# The *Barley stripe mosaic virus* γb protein promotes viral cell-to-cell movement by enhancing ATPase-mediated assembly of ribonucleoprotein movement complexes

**DOI:** 10.1371/journal.ppat.1008709

**Published:** 2020-07-30

**Authors:** Zhihao Jiang, Kun Zhang, Zhaolei Li, Zhenggang Li, Meng Yang, Xuejiao Jin, Qing Cao, Xueting Wang, Ning Yue, Dawei Li, Yongliang Zhang

**Affiliations:** State Key Laboratory of Agro-Biotechnology and Ministry of Agriculture Key Laboratory of Soil Microbiology, College of Biological Sciences, China Agricultural University, Beijing, P. R. China; Agriculture and Agri-Food Canada, CANADA

## Abstract

Nine genera of viruses in five different families use triple gene block (TGB) proteins for virus movement. The TGB modules fall into two classes: hordei-like and potex-like. Although TGB-mediated viral movement has been extensively studied, determination of the constituents of the viral ribonucleoprotein (vRNP) movement complexes and the mechanisms underlying their involvement in vRNP-mediated movement are far from complete. In the current study, immunoprecipitation of TGB1 protein complexes formed during *Barley stripe mosaic virus* (BSMV) infection revealed the presence of the γb protein in the products. Further experiments demonstrated that TGB1 interacts with γb *in vitro* and *in vivo*, and that γb-TGB1 localizes at the periphery of chloroplasts and plasmodesmata (PD). Subcellular localization analyses of the γb protein in *Nicotiana benthamiana* epidermal cells indicated that in addition to chloroplast localization, γb also targets the ER, actin filaments and PD at different stages of viral infection. By tracking γb localization during BSMV infection, we demonstrated that γb is required for efficient cell-to-cell movement. The N-terminus of γb interacts with the TGB1 ATPase/helicase domain and enhances ATPase activity of the domain. Inactivation of the TGB1 ATPase activity also significantly impaired PD targeting. *In vitro* translation together with co-immunoprecipitation (co-IP) analyses revealed that TGB1-TGB3-TGB2 complex formation is enhanced by ATP hydrolysis. The γb protein positively regulates complex formation in the presence of ATP, suggesting that γb has a novel role in BSMV cell-to-cell movement by directly promoting TGB1 ATPase-mediated vRNP movement complex assembly. We further demonstrated that elimination of ATPase activity abrogates PD and actin targeting of *Potato virus X* (PVX) and *Beet necrotic yellow vein virus* (BNYVV) TGB1 proteins. These results expand our understanding of the multifunctional roles of γb and provide new insight into the functions of TGB1 ATPase domains in the movement of TGB-encoding viruses.

## Author summary

Plant viruses employ varied movement strategies to mediate local and systemic infections. Viral ribonucleoprotein (vRNP) movement complexes comprising either the hordei- or potex-like triple gene block (TGB) and viral RNAs represent important models for plant virus movement. However, the constituents of viral ribonucleoprotein (vRNP) movement complexes as well as their biological significance in vRNP assembly and subsequent interactions are far from complete. Additionally, the mechanistic roles of the highly conserved TGB1 ATPase domain in vRNP-mediated movement remain an enigma. Here, we demonstrate that the γb protein acts as a novel positive regulator of BSMV cell-to-cell movement by directly interacting with the TGB1 protein. *In vitro* biochemical assays verified an essential role of TGB1 ATPase-mediated ATP hydrolysis in assembly of vRNP movement complexes, a process that can be further enhanced by the γb protein. We also extend our studies of BSMV TGB1 ATPase to those of PVX and BNYVV, and suggest a model for an evolutionally conserved mode of energy-coupled vRNP movement complex assembly among different TGB-encoding viruses. Our results address the knowledge gap between TGB1 ATPase activity and vRNP movement complex assembly and expand our understanding of the multifaceted roles of γb in BSMV infection.

## Introduction

Plant RNA viruses typically replicate on host endomembranes and require one or several movement proteins (MPs) for assembly and transport of progeny viral (v) RNAs to adjacent cells through plasmodesmata (PD) [1, 2]. A number of (+) ssRNA viruses share a conserved element consisting of three overlapping genes designated the “Triple gene block” (TGB) [3–8]. Based on the presence or absence of long N-terminal domains in TGB1 proteins and variable requirements of the coat protein (CP) for viral cell-to-cell movement, the TGB modules fall into two groups: hordei-like (class I) and potex-like (class II) [9–11]. Coordinated actions of the three TGB proteins are essential for viral ribonucleoprotein (vRNP) movement complex assembly and subsequent viral cell-to-cell movement [12–14]. However, despite extensive TGB studies, numerous critical steps in vRNP movement processes, such as switching of viral RNAs from replication templates and transition to key components of vRNP complexes for viral movement need to be clarified. In particular, the assembly and regulations of vRNP movement complexes, and recycling of plant-viral movement proteins by the endocytic pathway remain unclear at the molecular level.

**Fig 1:**
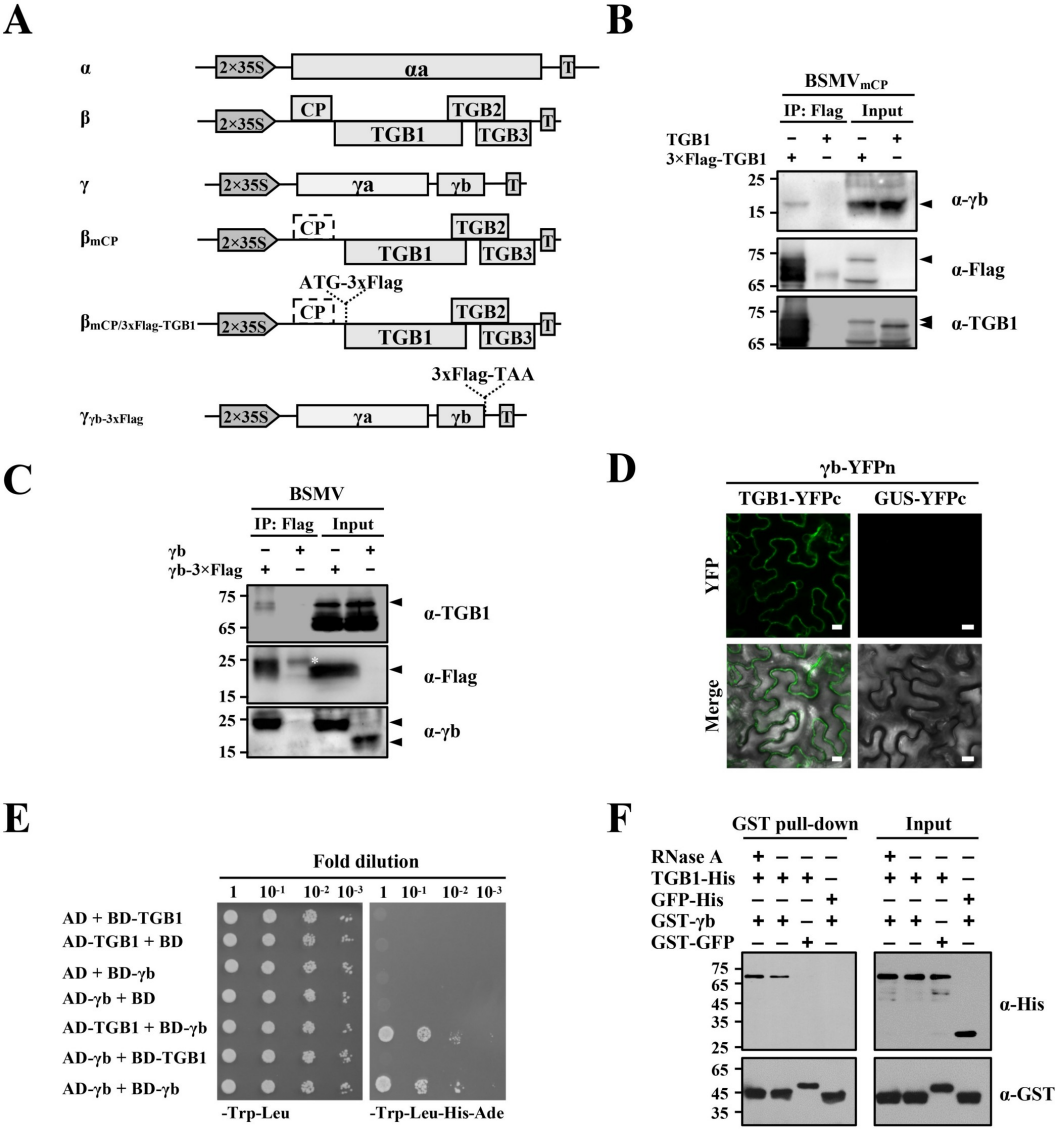
BSMV γb interactions with TGB1 *in vivo* and *in vitro*. **(A)** Schematic representation of BSMV infectious cDNA clones and their derivatives used for co-immunoprecipitation (Co-IP) analyses shown in Panels 1B and 1C. **(B and C)** Co-IP analyses to evaluate *in vivo* interactions between the γb and TGB1 proteins. A 3xFlag tag was engineered upstream of the TGB1 ORF in pCB301-β_mCP_ (B) or downstream of the γb ORF in the pCB301-γ (C). *N*. *benthamiana* leaves were infiltrated with *A*. *tumefaciens* harboring various constructs as indicated above the panels. BSMV negative controls consisted of pCB301-α, pCB301-β_mCP_, and pCB301-γ [76] without Flag tag insertions. Leaf tissues were harvested at 3 dpi. Total protein extracts were immunoprecipitated with anti-FLAG-agarose beads. Input and IP products were analyzed by Western blotting. Sizes (in kDa) of molecular weight markers are shown on the left and antibodies used for detection are on the right of each panel. Arrowheads and asterisk indicate the target and non-specific protein bands. **(D)** BiFC assays to test γb and TGB1 proteins interactions. *A*. *tumefaciens* harboring different plasmids expressing γb-YFPn/TGB1-YFPc or γb-YFPn/GUS-YFPc were co-infiltrated into *N*. *benthamiana* leaves. Combination of different constructs are shown above the panel. YFP signals were visualized by confocal microscopy at 3 dpi and depicted as a false-green color. Scale bars, 10 **μ**m. GUS-fused YFPc serves as a negative control. **(E)** Yeast two-hybrid (Y2H) assays to evaluate binding interactions between the γb and TGB1 proteins. Yeast cells transformed with the indicated plasmids were spotted onto dextrose dropout media (SD/-Trp-Leu or SD/-Trp-Leu-His-Ade) plates, in a series of 10-fold dilutions. Because of self-interactions of γb proteins, the γb gene was cloned as translational fusions with either AD or BD and used as positive controls, whereas the Y2H combinations containing either empty AD or BD constructs served as negative controls. **(F)** GST pull-down assays to analyze *in vitro* interactions between the γb and TGB1 proteins. His-tagged TGB1 or GFP was incubated with GST-γb or GST-GFP with or without the addition of 10 μg RNase A. Input and pull-down products were analyzed by Western blot analysis with anti-His or anti-GST antibodies.

*Barley stripe mosaic virus* (BSMV) is one of the two type members of the TGB-encoding viruses and has served as a model system for studies of virus movement for more than 30 years [4, 15]. BSMV contains three genomic RNAs designated RNAα, RNAβ, and RNAγ [15]. RNAα encodes the αa protein, which is the “helicase” subunit of the viral RNA dependent RNA polymerase (RdRp) complex. RNAβ encodes the coat protein (CP) in the first ORF, and is followed by three overlapping ORFs (TGB1, TGB2 and TGB3). The relative arrangement of the three genes is highly conserved in all TGB containing genera, including the allexi-, beny-, carla-, fovea-, peclu-, pomo-, and potexviruses [11, 16]. BSMV RNAγ encodes the γa protein, which is the “polymerase” subunit of the RdRp, and γb protein, which functions as a viral suppressor of RNA silencing (VSR) [17, 18]. Previously, we have inserted infectious cDNA clones representing each BSMV RNA into the binary vector pCB301 [19], to generate the pCB301-α, pCB301-β and pCB301-γ infectious clones [18] (Fig 1A). Local and systemic infection of BSMV in *Nicotiana benthamiana* can be established by co-infiltration of *Agrobacterium tumefaciens* harboring these and similar plasmids into plant leaves [18, 20].

BSMV TGB1 belongs to class I TGB proteins with large N-terminal extensions containing nucleolar and nuclear localization signals that are required for nucleocytoplasmic shuttling of TGB1 and cell-to-cell movement [21]. The C-terminal half of TGB1 contains an ATPase/helicase domain that belongs to superfamily I (SFI) helicases of alpha-like viruses with six conserved motifs (I, II, III, IV, V, and VI) [22]. BSMV TGB1 exhibits magnesium-dependent ATPase activity *in vitro* [22, 23]. Site-specific mutations in the conserved motifs alter TGB1 subcellular localization [24] and abrogate cell-to-cell movement [25]. TGB1 proteins also contain an NTPase/helicase domain that is responsible for NTP binding, hydrolysis of NTPs and unwinding of viral RNA duplexes [10, 26]. Mutation of conserved amino acids within the NTPase/helicase domains of TGB encoding viruses consistently results in interruption of cell-to-cell movement [27–29]. However, the underlying biochemical mechanisms whereby the NTPase/helicase domain functions in virus cell-to-cell movement are obscure.

BSMV sgβ2RNA encodes two small transmembrane proteins, TGB2 and TGB3, both of which are required for cell-to-cell movement [4, 24]. TGB3 interacts with both TGB1 and TGB2 *in vitro* and provides a basic lynchpin for BSMV ribonucleoprotein (RNP) interactions [13]. However, the extent to which other factors constitute the viral ribonucleoprotein (vRNP) movement complex and the biological significance of their involvement in vRNP assembly and subsequent movement need to be explored. Additionally, the mechanistic roles of the highly conserved TGB1 NTPase/helicase motifs in vRNP-mediated movement are still elusive.

The γb protein is translated from sub-genomic RNAγ (sgγ). Previous studies demonstrated that Hordeivirus γb’s are viral suppressors of RNA silencing (VSR) [17, 18]. γb also affects symptom development [30] and seed transmission of BSMV in barley [31] and complementation assays suggest that γb functions in long-distance movement of BSMV in *N*. *benthamiana* [32]. Our recent studies indicate that during early stages of BSMV infection, a majority of the γb proteins are recruited to the chloroplast outer membrane by binding to the αa replication protein subunit, and these interactions promote virus replication by enhancing unwinding of viral dsRNA intermediates [33]. In addition, γb interferes with ATG7-ATG8 interactions in a competitive manner to counteract autophagy-mediated antiviral defenses [34] and binds to glycolate oxidase (GOX) to suppress peroxisomal ROS bursts and promote BSMV infection [35]. The phosphorylated γb protein has a strong 21 bp dsRNA binding capacity and suppresses host cell death responses in *N*. *benthamiana*, wheat, and barley [36]. These studies *in toto* reveal multifunctional activities of the role of γb in BSMV pathogenesis.

In this study, we found that γb is a component of BSMV RNP movement complexes and positively regulates BSMV intracellular movement by directly interacting with the TGB1 protein. We also identified an orchestrated regulation mechanism underlying vRNP movement complex assembly that requires TGB1 ATPase-mediated ATP hydrolysis of TGB viruses and show that γb promotes this process by enhancing the ATPase activity. Our findings provide new insight into the movement of TGB-encoding viruses.

## Results

### γb interacts with TGB1 *in vivo* and *in vitro*

To identify host or viral factors that exist in TGB1 or γb protein complexes in *N*. *benthamiana*, we performed co-immunoprecipitation (co-IP) assays and analyzed the immunoprecipitates by liquid chromatography tandem mass spectrometry (LC-MS/MS). Because the CP is dispensable for cell-to-cell movement mediated by BSMV TGBs [37], RNAβ CP expression was eliminated by mutating the start codon of the CP ORF from AUG to UUG to produce RNAβ_mCP_). A 3xFlag epitope was then engineered as an N-terminal fusion to the TGB1 protein in RNAβ_mCP_, to generate RNAβ_mCP/3xFlag-TGB1_. *A*. *tumefaciens* strains harboring plasmids expressing RNAα, RNAβ_mCP/3xFlag-TGB1_, or RNAγ (Fig 1A) were co-infiltrated into *N*. *benthamiana* leaves and as a negative control leaves were co-infiltrated with mixtures of *A*. *tumefaciens* strains harboring plasmids expressing RNAα, RNAβ_mCP_, or RNAγ. At 3 days post-infiltration (dpi), infiltrated leaf discs were harvested, total proteins were extracted, immunoprecipitated with anti-FLAG-agarose beads and FLAG peptides were used to elute immunoadsorbed proteins from the beads. A small proportion of the IP products were separated by SDS-PAGE followed by silver staining, which indicated that substantial proteins were co-precipitated with the 3xFlag-TGB1 proteins compared with untagged TGB1 (S2 Fig). Subsequently, LC-MS/MS analyses were performed with the remaining eluates to determine the complexity of the proteins, and these results revealed αa, γb and TGB2 proteins (Table 1 and S2 Table), plus additional undetermined co-IP components. We also engineered a 3xFlag epitope fusion to the C-terminus of the γb protein to produce RNAγ_γb-3xFlag_, and *N*. *benthamiana* leaves were infiltrated with mixtures of *A*. *tumefaciens* harboring RNAα, RNAβ, or RNAγ_γb-3xFlag_ plasmids (Fig 1A). *N*. *benthamiana* leaves co-infiltrated with *A*. *tumefaciens* harboring plasmids expressing wild-type (wt) RNAα, RNAβ, or RNAγ served as a negative control. A Co-IP assay was performed at 3 dpi by using γb as a bait protein and subsequent LC-MS/MS analyses revealed the presence of TGB1, CP and some unidentified host factors amongst the immunoprecipitated products (Table 2 and S3 Table). These results indicate that TGB1 associates with γb during BSMV infection.

**Table 1:** Viral proteins identified by LC-MS/MS after immunoprecipitation of 3xFlag-TGB1 proteins from BSMV_mCP/3xFlag-TGB1_-infected *N*. *benthamiana*.

GenBank Accession	Score ^a^	Mass (Daltons)	Number of matches	Number of significant matches	Number of sequences	Number of significant sequences	emPAI	Sequence coverage (%)	Description
**AAA79149**	**21780**	**57438**	**490**	**476**	**17**	**17**	**3.35**	**38**	**BSMV TGB1**
**AAA79145**	**1839**	**131081**	**59**	**52**	**26**	**25**	**1.24**	**27**	**BSMV αa**
**2211403B**	**840**	**17859**	**33**	**29**	**7**	**6**	**3.04**	**27**	**BSMV γb**
**AAA79150**	**29**	**14311**	**2**	**2**	**2**	**2**	**0.78**	**11**	**BSMV TGB2**

^a^ Individual ions scores > 4 indicate identity or extensive homology (*p* < 0.05).

**Table 2:** Viral proteins identified by LC-MS/MS after immunoprecipitation of γb-3xFlag proteins from BSMV_γb-3xFlag_-infected *N*. *benthamiana*.

GenBank Accession	Score ^a^	Mass (Daltons)	Number of matches	Number of significant matches	Number of sequences	Number of significant sequences	emPAI	Sequence coverage (%)	Description
**2211403B**	**34228**	**17776**	**854**	**778**	**20**	**15**	**41.39**	**83**	**BSMV γb**
**AAA79148**	**2133**	**22513**	**74**	**63**	**10**	**10**	**5.39**	**63**	**BSMV CP**
**AAA79149**	**152**	**58458**	**13**	**6**	**7**	**3**	**0.24**	**17**	**BSMV TGB1**

^a^ Individual ions scores > 27 indicate identity or extensive homology (*p* < 0.05).

To test TGB1 interactions with BSMV γb, co-IP analyses were performed at 3 dpi using the same experimental conditions as in S2 Fig. *N*. *benthamiana* leaves were co-infiltrated with *A*. *tumefaciens* containing pCB301-α, pCB301-γ, as well as pCB301-β_mCP/3xFlag-TGB1_ or pCB301-β_mCP_, and subjected to co-IP assays with anti-FLAG beads. In contrast to prior co-IP assays, whose immunoprecipitated products were used for LC-MS/MS analysis, the resulting immunoprecipitates from this round of co-IP experiments were subjected to Western blot analysis with specific antibodies against either TGB1 or γb (Fig 1B). The results showed that γb co-precipitated with 3xFlag tagged TGB1, but not with the untagged TGB1 protein (Fig 1B). *A*. *tumefaciens* harboring plasmids expressing RNAα, RNAβ, as well as RNAγ or RNAγ_γb-3xFlag_ were co-infiltrated into *N*. *benthamiana* leaves, and the result showed that the γb-3xFlag protein co-precipitated with TGB1, whereas the untagged γb did not (Fig 1C). We then performed biomolecular fluorescence complementation (BiFC) assays to further evaluate γb-TGB1 interactions with *A*. *tumefaciens* containing plasmids expressing γb-YFPn and TGB1-YFPc or GUS-YFPc. Reconstitution of YFP signals was observed in *N*. *benthamiana* epidermal cells co-infiltrated with the γb-YFPn/TGB1-YFPc combinations (Fig 1D, left panels). However, discernable fluorescence signals were not evident when γb-GFP was co-expressed with the GUS-YFPc negative control (Fig 1D, right panels), and Western blot analyses confirmed expression of TGB1 and γb proteins in the infiltrated leaves (S3 Fig). We also performed yeast two-hybrid (Y2H) assays to examine interactions between γb and TGB1. Our results showed that γb failed to interact with TGB1 in the Y2H assay as evidenced by the absence of yeast colonies when the AD-γb (γb fused to the Gal4 activation domain) was paired with the BD-TGB1 (TGB1 fused to the Gal4 DNA binding domain), which is in agreement with a previous study [17]. However, when we exchanged the AD and BD fusions by pairing the BD-γb with AD-TGB1, growth of yeast colonies was evident on SD/-Trp-Leu-His-Ade drop-out plates (Fig 1E), in contrast to that of the AD-γb and BD-TGB1 combinations, suggesting that positional effect of the AD or BD fusions affect γb-TGB1 interactions in the Y2H system. Nevertheless, these results suggest that TGB1 interacts with γb *in vivo*.

To further investigate whether γb interacts directly with TGB1, we performed GST pull-down assays with recombinant proteins purified from *E*. *coli*. Due to the strong ssRNA binding activities of both γb and TGB1 [22, 38], RNase A was added to one of the experimental groups during incubation to exclude the potential role of RNA in mediating the γb-TGB1 interactions. The results showed that GST-γb specifically pulled down the TGB1-His protein in an RNA-independent manner. In contrast, TGB1 specific bands were absent in the GST-GFP pull-down products, and GST-γb failed to pull-down GFP-His (Fig 1F). These results provide additional evidence confirming that γb interacts physically with TGB1 *in vitro*.

We also tested binding between γb and TGB2 or TGB3 by using Y2H and BiFC assays. The Y2H results were negative for both TGB2 and TGB3 interactions with γb (S4 Fig). Similarly, confocal analysis of *N*. *benthamiana* leaves infiltrated with the *A*. *tumefaciens* carrying plasmids expressing either γb-YFPn/TGB3-YFPc or γb-YFPc/TGB3-YFPn failed to generate fluorescence signals. However, in BiFC analyses of the γb-TGB2 interaction, the γb-YFPc and TGB2-YFPn combination produced several fluorescence punctate spots, but the complementary (γb-YFPn and TGB2-YFPc) pairing failed to reconstitute YFP fluorescence (S5 Fig). These results further demonstrate specific interactions between γb and TGB1, but do not provide unequivocal evidence for TGB2 or TGB3 interactions with γb.

### γb localizes to multiple subcellular sites during BSMV infection

**Fig 2:**
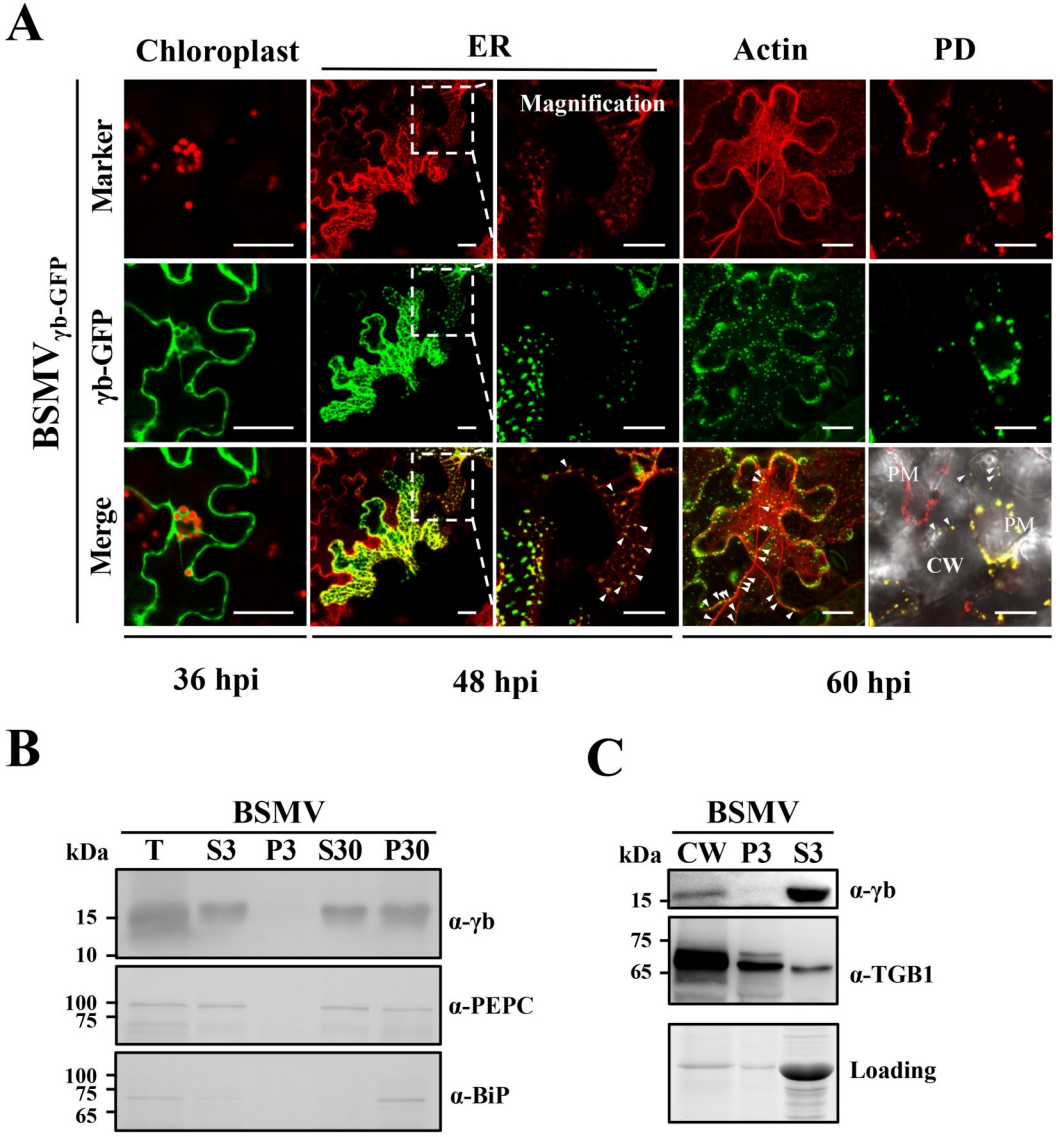
γb localization to multiple subcellular sites during BSMV infection. **(A)** Confocal analyses of γb subcellular localization during BSMV infection. GFP was engineered downstream of the γb ORF in the pCB301-γ (S1 Fig). *N*. *benthamiana* epidermal cells were co-infiltrated with *A*. *tumefaciens* harboring pCB301-α, pCB301-β or pCB301-γ_γb-GFP_, and confocal analyses were conducted at different time points as indicated below the panels. Chloroplasts were visualized by chlorophyll fluorescence and mCherry-HDEL [39], DsRed:Talin [28], and CFP-PDLP [21] were used as markers to indicate the ER, actin, and plasmodesmata (PD), and these organelles are displayed as a false red color. The white dotted boxes inset the left ER panels were further magnified to clearly show the subcellular localization of γb. White arrowheads in the right ER panels indicate fluorescence puncta located in close proximity to the ER network. The white arrowheads in the Actin and PD panels highlight colocalization of γb with thickened actin filaments and PDs. Scale bars, 20 μm. PM, plasma membrane. **(B)** Western blot analyses of subcellular fractions extracted from *N*. *benthamiana* leaf tissue infected with BSMV at 3 dpi. The sizes (in kDa) of molecular weight markers are shown on the left and antibodies used for detection are on the right of each panel. Phosphoenolpyruvate carboxylase (PEPC) and the luminal binding protein (BiP) were used as markers for the soluble and membrane fractions. T, total protein extracts; S3, supernatant separated by 3000 *g* centrifugation; P3, pellet separated by 3000 *g* centrifugation; S30, supernatant separated by 30000 *g* centrifugation; P30, pellet separated by 30000 *g* centrifugation. **(C)** Western blot detection of γb protein subcellular localization after CW fractionation of *N*. *benthamiana* tissue infected with BSMV at 3 dpi. The lower panel shows the amounts of proteins used for loading controls. Molecular weight markers are shown on the left and the antibodies used for detection are shown on the right of each panel. The TGB1 protein served as a CW marker. CW, cell wall fraction; S3, supernatant separated by 3000 *g* centrifugation; P3, pellet separated by 3000 *g* centrifugation.

We previously reported that γb localizes to chloroplasts early in BSMV infections [33]. To obtain additional information about the involvement of γb in infection, we carried out time-course observations of γb subcellular localization at different times after BSMV inoculation of *N*. *benthamiana*. *A*. *tumefaciens* derivatives harboring pCB301-α, pCB301-β and pCB301-γ_γb-GFP_ (BSMV_γb-GFP_, S1 Fig) were co-infiltrated into leaves followed by periodic confocal analyses after infiltration. This system permits a kinetic analysis of γb protein subcellular localization throughout various stages of BSMV infection. At 36 hours post infiltration (hpi), chloroplast localization of γb [33] was readily observed (Fig 2A, Chloroplast panels), and by 48 hpi, γb-GFP punctate foci were detected in some infected cells within a dense ER network visualized by the mCherry-HDEL ER marker [39] (Fig 2A, middle ER panels), or in close proximity to the ER (Fig 2A, right ER panels), and some of the bodies appeared to move along the ER network (S1 Video). Because the ER network is always closely associated with the actin cytoskeleton in plants [40], and previously studies have shown that BSMV infection is strongly associated with the ER/actin network [41], we presumed that the puncta were co-localizing with actin filaments. To confirm this notion, *A*. *tumefaciens* harboring plasmids encoding the actin marker DsRed:Talin [28] and BSMV_γb-GFP_ infectious RNAs were co-infiltrated into *N*. *benthamiana* epidermal cells. At 60 hpi, we observed an apparent reorganization and thickening of actin filaments that occurs during BSMV infection, as shown earlier by Lim *et al*. [41]. In addition, γb-GFP punctate fluorescence foci co-localized with the thickened RFP actin filament bundles, along with some punctate bodies at the cell periphery (Fig 2A, Actin panels). To further determine γb targeting to the PD during infection, we plasmolyzed *N*. *benthamiana* epidermal leaf cells that had been co-infiltrated with *A*. *tumefaciens* expressing BSMV_γb-GFP_ infectious RNA plasmids and the PD marker CFP-PDLP [21]. After plasmolysis, some of the γb-GFP punctate bodies were retained at the cell wall (CW) and colocalized closely with the PD marker (Fig 2A, PD panels). These results provide strong visual evidence that γb associates with both actin filaments and the PD, and extend the previous results of Lim *et al*. [41].

To further evaluate the distribution of γb during BSMV infection shown in Fig 2A, a procedure to separate cellular fractions by filtration and differential centrifugation was performed. Western blot analyses of protein extracts from the fractions revealed the presence of γb in both the soluble protein (S30) and membrane-enriched subcellular pellet (P30) fractions (Fig 2B), and showed that γb and TGB1 cofractionated in the CW-enriched P30 pellet containing cell walls and PD (Fig 2C). Considering the functional roles of the ER/actin network in plant virus movement [1, 42], the ER, actin, and PD localization of γb as shown above suggests that γb functions at multiple sites during BSMV replication and movement.

### TGB2 and TGB3 interact cooperatively to determine PD targeting of γb-TGB1 complexes during BSMV infection

**Fig 3:**
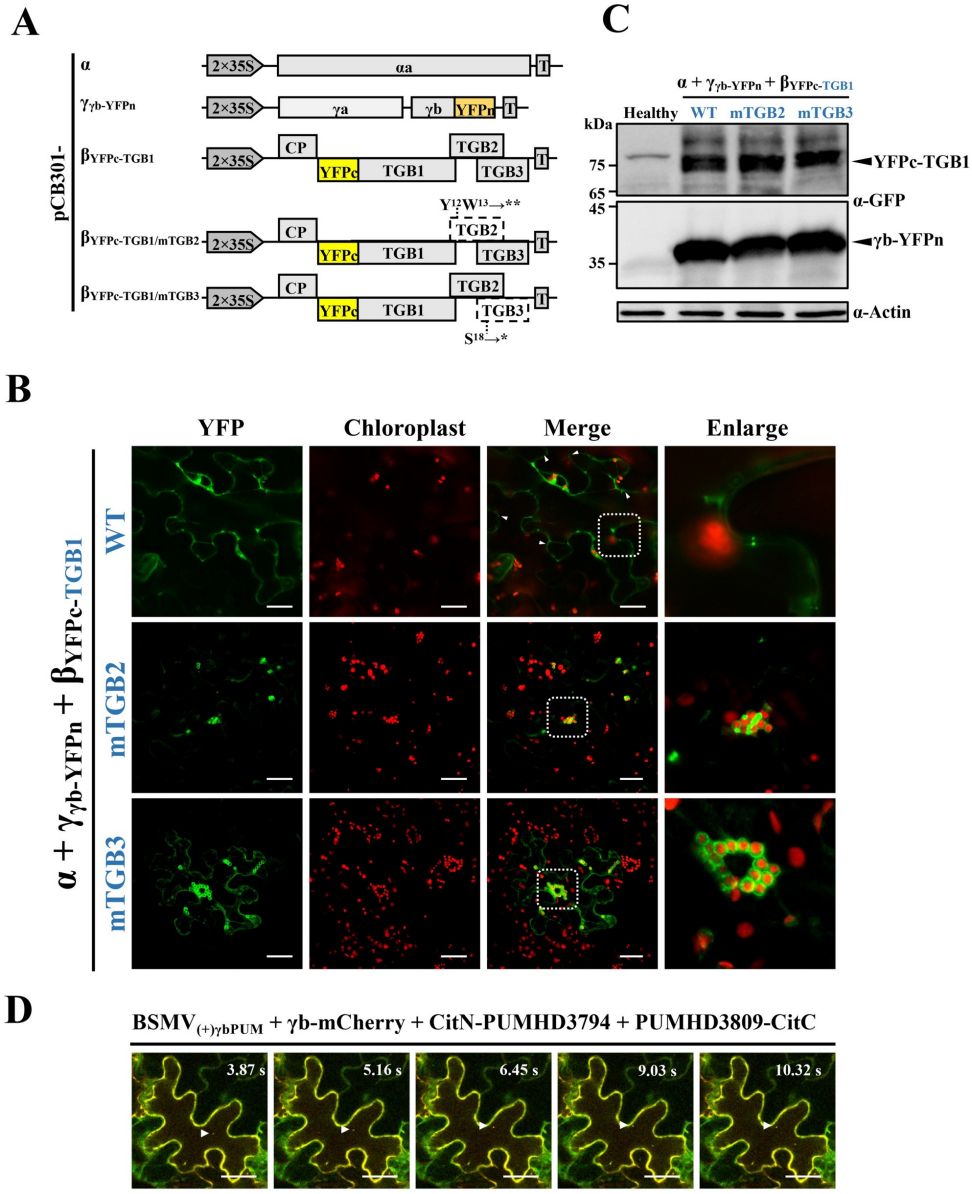
γb multi-subcellular site interactions with TGB1 and mobile granules associated with BSMV RNAs during infection. **(A)** Schematic representation of the BSMV-based BiFC system and TGB2 and TGB3 deficient mutants used for analyses of γb-TGB1 interactions. The asterisks indicate the stop codon. T, terminator. **(B)** BiFC confocal microscopy visualization of BSMV γb and TGB1 protein interactions. Co-infiltration of *A*. *tumefaciens* strains harboring pCB301-α and pCB301-γ_γb-YFPn_ with pCB301-β_YFPc-TGB1_ (top panels), pCB301-β_YFPc-TGB1/mTGB2_ (middle panels), or pCB301-β_YFPc-TGB1/mTGB3_ (bottom panels) in *N*. *benthamiana* leaves. YFP signals depicted as a false-green color at 3 dpi with red chloroplast autofluorescence. The white dotted boxed insets of the merged panels were magnified to highlight associations of reconstituted YFP signals with PD (top panels) and chloroplasts (middle and bottom panels). The white arrowheads indicate putative PD sites containing γb-TGB1 protein interactions. Scale bars, 25 μm. **(C)** Western blot with anti-GFP antibodies confirming protein expression in agroinfiltrated *N*. *benthamiana* leaves. Arrowheads indicate target bands corresponding to YFPc-TGB1 (~76 kDa) and γb-YFPn (~36 kDa) expressed from the BSMV infectious clone. Actin antibodies used to monitor protein loading (bottom panel). Sizes (in kDa) of molecular weight markers are shown on the left and antibodies used for detection are shown on the right of each panel. **(D)** Time-lapse confocal microscopy analyses of relationships between γb protein and vRNAs. *A*. *tumefaciens* harboring plasmids expressing RNAα, RNAβ, or RNA_(+)γbPUM_ were co-infiltrated into the six-leaf stage of *N*. *benthamiana* epidermal cells and fifteen days later, upper systemically infected leaves were agroinfiltrated with *A*. *tumefaciens* harboring plasmids expressing γb-mCherry, CitN-PUMHD3794, and PUMHD3809-CitC as described previously [33]. The infiltrated leaves were visualized at 3 dpi by confocal microscopy. White arrowheads indicate movement of fluorescent granules in the cytosol. Scale bars, 25 μm. The corresponding video was available in S2 Video.

Previous studies indicated that BSMV TGB1 is the major component of vRNP movement complexes [13]. To further characterize γb-TGB1 interactions during BSMV infection, subcellular localizations of γb-TGB1 complexes were analyzed by using of a BSMV-based BiFC system. Briefly, the C- or N-terminal halves of YFP fragments were inserted into the TGB1 N-terminal region or the γb C-terminal region to generate pCB301-β_YFPc-TGB1_ and pCB301-γ_γb-YFPn_ constructs (Fig 3A). With this system, reconstituted YFP fluorescence emitted by γb-TGB1 associations reflects the native subcellular localization of γb-TGB1 complexes during BSMV infection and enhances the transient overexpression-based BiFC assays shown in Fig 1C. After co-infiltrated of *N*. *benthamiana* leaves with *A*. *tumefaciens* derivatives containing plasmids expressing RNAα, RNAβ_YFPc-TGB1_, or RNAγ_γb-YFPn_, reconstituted YFP fluorescence was observed in the cytoplasm of epidermal cells (Fig 3B, upper panels). Notably, many punctate fluorescent foci appeared on opposite sides of the CW (Fig 3B, upper panels), in agreement with γb-GFP PD localization during BSMV infection (Fig 2A).

To investigate whether the TGB2 or TGB3 determines subcellular localization of γb-TGB1 complexes, two movement-deficient mutants, pCB301-β_YFPc-TGB1/mTGB2_ and pCB301-β_YFPc-TGB1/mTGB3_, were constructed by substitution of UAUUGG for UAAUAG at nts 2614–2619 or UCG for UAG at nts 2837–2839 of RNAβ_YFPc-TGB1_ to produce premature termination of the TGB2 or TGB3 ORFs. In pCB301-β_YFPc-TGB1/mTGB3_ mutant, the codons for isoleucine (nt 2836–2838) and glycine (nt 2839–2841) in the TGB2 ORF were maintained after the nucleotide substitutions (Fig 3A). *N*. *benthamiana* leaves were infiltrated with *A*. *tumefaciens* harboring pCB301-α, pCB301-β and pCB301-β_YFPc-TGB1/mTGB2_ or pCB301-β_YFPc-TGB1/mTGB3_. The results showed that the punctate bodies located at the cell periphery disappeared, but that strong YFP fluorescence was still present at the chloroplast periphery (Fig 3B, the middle and bottom panels). Expression of YFPc-TGB1 and γb-YFPn proteins in the agroinfiltrated leaves was confirmed by immunoblot analyses (Fig 3C). These results thus suggest that γb functions as a component of vRNP movement complexes by interacting with TGB1 and is cooperatively regulated by TGB2 and TGB3 interactions during BSMV infection.

### γb and BSMV RNAs form mobile granules in infected *N*. *benthamiana* cells

To assess the relationship of γb with vRNAs in leaf cells, we previously developed an RNA imaging system (PUM-BiFC) that can be used with BSMV_(+)γbPUM_ (pCB301-α, pCB301-β and pCB301-γ_(+)γbPUM_) infections [33]. The two target sites specifically recognized by two Pumilio homology domain (PUMHD) polypeptides were engineered downstream of the γb stop codon in the plus sequence orientation (S1 Fig). These two PUMHD proteins were individually fused to either the N- or C-terminal halves of split mCitrine (CitN-PUMHD3794 and PUMHD3809-CitC) [43]. Lower *N*. *benthamiana* leaves were first co-infiltrated with *A*. *tumefaciens* derivatives containing the BSMV_(+)γbPUM_ infectious clone and about fifteen days later, when the upper uninoculated leaves were systemically infected with BSMV_(+)γbPUM_, *A*. *tumefaciens* derivatives containing plasmids expressing CitN-PUMHD3794 and PUMHD3809-CitC were co-infiltrated into the upper leaves. We anticipated that upon expression, the two PUMHDs proteins would bind to their specific target sites in RNAγ molecules to result in reconstitution of mCitrine fluorescence and permit observations of RNAγ localization in the infected cells. To investigate whether RNAγ associates with γb, the BSMV_(+)γbPUM_ systemically infected leaves were co-infiltrated with *A*. *tumefaciens* harboring plasmids expressing γb-mCherry, CitN-PUMHD3794, or PUMHD3809-CitC. Red fluorescence representing γb-mCherry co-localized with the reconstituted mCitrine BSMV RNAγ fluorescent signals, and some of the merged fluorescence foci were observed to move rapidly in the cytoplasm (Fig 3D and S2 Video). These results thus reveal an intimate association of γb with RNAγ to produce complexes that can be transported intracellularly as mobile granules during infection.

### γb is required for efficient BSMV cell-to-cell movement

**Fig 4:**
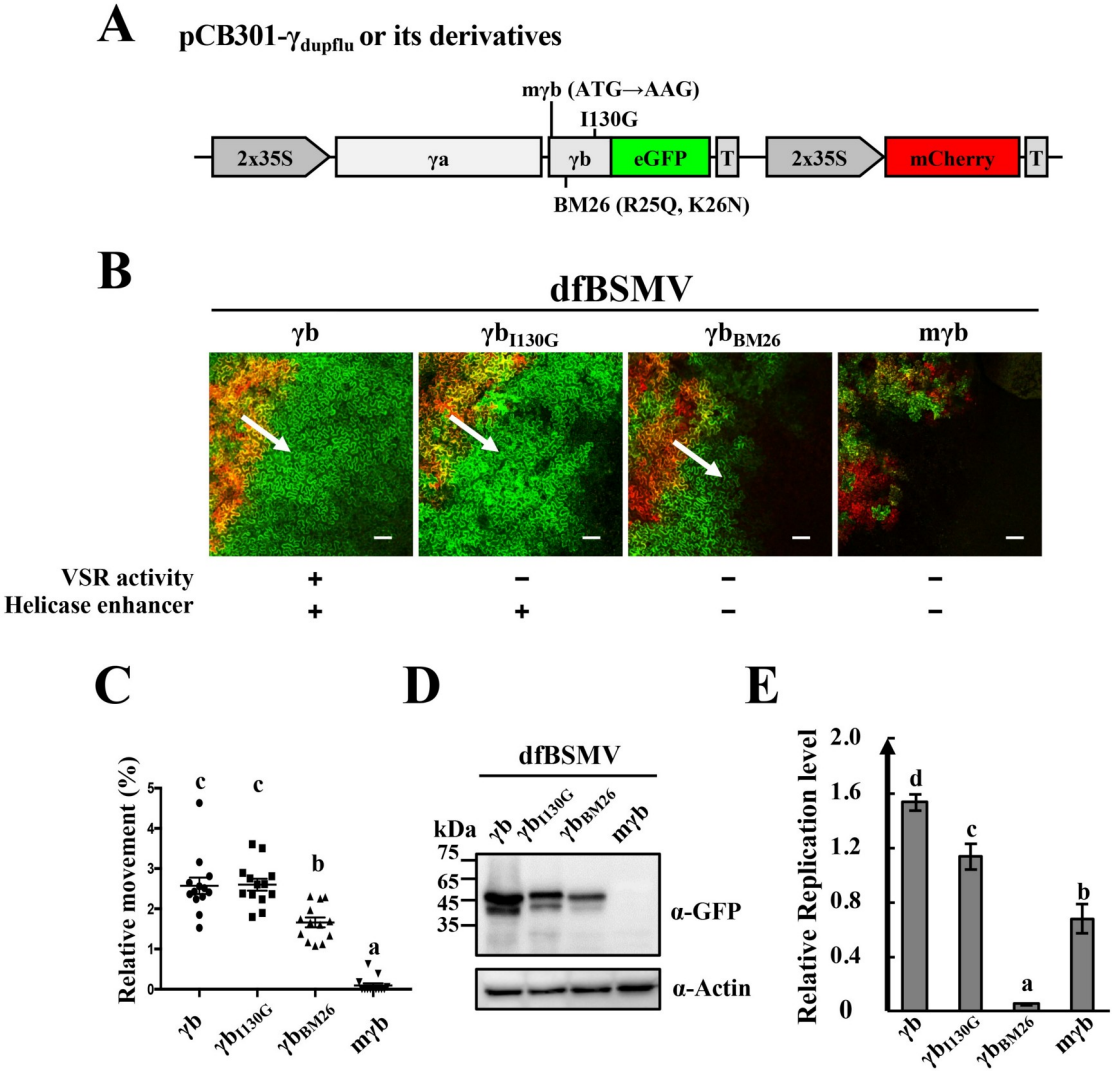
Requirement of γb for efficient BSMV cell-to-cell movement. **(A**) Schematic representation of wild-type RNAγ and derivatives containing γb mutants used for the dfBSMV reporter system [21]. **(B)** Analyses of wild-type RNAγ and different γb mutant cell-to-cell movement with the dfBSMV reporter system. VSR or helicase enhancer activities that were retained (+) or inactivated (−) in γb are indicated below the panels. Red fluorescence produced from the mCherry expression cassette shows primary infiltrated areas and the BSMV GFP reporter fluorescence outside the red region identifies secondary tissue invasion. White arrows indicate the direction of infection movement. Scale bars, 75 μm. **(C)** Quantification of BSMV movement shown in Figure 4B. The areas of green and red fluorescence were measured by ImageJ software (n = 13). Y-axis indicates the relative sizes of the green areas in comparison to that of the red-colored areas. Different letters in the chart denote statistically significant differences among different groups according to the Duncan’s multiple range test (*P* < 0.05). **(D)** Western blot antibody detection of GFP accumulation in green areas surrounding the infiltrated regions shown in Figure 4B. Leaf samples for Western blot analysis were excised under a Leica stereo fluorescent microscope to avoid leaf tissue contamination from the infiltrated areas. Equal protein loading was monitored by the actin protein (bottom panel). Sizes (in kDa) of molecular weight markers are shown on the left and antibodies used for detection are shown on the right. **(E)** RT-qPCR analyses of wild-type BSMV and γb mutant replication. *A*. *tumefaciens* pCB301-α and pCB301-γ or different pCB301-γ mutant co-infiltrations of *N*. *benthamiana* leaves at 3 dpi. Total RNA was isolated and the amounts of RNAα were determined by RT-qPCR to evaluate replication [33]. Letters above the bars denote significant differences (*P* < 0.05) according to the Duncan’s multiple range test (n = 3).

Because the BSMV TGB block is responsible for virus movement and γb directly interacts with TGB1, we next examined whether γb functions in BSMV movement. For this purpose, we developed a BSMV duplex fluorescence (dfBSMV) reporter system based on one described in our previous study [21]. In this system, an mCherry expression cassette was inserted downstream of RNAγ_γb-GFP_ to produce the pCB301-γ_dupflu_ plasmid that enables transcription of the mCherry RNA only in *Agrobacterium* containing cells (Fig 4A). *N*. *benthamiana* leaves were infiltrated with *A*. *tumefaciens* harboring pCB301-α, pCB301-β or pCB301-γ_dupflu_ plasmids (designated dfBSMV) and the borders of the infiltrated regions were marked on the leaves. As expected, only the primary agroinfiltrated tissue expressed the mCherry red color (Fig 4B), whereas the γb-GFP protein encoded by sgRNAγ emitted green fluorescence in both the primary infection foci and in the surrounding secondary tissue invaded during cell-to-cell movement. At least seven leaf tissues located at the border of the infiltration regions were harvested at different time points and observed by confocal microscopy.

To investigate the effects of γb on BSMV cell-to-cell movement, the expression of γb in pCB301-γ_dupflu_ was eliminated by mutating the start codon of γb from AUG to UUG, to generate γb-deficient RNAγ_dupflu_ (pCB301-γ_mγbdupflu_, Fig 4A). *A*. *tumefaciens* harboring plasmids expressing RNAα, RNAβ, RNAγ_dupflu_ or RNAγ_mγbdupflu_ were co-infiltrated into *N*. *benthamiana* leaves. The results reveal that both wtBSMV and the BSMV_mγb_ mutants replicated in the infiltrated region, but obvious cell-to-cell movement was not observed at 2 dpi (S6 Fig). At 3 dpi, BSMV_mγb_ was restricted to the infiltrated area and only sporadic expression of GFP was observed in surrounding cells, in striking contrast to that of wtBSMV invaded tissue, which had obvious GFP fluorescence outside the red-colored infiltration region (Fig 4B and 4C, S6 Fig). At 5 dpi, very few cells exhibited GFP fluorescence were present outside the infiltrated regions and obvious cell-to-cell movement could be visualized in less than 5% of the observed fields (S6 Fig). These results suggest that γb is required but is not absolutely essential for BSMV cell-to-cell movement in *N*. *benthamiana*.

To dissect the potential functional roles of γb in virus movement from those of virus replication or viral suppression of RNA silencing (VSR) as described previously [17, 33], we used BSMV derivatives containing γb loss-of-function mutants to analyze virus movement (Fig 4A). The first mutant designated pCB301-γ_I130Gdupflu_ contained a single amino acid substitution (I130G) in γb (Fig 4A) that causes considerably reduced VSR activity [33], and confocal analyses indicated that the cell-to-cell movement ability of this mutant was not dramatically impaired because movement was comparable to that of wtBSMV (Fig 4B and 4C). The pCB301-γ_BM26dupflu_ mutant (Fig 4A), in which both the VSR activity and helicase enhancement activity of γb had been destroyed was used for confocal analysis. The pCB301-γ_BM26dupflu_ results indicated that the cell-to-cell movement of dfBSMV_BM26_ was significantly more extensive than dfBSMV_mγb_ (Fig 4B and 4C). The movement of different mutants was also assessed by Western blot analysis of the GFP protein abundance in areas outside the infiltration region. The results revealed that the γb_I130G_ and γb_BM26_ mutants were able to move outside the infiltrated region and generate GFP fluorescence, albeit marginally weaker than wtBSMV. In contrast, dfBSMV_mγb_ exhibited markedly reduced cell-to-cell movement, with GFP accumulation below the Western blot detection limits (Fig 4D).

RNA replication of different BSMV derivatives as accessed by varied amounts of BSMV RNAα was analyzed by RT-qPCR at 3 dpi [33], and the results indicated that the γb mutants had strikingly different effects on replication (Fig 4E). The BM26 mutant lacking VSR activity had a more severe impact on BSMV replication than the mγb mutants without VSR or helicase enhancement activity, which we hypothesize may be related to dominant-negative effects on replication [33]. Despite lower replication levels, the BM26 mutant was more active in cell-to-cell movement than the mγb mutant (Fig 4B and 4C). Altogether, these results demonstrate that γb has a positive role in BSMV cell-to-cell movement that is separate from its VSR and helicase enhancer activities.

### Multiple regions of γb and TGB1 are required for γb-TGB1 interactions

**Fig 5:**
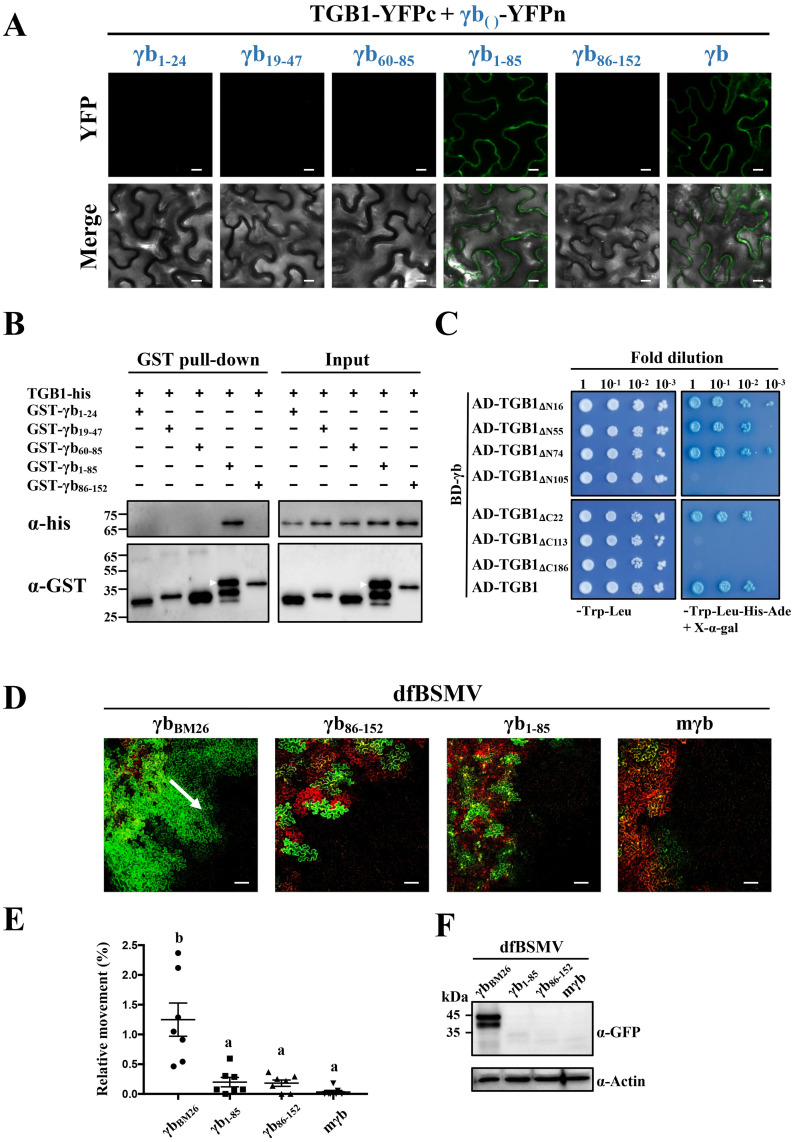
Analyses of regions responsible for γb and TGB1 protein interactions. **(A)** BiFC analyses of TGB1 and truncated γb protein mutants. Co-expression of the N-terminal halves of YFP-fused γb or truncated derivatives and TGB1-YFPc under control of the 35S promoter in *N*. *benthamiana* leaves. The subscript numbers show γb amino acids used for BiFC assays. YFP signals were visualized by confocal microscopy at 3 dpi. Scale bars, 10 μm. **(B)** GST pull-down assays of interactions between TGB1 and different truncated γb mutant proteins. The His-tagged TGB1 protein was incubated with different GST-tagged γb variants. After incubation with glutathione agarose beads, the pull-down products were analyzed by Western blotting with anti-His or anti-GST antibody. Sizes (in kDa) of molecular weight markers and the antibodies used for detection are shown on the left. The white arrows indicate the target bands. **(C)** Y2H assays of yeast transformants expressing γb or truncated TGB1 mutants as BD or AD fusions. Various combinations of yeast two-hybrid vectors are indicated on the left. A dilution series (10^−1^, 10^−2^, 10^−3^, and 10^−4^) of yeast cells were spotted on yeast synthetic drop-out media (SD/-Trp-Leu or SD/-Trp-Leu-His-Ade) supplemented with X-α-Gal. Interactions of γb and TGB1 confirmed by Y2H assays serve as a positive control. **(D)** Confocal Analyses of cell-to-cell movement of BSMV γb mutant derivatives. *A*. *tumefaciens* harboring pCB301-α, pCB301-β or various pCB301-γ-derivative constructs were co-infiltrated into the *N*. *benthamiana* leaves, and analyses were performed at 3 dpi. The dfBSMV_BM26_ mutant is a control. Scale bars, 100 μm. The white arrow indicates the direction of BSMV movement. **(E)** Quantification of BSMV movement efficiency shown in panel D. The green (infiltrated and peripheral BSMV invaded regions) and red fluorescent (*Agrobacterium* infiltrated regions) areas were measured by ImageJ software (n = 7). The Y-axis indicates the relative sizes of the green areas in comparison with the red-colored areas. Different letters in the chart denote statistically significant differences among different groups according to the Duncan’s multiple range test (*P* < 0.05). **(F)** Western anti-GFP antibody blotting to detect GFP accumulation in leaf regions adjacent to the red-colored areas shown in Figure 5D. Actin immunoblots shown below are loading controls. Leaf samples for Western blots were excised under a Leica stereo fluorescent microscope as in Fig 4D. Sizes (in kDa) of molecular weight markers are shown on the left and antibodies used for detection are shown on the right of each panel.

To determine the regions responsible for interactions of γb with TGB1, a series of γb truncation mutants were constructed for BiFC assays. Each γb mutant was fused with the N-terminal half of YFP and co-expressed with TGB1-YFPc. As a positive control, BiFC analysis confirmed the wtγb-TGB1 interactions (Fig 5A, right panels). In contrast, confocal analysis of the mutant γb-TGB1 pairs revealed that only the N-terminal 1–85 amino acids (aa) of γb interact with TGB1, and that each internal domain of the N-terminus, including C1 (consisting of aa 1–24 of γb, γb_1-24_), BM (consisting of aa 19–47 of γb, γb_19-47_), and C2 (consisting of aa 60–85 of γb, γb_60-85_), are required for interactions with TGB1 as evidenced by the BiFC assay (Fig 5A). Western blot analysis confirmed expression of the target proteins in the infiltrated leaves (S7 Fig), and we also performed GST pull-down assay using recombinant proteins purified from *E*. *coli*. Our results showed that only the GST-fused N-terminal γb (GST-γb_1-85_) mutant specifically pulled down the TGB1-His protein, whereas other truncations of the γb protein did not (Fig 5B). Taken together, these results indicate that the N-terminus of γb (γb_1-85_) is required for TGB1 binding.

To identify regions within TGB1 that are required for γb-TGB1 interactions, we constructed a series of TGB1 deletion mutants for Y2H assays. BD-γb was tested for its possible interactions with various TGB1 truncation mutant fusions with the GAL4 activation domain as shown in Fig 5C. As a positive control, Y2H analyses confirmed that the γb-TGB1 interactions are consistent with the BiFC results shown in Fig 5A. Yeast expressing the AD-TGB1_ΔN74_ containing the C-terminal 75–512 aa of TGB1 grew well on SD/-Trp-Leu-His-Ade drop-out plates after mating with yeast containing the BD-γb expression constructs, whereas deletion of the TGB1 N-terminal 105 aa led to failure of yeast growth after mating (Fig 5C, upper right panel). Truncations of the TGB1 C-terminal sequences indicated that TGB1 aa 1–491 retained the ability to interact with γb, but that C-terminal TGB1 deletions to aa 113 or aa 186 destroyed TGB1 binding to γb (Fig 5C, bottom right panel). These data indicate that TGB1 aa 75 to 491 are required for γb-TGB1 binding.

We next used the dfBSMV reporter system to assess whether the mapped γb region responsible for γb-TGB1 interactions contributes to BSMV cell-to-cell movement. The pCB301-γ_1-85dupflu_ and pCB301-γ_86-152dupflu_ derivatives that express aa 1–85 and aa 86–152 of γb were constructed, and *N*. *benthamiana* leaves were co-infiltrated with *A*. *tumefaciens* containing pCB301-γ_dupflu_ or its derivatives containing different γb mutations. As a positive control, dfBSMV_BM26_ had only minor reductions in cell-to-cell movement (Fig 5D, left panel). In contrast, dfBSMV_γb86-152_ (pCB301-α + pCB301-β + pCB301-γ_86-152dupflu_) resulted in significantly reduced cell-to-cell movement, similar to that of the dfBSMV_mγb_ control (Fig 5D and 5E). Nevertheless, dfBSMV_γb1-85_ (pCB301-α + pCB301-β + pCB301-γ_1-85dupflu_) was unable to rescue cell-to-cell movement of BSMV (Fig 5D and 5E). Western blot analyses of total protein extracts from leaf regions outside the infiltration area had GFP expression levels similar to those of the GFP fluorescence intensity shown in Fig 5D (Fig 5F). These results indicated that although the N-terminus of γb (aa 1–85) retains the ability to interact with TGB1, it is not sufficient to support BSMV intercellular movement, and that the γb C-terminal 86–152 aa are also required for movement.

### TGB1 ATPase activity is essential for actin and PD targeting

**Fig 6:**
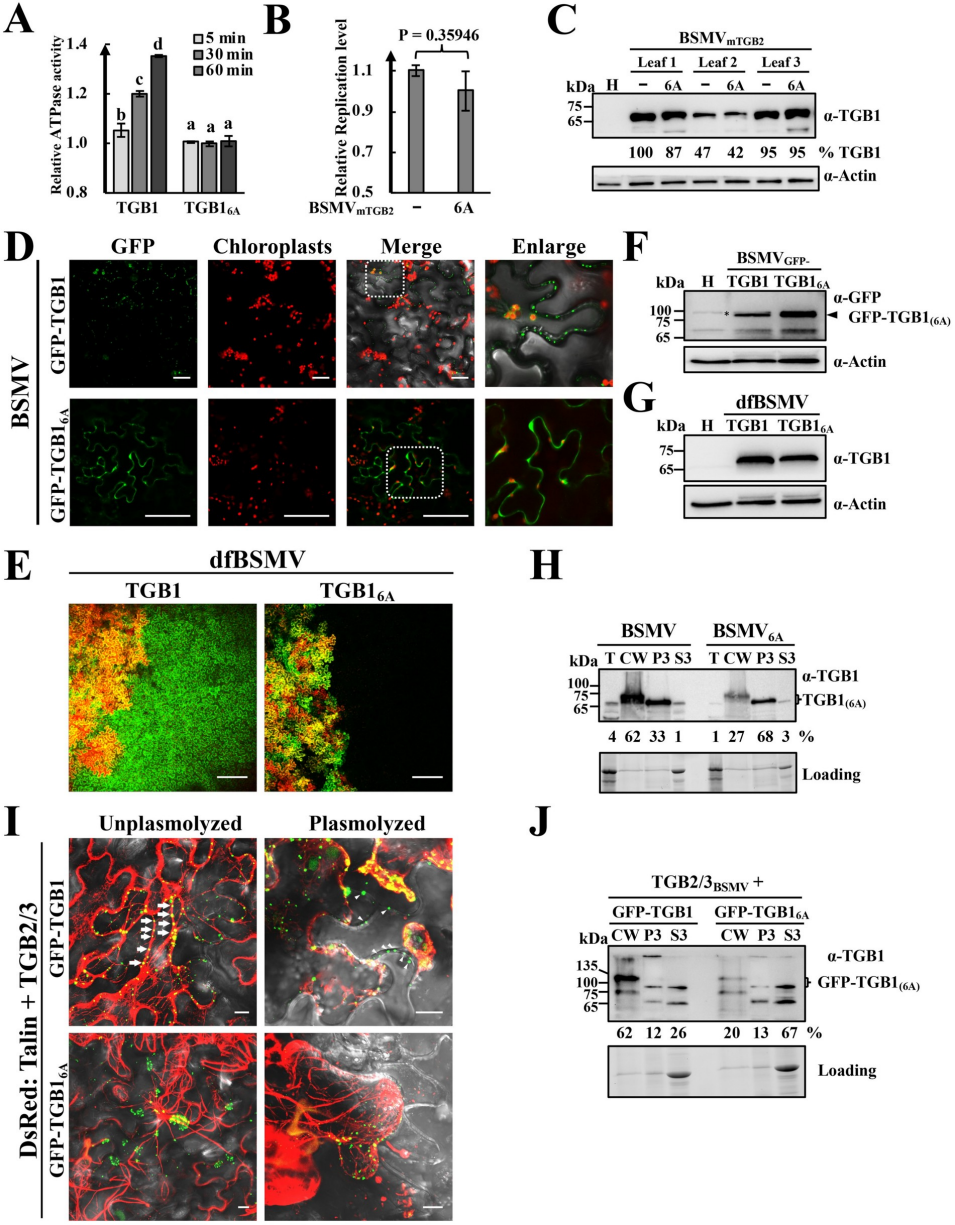
Requirements of BSMV TGB1 ATPase activity for actin filament and PD targeting. **(A)** ATPase activities of TGB1 and TGB1_6A_ proteins. Purified His-tagged TGB1 or TGB1_6A_ proteins were added to ATPase reaction buffers at room temperature, followed by ATP additions to initiate ATP hydrolysis. The relative ATPase activities were analyzed at different time points by power wave XS2. Error bars indicate standard errors of the mean (n = 3). One-way analysis of variance (ANOVA) was used for statistical analysis. Different letters above the bars indicate statistically significant differences (*P* < 0.05) determined by Duncan’s multiple range tests (n = 3). **(B)** RT-qPCR assays of BSMV_mTGB2_ replication in infections with wild-type TGB1 and BSMV_6A-mTGB2_ with 6A mutations. BSMV_mTGB2_ or BSMV_6A-mTGB2_ were infiltrated into the different sites of the same leaf (Half-leaf method). The infiltrated regions were excised at 3 dpi and total RNA extracts were analyzed by RT-qPCR. The results were analyzed by Student’s *t* test. (**C)** Western blot analyses with anti-TGB1 antibodies to detect TGB1 and TGB1_6A_ protein accumulation in BSMV_mTGB2_- or BSMV_6A-mTGB2_-infected *N*. *benthamiana* leaf tissues from different infiltrated regions within the same leaf. Band intensities were quantified with ImageJ software, and the lane 2 adjacent to the healthy leaf control (H) was set to 100%. Protein loading was assessed by actin levels in the protein extracts. **(D)** Confocal microscopy visualization of GFP-TGB1 and GFP-TGB1_6A_ subcellular localization in BSMV infections of *N*. *benthamiana* epidermal cells at 3 dpi. The dotted boxes shown in the merged panels were enlarged. Scale bars, 100 μm. **(E)** Analyses of cell-to-cell movement of BSMV TGB1 6A mutants with the dfBSMV reporter system. Images were captured at 3 dpi. Scale bars, 250 μm. **(F-G**) Western blot analyses of GFP-TGB1 and GFP-TGB1_6A_ (D) or TGB1 and TGB1_6A_ (E) accumulation in the infiltrated leaves shown in panels 6D and 6E. Actin immunoblots represent loading controls. The arrowhead and asterisk in panels F and G indicate the target and unspecific bands. (**H**) Western blot analyses of CW fractions prepared from leaves at 3 dpi after infiltration with *A*. *tumefaciens* harboring wild-type BSMV infectious clones or derivatives containing 6A TGB1 mutations. The loading control indicates the amounts of proteins used for Western blot detections. The band intensities were quantified with ImageJ software. **(I)** Confocal analyses of GFP-TGB1 and GFP-TGB1_6A_ subcellular localization in *N*. *benthamiana* epidermal cells. GFP-TGB1 or GFP-TGB1_6A_ were co-expressed with the TGB2/3 proteins in agroinfiltrated *N*. *benthamiana* leaves as described previously [28]. DsRed:Talin provided an actin marker. Unplasmolyzed leaf tissues were observed by confocal microscopy at 3 dpi or were plasmolyzed with sucrose solutions to evaluate CW associations of TGB1 as described previously [28]. The arrows indicate TGB1 labeling alongside the actin filaments. The arrowheads show retention of TGB1 at the CW after plasmolysis. Scale bars, 10 μm. **(J)** Western blot analysis with anti-TGB1 antibody to detect accumulation of TGB1 and TGB1_6A_ proteins in different cellular fractions. Agroinfiltrated leaf samples shown in panel 6I were harvested at 3 dpi. Loading controls indicate amounts of proteins used for Western blot detections. Band intensities were quantified by with ImageJ software. Molecular weight marker sizes (in kDa) are shown on the left and the antibodies used for detection are shown on the right.

Due to interactions of γb with TGB1 containing the integral ATPase/helicase domain (Fig 5C), we hypothesized that the functional involvement of γb in movement may be associated with TGB1 ATPase/helicase activity. First, we performed an *in vitro* TGB1 ATPase assay (Sigma-Aldrich, Cat. # MAK113) based on binding of released Pi to a malachite green molybdate complex at A_620_. The results showed that BSMV TGB1 possesses ATPase activity *in vitro* as evidenced by significantly increased A_620_ values over time (Fig 6A), as expected from a previous study [22]. We also constructed an ATPase-defective mutant of TGB1 (TGB1_6A_) in which six conserved amino acids (GKS, DE, and Q) in motif I, II, and III were mutated to alanine (Fig 6A). In contrast to wtTGB1, TGB1_6A_ lost ATPase activity as indicated by consistent A_620_ values during analyses at different time points (Fig 6A). We introduced the 6A mutation into a movement deficient construct BSMV_mTGB2_, and then co-infiltrated *N*. *benthamiana* leaves with *A*. *tumefaciens* derivatives harboring plasmids expressing RNAβ_mTGB2_ or RNAβ_6A-mTGB2_ and BSMV RNAα and RNAγ. Due to inactivation of cell-to-cell movement of these BSMV mutants, accumulation of these BSMV derivatives could only be due to virus replication. RT-qPCR analysis of total RNA extracted from the infiltrated leaves revealed that introduction of the 6A mutation into the ATPase domain (BSMV_6A-mTGB2_) failed to affect replication levels compared with those of BSMV_mTGB2_ (Fig 6B). Western blot analyses also confirmed comparable TGB1 accumulation of BSMV_mTGB2_ and BSMV_6A-mTGB2_ (Fig 6C). These results indicate that inactivation of TGB1 ATPase activity does not affect TGB1 accumulation or virus replication in *N*. *benthamiana*.

To investigate functional roles of the ATPase/helicase domain in TGB1 subcellular localization, we introduced the 6A mutation into pCB301-β_GFP-TGB1_ (S1 Fig) to produce TGB1_6A_ and visualized subcellular fluorescence of GFP-TGB1_6A_ mutant and wtGFP-TGB1 in infiltrated tissue at 3 dpi by confocal microscopy. *A*. *tumefaciens* harboring plasmids expressing RNAα and RNAγ were co-infiltrated with *Agrobacterium* containing either pCB301-β_GFP-TGB1_ or pCB301-β_GFP-TGB16A_. As a positive control, confocal analysis of the unmodified BSMVβ_GFP-TGB1_ showed that a considerable amount of the fluorescence appeared as paired spots at the periphery of adjoining cells (Fig 6D, upper right panels), which previous results indicate are PD [21, 41]. Occasional, GFP-TGB1 fluorescent areas were also present around the chloroplasts (Fig 6D, upper right panels). In striking contrast, GFP-TGB1_6A_ fluorescence had a uniform distribution of fluorescence at the cell periphery instead of the paired PD spots (Fig 6D, lower right panel). The 6A mutation was also introduced into the dfBSMV reporter system, and confocal comparisons of fluorescence localization in *N*. *benthamiana* leaves infiltrated with dfBSMV_6A_ and wt dfBSMV showing that GFP fluorescence was completely restricted to the red-colored infiltrated areas containing dfBSMV_6A_ (Fig 6E) reflects abilities of wtTGB1 and TGB1_6A_ to function in subcellular virus spread. Western blot analysis of total protein extracts from leaf regions infiltrated with the BSMVβ_GFP-TGB1_ (3 dpi) or dfBSMV derivatives (3 dpi) revealed similar abundances of TGB1_6A_ and wtTGB1 (Fig 6F and 6G). These results combined with the fact that the TGB1 6A mutation did not affect BSMV replication (Fig 6B and 6C), indicate that the altered subcellular localization of TGB1_6A_ and the significantly reduced cell-to-cell movement of dfBSMV_6A_ are intimately associated with aberrant TGB1 ATPase activity rather than a consequence of changes in the abundance of TGB1.

In additional experiments, we evaluated effects of the 6A mutations on PD targeting of TGB1 by gradient separation. The 6A mutation was introduced into pCB301-β to generate pCB301-β_6A_, and then *A*. *tumefaciens* containing the pCB301-α, pCB301-β_6A_, or pCB301-γ plasmids were co-infiltrated into *N*. *benthamiana* leaves. The CW fractions were isolated from wtBSMV- or BSMV_6A_-infected *N*. *benthamiana* leaves, and Western blot analyses of different fractions revealed that wtTGB1 predominated in the CW-enriched fraction (62% in the fractions shown in Western blot analyses). However, the abundance of TGB1_6A_ decreased substantially in the CW fraction (27% in the Western blot fractions) (Fig 6H), which is consistent with the confocal analyses shown in Fig 6D.

To further elucidate the essential roles of the TGB1 ATPase domain in mediating the PD targeting of TGB1, GFP-fused wtTGB1 or TGB1_6A_ were co-expressed transiently with DsRed:Talin and TGB2/3 to produce a 10:1 ratio of TGB2 to TGB3 in *N*. *benthamiana* epidermal cells [44]. As a positive control, co-expression of GFP-TGB1 and TGB2/3 produced numerous punctate fluorescent bodies at the cell peripheries or in close proximity to actin filaments (Fig 6I, upper left panel). After plasmolysis, fluorescent granules were either retained at the CW or were retracted along with actin filaments (Fig 6I, upper right panel). In contrast, when GFP-TGB1_6A_ was co-expressed with TGB2/3, GFP fluorescence appeared as aggregated forms with greatly reduced actin filament associations compared with the wtTGB1 control (Fig 6I, bottom left panel). Most of the GFP-TGB1_6A_ fluorescent foci retracted from the CW after plasmolysis (Fig 6I, bottom right panel), whereas wtTGB1 was clearly retained at the CW and colocalized with the actin filaments. These results demonstrate that TGB1 ATPase activity is required for efficient TGB1 actin and PD targeting. The CW fraction was also isolated from the agroinfiltrated *N*. *benthamiana* leaves and Western blot analyses of the faction revealed that large amounts of GFP-TGB1 were present in the CW-enriched fraction (62% of the fractions shown in the Western blot analyses). However, enrichment of TGB1_6A_ in the CW fraction significantly decreased to ~20% of the fractions in the Western blot analyses (Fig 6J), and these results are consistent with the confocal analyses shown in Fig 6I. Altogether, these results indicate that ATPase activity is indispensable for TGB1 targeting to actin filaments and PD, and is essential for viral cell-to-cell movement.

### γb promotes TGB1-TGB3 associations for efficient vRNP assembly by enhancing TGB1 ATPase activity

**Fig 7:**
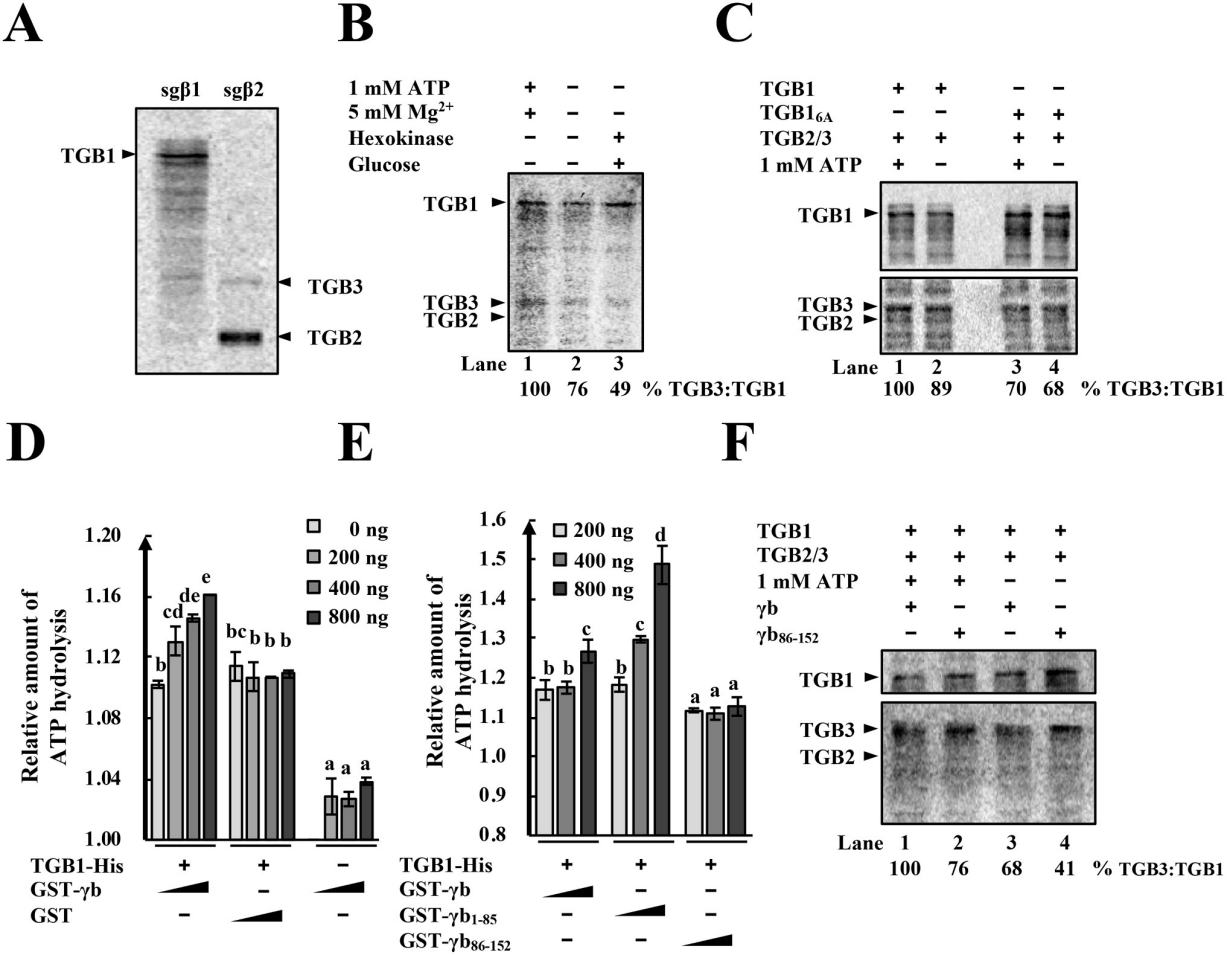
TGB1-TGB3 protein association increases after ATP hydrolysis and γb protein interactions. **(A)** *In vitro* translations of TGB1 from subgenomic RNAβ1 (sgβ1), and TGB2 and TGB3 from sgβ2 RNA in wheat germ extracts. Arrowheads show positions of the target protein bands. Translation products were radiolabeled with ^14^C-Leucine and visualized by phosphor imaging. **(B)** ATP enhancement of TGB1-TGB3 protein binding. The 3xFlag-TGB1, TGB2 and TGB3 proteins were translated *in vitro*, mixed and divided into three treatment groups. Lane 1: TGB1-TGB3 binding in the presence of 1 mM ATP and 5 mM Mg^2+^; Lane 2: No treatment; Lane 3: Depletion of residual ATP by hexokinase and glucose addition to reaction mixes. Co-IP assays were evaluated by recovery from anti-FLAG M2 magnetic beads. Arrowheads show positions of the target protein bands. Band intensities of the TGB1 and TGB3 proteins were quantified by with ImageJ software. The relative ratios of TGB3 to TGB1 are shown below the images and the values of the first lane were set to 100%. **(C)** Effects of TGB1 ATPase disruption on TGB1-TGB3 associations. Flag-tagged TGB1 or the TGB1_6A_ was translated *in vitro* followed by Co-IP assays as described above. The band intensities of TGB1 and TGB3 were measured with ImageJ software and the relative ratios of TGB3 to TGB1 are shown below the images. The value of the lane 1 was set to 100%. Arrowheads indicate the positions of the target protein bands. **(D)** Dose dependent γb protein enhancement of TGB1 ATPase activities. Column 1–4: Increasing amounts of GST-γb protein (0 ng, 200 ng, 400 ng, 800 ng) on 1 μg TGB1-His ATPase activities. Columns 5–8: 1 μg TGB1-His with increasing amounts (0 ng, 200 ng, 400 ng, 800 ng) of GST proteins. Columns 9–12: Increasing amounts (0 ng, 200 ng, 400 ng, 800 ng) of GST-γb protein without TGB1-His. Column 9 is adjusted to 1.00. After incubation for 45 min, ATPase activities of the samples were measured at 620 nm with a microplate reader. Letters above each bar chart indicate statistically significant differences (*P* < 0.05) determined by Duncan’s multiple range test (n = 2). **(E)** Enhancement of TGB1 ATPase activities by TGB1-γb protein interactions. Columns 1–3: TGB1-His (1 μg) with increasing amounts of GST-γb proteins (200 ng, 400 ng, 800 ng). Column 4–6: TGB1-His (1 μg) with increasing amounts (200 ng, 400 ng, 800 ng) of GST-γb_1-85_ proteins. Column 7–9: 1 μg TGB1-His with increasing amounts (200 ng, 400 ng, 800 ng) of GST-γb_86-152_ proteins. Letters above each bar show statistically significant differences (*P* < 0.05) determined by Duncan’s multiple range tests (n = 3). **(F)** γb and γb_86-152_, enhancement of TGB1-TGB3 protein binding in the presence of ATP. *In vitro* translated GST-fused γb or γb_86-152_ proteins were mixed with *in vitro* synthetized TGB1, TGB2 and TGB3 in the presence or absence of ATP, incubated for 90 min, and Co-IP assays performed to evaluate TGB1 and TGB3 protein binding. Band intensities of the TGB1 and TGB3 proteins were quantified with ImageJ software and the relative ratios of TGB3 to TGB1 are shown below the images. Lane 1 is adjusted to 100% for comparisons of the different mixtures. Arrowheads indicate the positions of the target protein bands.

TGB2 and TGB3 are difficult to immunoprecipitate during BSMV infection because of their membrane localization and low-abundance [15], hence biochemical aspects of vRNP movement complex assembly are obscure. To obtain the full-length TGB proteins for studies of vRNP movement complex assembly, three FLAG tag repeats were fused to the TGB1 N-terminus and expressed under a T7 promoter for *in vitro* RNA transcription and translation. DNA fragments corresponding to sgRNAβ2 were also engineered under the T7 promoter to facilitate an ~10:1 ratio of TGB2 and TGB3 [28, 44]. The 3xFlag-TGB1 and TGB2/3 proteins were then translated in a wheat germ cell-free translation system in the presence of radiolabeled ^14^C-Leucine. The results revealed that all three proteins could be detected by phosphorimaging to provide sensitive identification of the translation products and their use in biochemical experiments (Fig 7A).

Previous studies have demonstrated that BSMV TGB1 interacts with TGB3 *in vitro* [13] and that both TGB2 and TGB3 are required for efficient PD targeting of TGB1 [28]. To investigate whether ATPase is required for TGB1-TGB3 associations, *in vitro* translations coupled with Co-IP experiments were conducted in the presence or absence of ATP. We also depleted residual ATP in the samples by adding hexokinase and glucose [45]. We then immunoprecipitated the 3xFlag-TGB1 protein from the *in vitro* translation products with anti-FLAG magnetic beads. The results showed that substantially greater amounts of the TGB3 and TGB2 proteins co-immunoprecipitated with TGB1 in the presence of 1 mM ATP than with lower amounts of ATP (Fig 7B, compare lane 1 with lanes 2 and 3). These results indicate that ATP promotes binding of TGB1 to both TGB3 and TGB2.

To further determine whether TGB1-TGB3 associations are related to the ATPase activity of TGB1, 3xFlag-TGB1_6A_ proteins were translated *in vitro* followed by coimmunoprecipitation with separately translated TGB2/3 proteins. The results indicated that in the positive control, TGB1-TGB3 binding was enhanced by addition of 1 mM ATP when TGB1 ATPase activity was maintained (Fig 7C, compare lane 1 with lane 2). In contrast, when TGB1 ATPase was inactivated by introducing the 6A mutation (TGB1_6A_), addition of ATP to the reaction mix failed to alter TGB3 immunoprecipitation (Fig 7C, compare lane 3 with lane 4), indicating that the TGB1_6A_ mutant failed to respond to the ATP and only retained basal TGB3 binding. These results demonstrate that TGB1 ATPase-mediated ATP hydrolysis promotes TGB1-TGB3 associations.

Because γb interacts with the ATPase/helicase domain of TGB1 (Fig 5C), we examined whether γb regulates TGB1 ATPase activity. TGB1-His, GST-γb, and GST proteins purified from *E*. *coli*. Different amounts of GST-γb or GST proteins were incubated with TGB1-His for 30 min and the ATPase A_620_ values of the group incubated with GST-γb significantly increased during addition of increasing amounts of the GST-γb protein, whereas the A_620_ values of the group incubated with the GST protein failed to increase (Fig 7D). As a control, GST-γb alone was used to test ATPase activity and only background levels of the A_620_ value were present, indicating that the γb protein lacks ATPase activity (Fig 7D). Collectively, these results reveal that γb enhances the ATPase activity of TGB1 in a dose-dependent manner.

Because the 1–85 aa N-terminal γb region is required for γb-TGB1 interactions (Fig 5A and 5B), we also tested whether γb_1-85_ alone could enhance the ATPase activity of TGB1. TGB1-His was incubated with increasing amounts of the GST-γb, GST-γb_1-85_, or GST-γb_86-152_ proteins (Fig 7E). The results indicated that both GST-γb_1-85_ and GST-γb exhibited a strong ability to enhance ATPase activity of TGB1, but in contrast, GST-γb_86-152_ failed to increase the A_620_ values (Fig 7E). These results indicate that interactions of γb with TGB1 are required for enhanced TGB1 ATPase activity and that γb residues 1–85 are sufficient for enhancement.

In addition, *in vitro* translated γb or γb_86-152_ proteins were added to three TGB containing samples and these results indicated that the γb protein can increase TGB1-TGB3-TGB2 complex associations in the presence of ATP, but that γb_86-152_ cannot (Fig 7F, compare lane 1 with lane 2). Moreover, both γb and γb_86-152_ induced stronger associations of TGB1 with TGB3 and TGB2 in the presence of ATP than in its absence (Fig 7F, compare lanes 1 and 2 with lanes 3 and 4). Since trace amounts of ATP are present in *in vitro* translation reactions, we also noted that γb, in contrast to γb_86-152_, can increase associations of TGB1 with TGB3 and TGB2 without exogenous ATP additions (Fig 7F, compare lane 3 with lane 4). Altogether, these results indicate that γb enhances ATPase activity of TGB1 and promotes associations of TGB1 with TGB3 and TGB2 and that these associations lead to efficient assembly of vRNP movement complexes.

### A TGB1 ATPase activity requirement for vRNP movement complex assembly may be characteristic of TGB viruses

**Fig 8:**
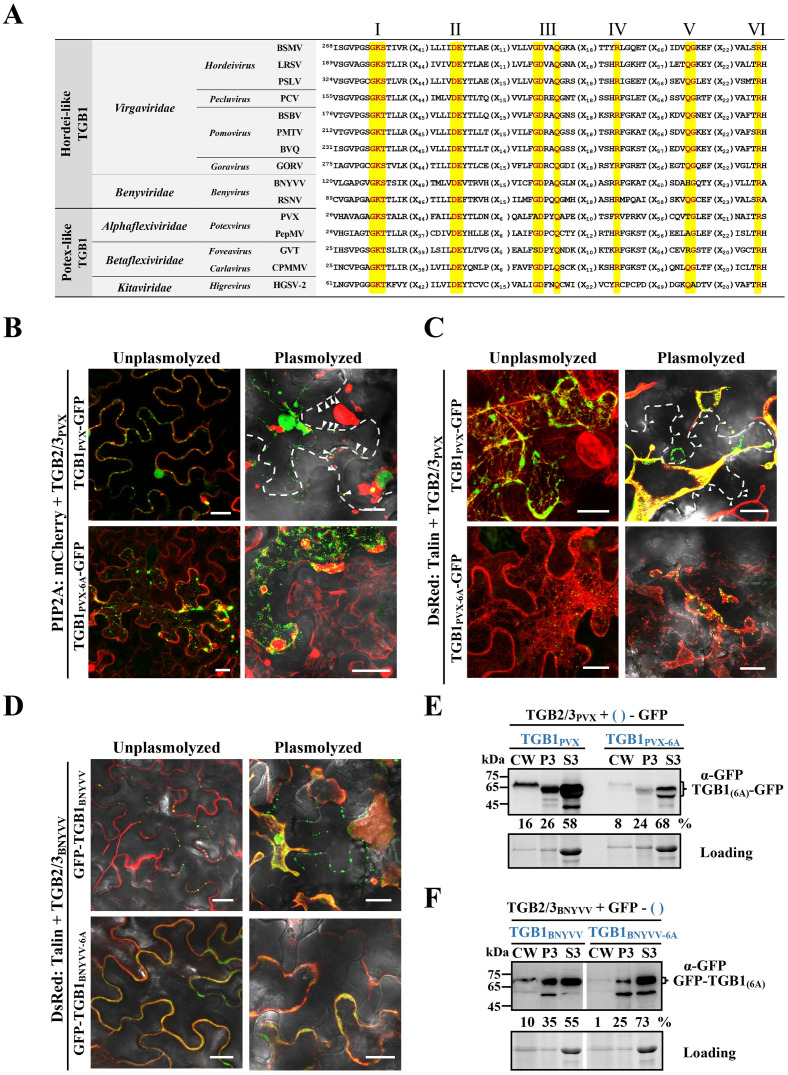
Model for TGB1 ATPase of assembly of vRNP movement complexes of different TGB-encoding viruses. **(A)** Sequence comparison of conserved motifs of ATPase/helicase motif domains I-VI of different TGB-encoding viruses. Conserved residues are shown in red and highlighted in yellow. Amino acid residues in the conserved domains were mutated to alanines to generate the BSMV, PVX, and BNYVV TGB1_6A_ mutants. **(B and C)** Representative confocal images of PVX TGB1-GFP or PVX TGB1_6A_-GFP subcellular localization in the presence of PVX TGB2/3. TGB1_PVX_-GFP and TGB1_PVX-6A_-GFP were co-expressed with TGB2/3_PVX_ in *N*. *benthamiana* epidermal cells. The images were captured at 2 dpi with a confocal microscope. Plasmolyzed cells were infiltrated with 700 mM sucrose. PIP2A:mCherry [39] and DsRed:Talin [28] were used as markers to identify plasma membranes and actin. The white dashed lines and white arrowheads show the CW and TGB1_PVX_-GFP PD retained proteins after plasmolysis. Scale bars, 20 μm. **(D)** Representative confocal images of GFP-TGB1_BNYVV_ or GFP-TGB1_BNYVV-6A_ subcellular localization in the presence of BNYVV TGB2/3. The GFP-TGB1_BNYVV_ or GFP-TGB1_BNYVV-6A_ proteins were co-expressed with TGB2/3_BNYVV_ in *N*. *benthamiana* epidermal cells and observed at 3 dpi. DsRed:Talin was used to label actin filaments. *N*. *benthamiana* tissue was visualized at 2 dpi with a confocal microscope. Scale bars, 20 μm. **(E** and **F)** Fraction of TGB1 proteins enriched from infiltrated *N*. *benthamiana* leaf tissue shown in Panels 8C and 8D. Leaf samples were harvested at 2 or 3 dpi and Western blot analyses were performed with anti-GFP antibodies. Loading controls consist of proteins used for Western blot detection. Band intensities were quantified with ImageJ software. Sizes (in kDa) of molecular weight markers are shown on the left.

Previous studies have shown that PD localizations of PVX TGB1 and BNYVV TGB1 are also dependent on TGB2 and TGB3 [27, 29], and sequence alignments indicate that the TGB1 ATPase/helicase domain of these viruses is highly conserved (Fig 8A). To examine whether an ATPase function is conserved in PVX and BNYVV, six conserved amino acids (GKS, DE, Q) in TGB1 motifs I, II, and III were mutated to alanines (Fig 8A). GFP proteins were fused to the C-terminus of PVX TGB1 or the N-terminus of BNYVV TGB1 as described previously [27, 29]. Then, TGB1_PVX_-GFP or TGB1_PVX-6A_-GFP were transiently co-expressed in *N*. *benthamiana* leaves with TGB2/3_PVX_ and PIP2:mCherry [39] or DsRed:Talin [28]. At 2 dpi, large amounts of PVX TGB1 formed punctate aggregates at the plasma membrane and actin filaments of epidermal cells (Fig 8B and 8C, upper left panel), whereas the 6A mutant was randomly distributed in the cytoplasm and failed to co-localize with actin filaments (Fig 8B and 8C, bottom left panel). After plasmolysis, a number of wtTGB1 punctate bodies of both viruses were retained at the CW and partial TGB1_PVX_-GFP fluorescence retracted to the cytosol, but still displayed strong co-localization with actin filaments (Fig 8B and 8C, upper right panel). However, all of the TGB1_PVX-6A_-GFP fluorescent foci retracted to the cytosol without obvious colocalization with actin filaments (Fig 8B and 8C, bottom right panel).

Similarly, GFP-TGB1_BNYVV_ or the GFP-TGB1_BNYVV-6A_ mutant co-localized with TGB2/3_BNYVV_ and DsRed:Talin [28] in *N*. *benthamiana* epidermal cells at 2 dpi as determined by confocal microscopy. Intriguingly, in contrast to BSMV and PVX, GFP-TGB1_BNYVV_ fluorescent bodies were mainly present at the cell periphery (Fig 8D, upper left panel). In contrast, the GFP-TGB1_BNYVV-6A_ mutant developed diffuse GFP signals along the cell periphery, but relatively few punctate bodies were observed at the CW (Fig 8D, bottom left panel). However, after plasmolysis, the GFP-TGB1_BNYVV_ punctate foci remained at the CW (Fig 8D, upper right panel), but the 6A mutant fluorescence retracted to the cytosol and no detectable fluorescence foci remained at the CW (Fig 8D, bottom right panel). In complementary experiments, CW fractions were isolated from *N*. *benthamiana* leaf tissues shown in Fig 8C and 8D followed by Western blot analyses. These results were consistent with the confocal analyses (Fig 8C and 8D), and showed that both TGB1_PVX_-GFP and GFP-TGB1_BNYVV_ were more abundant in CW-enriched fractions than their corresponding 6A mutants (Fig 8E and 8F). Taken together, these data suggest BSMV, PVX and BNYVV TGB1 ATPase domains function similarly in vRNP complex assembly and localization.

## Discussion

To successfully establish infection, plant viruses have evolved versatile proteins encoded by compact genomes. During the past, numerous studies have focused on multifunctional virus proteins, including *Potyviridae* HC-Pro [46], *Potexvirus* TGB1 [47–50], *Cauliflower mosaic virus* P6 [51], and *Geminiviridae* βC1 [52] and C4 [53, 54]. In addition, *Hordeivirus* γb proteins also carry out well-studied multifunctional activities involved in replication, movement and defense responses [17, 30, 33–36]. In this study, we demonstrated that γb interacts physically with the TGB1 protein (Figs 1 and 3B), which has been previously demonstrated to be a key component of BSMV vRNP movement complexes [13]. Subcellular localization analyses revealed that γb associates with the ER, actin and PD, moves along the ER network (Fig 2 and S1 Video), and is associated with BSMV TGB2 and TGB3 in the ER and actin filaments [41]. By using a BSMV-based BiFC system, we were able to observe γb-TGB1 interactions during virus infection and found that TGB2 and TGB3 modify the subcellular location of the γb-TGB1 complex (Fig 3). By using a Pumilio-based reporter system, we showed that γb also forms mobile punctate granules in association with BSMV genomic (g) RNAs in systemically infected *N*. *benthamiana* epidermal cells (Fig 3D and S2 Video). These data contribute to a model whereby γb participates directly in BSMV intracellular transport by acting as a novel component of vRNP movement complexes.

The movement processes of many plant RNA viruses have been proposed to be coupled to RNA replication. For example, the prototypical P30 MP is recruited by TMV gRNAs to viral replication complexes (VRCs) for movement complex assembly and cell-to-cell movement [1]. Another TGB virus, PVX forms a “caps” structures at PD orifices to couple replication and movement [29]. *Red clover necrotic mosaic virus* (RCNMV) RNA 1 also recruits the 35 kD MP to VRCs [55], and this process is facilitated by a host factor, glyceraldehyde-3-phosphate dehydrogenase (GAPDH) [56]. *Turnip mosaic virus* (TuMV) 6K2 protein also mediates targeting of replication vesicles to PD for cell-to-cell movement [57]. BSMV replication occurs on the chloroplast outer membrane-invaginated spherules [58], but the mechanisms whereby TGBs are targeted to chloroplast replication sites during BSMV infection have not been described previously. Using a BSMV-based BiFC system, we demonstrated that chloroplast localization of the γb-TGB1 complex increased remarkably in the absence of TGB2 and TGB3, suggesting that TGB1 is initially recruited to chloroplasts membranes by VRC-vRNP interactions, a scenario that facilitates coupling of BSMV replication and movement by γb. However, in the presence of TGB2 and TGB3, the TGB1-γb complex localizes primarily at the cell periphery. These events provide a model in which chloroplast localization of replication complexes are transient events, in which replicating vRNAs rapidly interact with TGB1-γb to form complexes that are transported from chloroplast replication sites by TGB2 and TGB3 through the actin cytoskeleton to the PD.

TGB-encoding viruses share several common mechanisms for intra- and intercellular movement. Coordinated interactions of the three TGB proteins are required for viral intra- and intercellular movement [11]. Both hordei-like and potex-like TGB1s share a highly conserved motif in their ATPase/helicase domains (Fig 8A). In PMTV, TGB2 and TGB3 direct formation of intermediate bodies containing TGB1, and although mutations in the ATPase/helicase domain do not alter TGB1 targeting to the intermediate bodies, the mutants impair the PD localization of TGB1 and translocation to adjacent cells [59]. A similar phenomenon has also been observed in BSMV-, BNYVV-, and PVX-infected *N*. *benthamiana* [27, 28, 49], but the underlying mechanisms are unclear. PD localization of these TGB1s strictly depends on cognate TGB2 and TGB3 expression. The assembly of BSMV movement complexes depends on TGB complexes and this assembly is impaired by TGB mutants [24]. Previous studies indicated that BSMV TGB1 has strong ATPase activity *in vitro* [22, 26]. Thus, we initially focused on ATPase activities by mutating TGB1 motif I (GKS) to three alanines (AAA), but, the TGB1_3A_ protein still retained partial ATPase activity as evidenced by ATP hydrolysis upon addition of γb proteins (S8 Fig). This is consistent with previous studies showing that a BSMV TGB1 M1 (K259R) mutant only partially abolished TGB1 PD targeting [28]. We then constructed a TGB1_6A_ mutant that completely inactivates ATPase activity and destroys BSMV cell-to-cell movement (Fig 6). Our cell biology and biochemistry assays demonstrated that TGB1_6A_ lacks the ability to target actin and CW (Fig 6), but does not interfere with TGB3 interactions (Fig 7C), suggesting that the reduced actin and PD associations of TGB1_6A_ are not a consequence of the 6A mutation on TGB1-TGB3 interactions, but instead result from interference with TGB1 ATPase activities.

Unlike animal viruses, plant viruses encode movement proteins that function in increasing the size exclusion limits (SEL) of PD to facilitate cell to cell movement [60]. Among the early studies, TGB movement proteins encoded by PVX and *White clover mosaic virus* (WClMV) were shown to increase PD SEL when co-expressed [61]. Further studies indicated that PVX TGB2 also functions in altering plasmodesmal permeability in *N*. *benthamiana* cells [62]. However, hordei-like TGB1 proteins alone are incapable of independent intracellular trafficking to PD [10], but the extent to which they contribute to PD SEL modifications when expressed with other viral proteins has not been previously determined. Hordeivirus TGB1 proteins have three domains, a disordered extreme N-terminal domain (NTD), an internal domain (ID), and a C-terminal ATPase/helicase domain (HELD) [63]. Previous studies have shown that the NTD and ID domains are responsible for homologous protein-protein interactions and that TGB1 has multiple RNA binding sites [22, 28]. The 6A mutations (Fig 8A) located within the HELD domains do not affect TGB1 multimerization or RNA binding of TGB1, so it is reasonable to presume that the 6A mutations would not substantially affect the ability of TGB1 to “self” interact or bind to viral RNAs. Therefore, other TGB1 activities appear to be crucial for TGB1 PD targeting, and our study demonstrates that the TGB1 ATPase activity has a functional role in mediating associations with the ER/actin network and PD.

In human and mammals, protein complex assembly are highly dependent on ATP, including spliceosomal U1 snRNP assembly [64], maturation of RNA-induced silencing complexes [65], and assembly of human origin recognition complexes (ORC) [66]. In plants, ZAR1, a plant resistance protein, also requires ATP to induce structural remodeling and assembly into a functionally pentameric ZAR1 resistosome [67]. Hordei-like TGB1 proteins also multimerize into high-molecular-weight complexes that disassemble into monomers upon incubation with ATP [63]. Based on these results, we propose that TGB1 conformational changes and assembly of vRNP movement complexes require ATP hydrolysis. Because TGB2 and TGB3 are membrane associated and difficult to purify, there is no direct *in vivo* evidence to demonstrate the association of TGBs. However, we used a wheat germ translation system to synthesize full-length TGB2 and TGB3 proteins in their native ~10:1 ratios to study vRNP movement complex assembly. Using *in vitro* translation and Co-IP assays, we found that both TGB1 and TGB1_6A_ were able to interact with TGB3. But in the present of ATP, TGB1 binding to TGB3 was enhanced substantially, but not the TGB1_6A_ mutant, indicating that hydrolysis of ATP promotes TGB1-TGB3 interactions (Fig 7). Because ER/actin networks are highly dynamic in plants, and actin and PD targeting of TGB1 are strictly dependent on TGB3 [41], we suggest that the basal TGB1-TGB3 interaction is not sufficient to support actin and PD targeting of TGB1, as indicated by the subcellular localization of TGB1_6A_ mutant in Fig 6I. Instead, TGB1 needs to undergo conformational changes mediated by ATP hydrolysis [63] as a prerequisite for associations with TGB3 and stable vRNP movement complexes that can traffic along the ER/actin network. Our study, for the first time, reveals that the optimal assembly of a plant virus vRNP movement complex is an energy consuming event mediated by TGB1 ATPase. In addition to vRNP movement complex assembly, it would be interesting for future investigations to determine whether ATP hydrolysis also has roles in transport of vRNP movement complexes along the ER/actin network.

vRNP movement complex regulation appears to be fine-tuned by multiple host and virus factors during virus movement. Previous studies showed that PMTV TGB2 and TGB3 are reused via endocytic recycling [68], suggesting that vRNP movement complex assembly and disassembly are temporally and spatially regulated during intracellular trafficking. Although, the factors regulating this process remain largely unknown, Hu *et al*. reported that protein kinase CK2 facilitates BSMV cell-to-cell movement by phosphorylating the BSMV TGB1 protein and enhances TGB1-TGB3 interactions [69]. Nucleolar protein fibrillarin (Fib) is another factor that is hijacked by many plant viruses during cell-to-cell and long-distance movement [70–73], BSMV TGB1 also recruits Fib from nucleolar to vRNP movement complexes to promote cell-to-cell movement [21]. This evidence suggests that efficient movement of vRNP complexes requires many interacting regulators. In this study, we demonstrated that the BSMV-encoded γb protein is co-opted by TGB1 for efficient intracellular movement by recruitment to vRNP movement complexes. The γb protein interactions with the TGB1 protein result in enhancement of TGB1 ATPase activity and efficient assembly of vRNP movement complexes. These results extend our knowledge about regulators that are directly involved in viral movement processes. Given that the potex-like CP is also required for cell-to-cell movement [9], it would be interesting to investigate whether PVX CP has γb-like functions similar to those shown here. In addition, although members of the family *Closteroviridae* do not encode a movement-related ATPase protein like those of the TGB-encoding viruses, instead they encode an ATPase-containing Hsp70h protein in their genomes that functions in cell-to-cell movement [74]. Moreover, the *Potyviridae* CI protein is a SF-II helicase and has ATPase activity that is required for PD interactions and viral cell-to-cell movement [75]. These studies suggest that in addition to vRNP movement complexes formed by TGB-encoding viruses, assembly of the movement complexes produced by members of other virus families may also involve energy-coupled processes.

**Fig 9:**
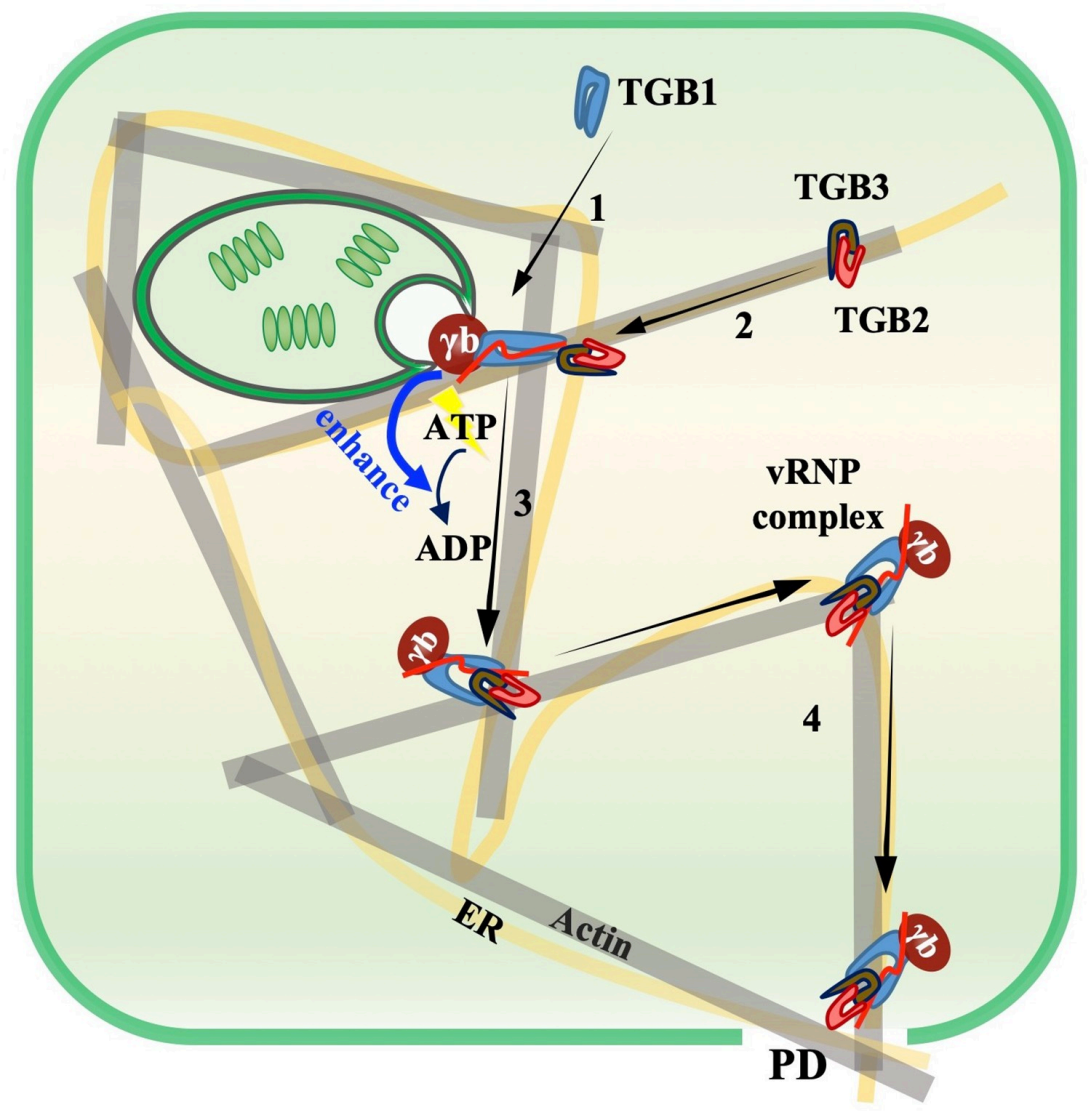
A proposed model for the role of γb in enhancing TGB1 ATPase mediated RNP movement complex assembly. Upon entry into host cells, BSMV replication occurs at chloroplast membrane associated VCRs and sgRNAs are synthesized for translation of TGB proteins. TGB1 moves to the periphery of the chloroplast VRCs, where TGB1 assembles with progeny vRNAs and the γb protein (Step 1). Next, TGB2-TGB3 complexes transit to chloroplasts along ER/actin networks to interact with the TGB1 protein (Step 2). During this process, the TGB1 protein conformation is altered as a consequence of homologous ATPase activities, which are further enhanced by γb protein binding to TGB1 proteins. During ATP hydrolysis, TGB1-TGB3 interactions are postulated to result in formation of functional vRNP movement complexes (Step 3) for intracellular transport along the ER/actin network, targeting to PD, and subsequent cell-to-cell movement (Step 4).

In summary, we propose a model for vRNP movement complex assembly and intracellular movement during BSMV infection (Fig 9). Upon BSMV entry into the cell, VRCs are formed at chloroplasts for synthesis of progeny gRNAs and sgRNAs. After translation, TGB1 is recruited to the periphery of the VRCs by unknown mechanisms, Next, the TGB1 protein binds vRNAs and interacts with γb to form vRNA-TGB1-γb complexes (Fig 9, Step 1). A TGB2-TGB3 complex is formed after co-translation and moves along the ER/actin network and when the complex is adjacent to chloroplasts, TGB3 binds directly to TGB1 (Fig 9, Step 2). During this process, TGB1 conformational changes occur during hydrolysis of ATP, and γb further enhances the ATPase activity of TGB1 to form stable associations of TGB1 with TGB3 and subsequent assembly of functional vRNP movement complexes (Fig 9, Step 3). Finally, the mature vRNP movement complex moves to the PD along the ER/actin network (Fig 9, Step 4). When TGB1_6A_ mutant ATPase activity is destroyed, vRNP movement complex assembly and viral intracellular movement are blocked (Figs 6 and 7, S9 Fig). Our results thus reveal that TGB1 ATPase activity has a pivotal role in assembly of vRNP movement complexes and identify a novel function of the γb protein during BSMV movement. These results enhance our understanding of the multifunctional roles of γb and provide new insight into the assembly and movement of vRNP movement complexes. In summary, the TGB proteins are components of a sophisticated subcellular movement machine that is targeted and regulated by various host and viral factors including the γb protein.

## Conclusion

BSMV γb is a novel positive regulator that participates directly in virus cell-to-cell movement. The formation of vRNP movement complexes are energy-consuming processes that are mediated by TGB1 ATPase, which enhances ATP hydrolysis to boost assembly of vRNP movement complexes. In conclusion, our results enhance our understanding of the versatile roles of γb in regulation of BSMV infection and provide mechanistic insight into the functions of the ATPase activities of the TGB1 protein in the movement of TGB-encoding viruses.

## Materials and methods

### Plant growth conditions

*N*. *benthamiana* plants were grown in a climate-controlled chamber at 23°C with a 15/9 h light/dark photoperiod as described previously [20].

### Plasmid constructions

The BSMV infectious cDNA clones pCB301-α, pCB301-β, and pCB301-γ have been described previously [76]. For Co-IP assays, a 3xFlag tag was engineered downstream of the γb C-terminus by reverse-PCR to generate pCass4-Rz-BSMVγ_γb-3xFlag_. The pCass4-Rz-β_mCP/3xFlag-TGB1_ was described previously [21]. For BiFC assays, the N-terminal half of YFP-fused γb and C-terminal half of YFP-fused TGB1 constructs have been described previously [21, 33]. TGB2, TGB3, and a series of γb truncated fragments were amplified from pCB301-β or pCB301-γ and cloned into the pSPYNE-35S or pSPYCE-35S vectors [77] at the *Bam*HI and *Xho*I sites. The GUS fragment was amplified from the pBl121 binary vector and cloned between the *Bam*HI and *Xho*I sites of the pSPYCE-35S plasmid. For Y2H assays, pGADT7-γb and pGBKT7-γb were described previously [33]. Full-length TGB1, TGB2, and TGB3 were amplified from pCB301-β and cloned into pGADT7-Rec and pGBKT7 (Clontech) at the *Eco*RI and *Bam*HI sites to generate pGADT7-TGB1 and pGBKT7-TGB1. Various TGB1 truncated mutants were constructed using reverse-PCR. For GST pull-down assays, pET30a-TGB1 and pGEX-KG-γb used to express full-length TGB1-His and GST-γb recombinant proteins were described previously [21, 34]. A series of γb truncated fragments were amplified from pCB301-γ and then cloned into the pGEX-KG vector [78] at the *Bam*HI and *Xho*I sites.

For the BSMV-based BiFC system, the C-terminal half of YFP (YFPc, aa 156–240) was amplified from the pSPYCE-35S vector and ligated into the pCB301-β plasmid to generate the pCB301-β_YFPc-TGB1_ construct, and the N-terminal half of YFP (YFPn, aa 1–155) was amplified from the pSPYNE-35S vector and cloned into pCB301-γ_MCS_ [76] by using a Seamless Assembly Cloning Kit (Clone Smarter Technologies Inc., Houston, USA) according to the manufacturer’s instructions. The 12^th^ and 13^th^ amino acid codons of TGB2 were mutated to stop codons to generate the pCB301-β_YFPc-TGB1/mTGB2_ plasmid. The 18^th^ amino acid codon of TGB3 was mutated into a stop codon to generate the pCB301-β_YFPc-TGB1/mTGB3_ plasmid.

For subcellular localization analyses, the BSMV TGB1_6A_ (^258^GKS^260^, ^313^DE^314^, and Q^343^) derivatives, the BNYVV TGB1_6A_ (^128^GKS^130^, ^188^DE^189^, and Q^218^) derivatives and BNYVV TGB1 were cloned into the *Xho*I and *Apa*I sites of the pGDG vector [79]. The PVX TGB1_6A_ derivatives (^34^GKS^36^, ^82^DE^83^, and Q^103^) and PVX TGB1 were cloned into the *Xho*I and *Apa*I sites of the pGDGm vector. PVX TGB2/3 and BNYVV TGB2/3 were amplified from the plasmids pND108 and pCB-BN2 [80, 81] and ligated into the *Xho*I and *Apa*I sites of the pGD vector [79] to yield pGD-TGB2/3_PVX_ and pGD-TGB2/3_BNYVV_.

The primers used for plasmid constructions are listed in S1 Table and sequence analyses were performed to authenticate all plasmids.

### Agroinfiltration

*A*. *tumefaciens* EHA105 strains harboring different expression constructs were infiltrated into *N*. *benthamiana* leaves as described previously [8, 33]. *A*. *tumefaciens* containing each construct was adjusted to OD_600_ = 0.3; and for TBSV P19, the OD_600_ was 0.2. The different cultures were mixed equally (1:1:1) prior to infiltration.

### Coimmunoprecipitation (Co-IP) assays

Co-IP assays were performed as described previously with minor modifications [33]. *A*. *tumefaciens* containing different constructs were agroinfiltrated into *N*. *benthamiana* leaves. Three-gram of fresh leaves were harvested at 3 dpi and ground in liquid nitrogen. The leaf powders were then transferred into a 50 mL tube containing 6 mL of extraction buffer [10% glycerol, 25 mM Tris-HCl, pH 7.5, 1 mM EDTA, 150 mM NaCl, 10 mM DTT, 1 tablet of cOmplete Protease Inhibitor Cocktail (Roche, Cat. # 11697498001), 0.5% (v/v) NP40, 2% (w/v) PVP40 and 0.4% (v/v) Triton X-100 in a final volume of 50 mL]. After 30 min incubation on ice, crude extracts were centrifuged at 30000 *g* for 45 min and the supernatants were incubated with anti-FLAG M2 magnetic beads (Millipore, M8823) for 3 h at 4°C on a rocker platform, followed by washing six times with IP buffer [10% glycerol, 25 mM Tris-HCl, pH 7.5, 1 mM EDTA, 150 mM NaCl, 0.1% (v/v) Tween 20] at 4°C for 10 min per wash. The pelleted beads were boiled for 10 min and analyzed by Western blotting with anti-FLAG (Sigma-Aldrich, Cat. # A2220), anti-γb, or anti-TGB1 antibodies.

### GST pull-down assays

Five μg of proteins fused with GST- or the His-tag were incubated in 600 μL of binding buffer [50 mM Tris-HCl, pH 8.0, 100 mM NaCl, 5 mM DTT, 0.2% glycerol, 0.6% Triton X-100, and 1 tablet of cOmplete Protease Inhibitor Cocktail (Roche, Cat. # 11697498001) in a final volume of 50 mL] for 3 h at 4°C on a rocker platform. Ten μg of RNase A (TIANGEN, P4324) was added to one of the groups. The beads were washed six times with 1 mL wash buffer [50 mM Tris-HCl, pH 8.0, 0.6% Triton X-100, 0.2% glycerol, 600 mM NaCl] at 4°C for 10 min per wash and analyzed by Western blotting with anti-GST or anti-His antibody after a 10 min boiling.

### Yeast two-hybrid (Y2H) assays

Y2H assays were conducted as described previously [33]. Briefly, different γb and TGB1 constructs were transformed into Y187 or Gold yeast strains. Mated yeast cells were plated on SD/-Trp-Leu drop-out media and cultured at 30°C for 3 days. Single colonies were picked and cultured in SD/-Trp-Leu drop-out liquid medium for 24 h at 30°C by shaking at 250 rpm. Then the yeast cells were collected by centrifugation at 3000 *g* for 1 min, washed once with 1 ml of ddH_2_O and resuspended in ddH_2_O. The yeast cells were adjusted to an OD_600_ of 1.0, followed by gradient dilutions with ddH_2_O, and 2 μL was pipetted onto SD/-Trp-Leu plates and SD/-Trp-Leu-His-Ade plates containing 40 **μ**g/mL X-α-Gal (Clontech) media. Yeast cells were cultured at 30°C and photographs were taken after about 4 days.

### BiFC assays

BiFC assays were performed as described previously [77], and at 3 dpi, the samples were observed with a Zeiss LSM710 confocal microscope. YFP signals were excited at 514 nm.

### ATPase activity assays

All the proteins used in this assay were purified from *Escherichia coli* BL21 strain. ATPase activities were analyzed according to the manufacturer’s instruction with an ATPase/GTPase Activity Assay Kit (Sigma-Aldrich, Cat. # MAK113), whose activity can be visualized as a dark green color at (A_620_) formed from reactions with phosphate released during ATPase activity and a malachite green substrate. After 45 min incubation and 30 min termination at room temperature, the samples were measured with a microplate spectrophotometer (BioTek, Power Wave XS2) at 620 nm (A_620_).

### ATP depletion

ATP depletion was performed as previously described [65]. Briefly, the translation products were incubated with 25 mM glucose and 0.5 units/μL hexokinase (Sigma) for 20 min at room temperature to deplete ATP in the reaction mix.

### Total RNA isolation and quantitative reverse transcription PCR (RT-qPCR) analyses

*N*. *benthamiana* total RNA was extracted with TRIzol (Invitrogen, Cat. # 15596018) according to the manufacturer’s instructions. RT-qPCR analysis was performed as described previously with minor modifications [82]. Briefly, 3 μL total RNAs were treated with Recombinant DNase I (RNase free) (TaKaRa) at 37°C for 1 h and then inactivated at 80°C for 10 min. The first cDNA strand was synthesized with M-MLV reverse transcriptase (Promega) and oligo (dT)_18_ primer and incubated at 42°C for 90 min and then inactivated at 75°C for 10 min. Subsequently, the first round cDNAs were quickly cooled on ice and used as templates for RT-qPCR analysis. The DNA fragments corresponding to RNAα were amplified to determine BSMV replication levels. The cycling parameters were: 95°C for 3 min, followed by 40 cycles at 95°C for 10 sec and 53°C for 20 sec. A dissociation curve was generated by increasing temperature from 65 to 95°C to verify primer specificity. The *protein phosphatase 2A* (*PP2A*) gene was used as the internal control [82], and the data were analyzed using Bio-Rad CFX Manager. Primers used for RT-qPCR analysis were listed in S1 Table.

### Statistical analyses

To quantify the relative movement abilities of different BSMV derivatives, the red fluorescent area representing the agroinfiltration region was subtracted from the green fluorescent only area indicative of the BSMV infection, the GFP only resulting areas were then divided by the corresponding red fluorescent areas. At least seven different infiltration regions from different *N*. *benthamiana* leaves were used for calculations and all data were statistically analyzed by using SPSS software (IBM SPSS Statistics, version 23) to evaluate a statistical difference among different groups.

### Confocal laser scanning microscopy

*N*. *benthamiana* leaves were observed with a Leica SP8 or Zeiss LSM710 confocal microscopes. CFP, GFP, YFP, mCherry, and chlorophyll autofluorescence were visualized at 440 nm, 488 nm, 514 nm, 543 nm, and 633 nm, respectively. A line sequential scanning mode was used and detection bands were optimized for each fluorophore group to avoid cross fluorescence effects.

### *In vitro* translation and Co-IP assay

The TnT wheat germ extract system (Promega, Cat. # L4140) together with L-[^14^C(U)]-Leucine (PerkinElmer, Cat. # NEC279E050UC) was used for *in vitro* translation reactions for synthesis of target proteins according to the manufacturer’s instructions. Briefly, DNA fragments corresponding to 3xFlag-TGB1, 3xFlag-TGB1_6A_, and TGB2/3 were amplified using primers listed in S1 Table and purified by electrophoresis through 1% (w/v) agarose gels. Note: To enhance the translation product of TGB1, a Kozak sequence (CCACC) and a spacer (AG) sequence were introduced between the T7 promoter (ATACGACTCACTATAGGG) and the TGB1 encoding sequence. In contrast, the Kozak sequence addition was not used for TGB2/3 translation to maintain the native ~ 10:1 translation ratio. The reaction mix contained 25 μL wheat germ extract, 2 μL reaction buffer, 1 μL T7 RNA polymerase, 1 μL amino acid mixture minus Leucine, 1000 Ci / mmol at 10 mCi/mL ^14^C-Leucine, 1 μL recombinant RNase inhibitor (TaKaRa, Cat. # 2313A, 40 U/μL), 1 μg DNA template, and nuclease-free water in a final volume of 50 μL. *In vitro* translation reaction mixtures were incubated at 30°C for 90 minutes. Note: The 3xFlag-TGB1 and 3xFlag-TGB1_6A_ proteins were translated separately, whereas the TGB2 and TGB3 proteins were co-translated from a single sgRNA template to maintain the ~10 to 1 synthesis ratios resulting from leaky scanning of TGB2. After translation, different proteins were mixed with 1 mM ATP (TaKaRa, Cat. # 4041, 100 mM) and 5 mM magnesium sulfate in IP buffer [20 mM Tris-HCl (pH 7.4), 10% glycerol, 150 mM NaCl, 0.1% (v/v) Triton X-100]. Immunoprecipitations were performed with anti-FLAG M2 magnetic beads (Millipore, M8823) at 4°C for 90 min. The IP products were washed once with IP buffer at 4°C for about 10 min. After boiling in 1xSDS loading buffer for 10 min, the samples were separated on 12.5% or 15% SDS-PAGE gels, electrophoretically transferred to nitrocellulose (Bio-Rad, Cat. # 162–0115), and exposed to a phosphor imaging screen (GE Healthcare) for at least one week. Band signals were measured with a Typhoon 9400 PhosphorImager (GE Healthcare) and quantified by ImageJ software.

### Fractionation analysis of TGB1 proteins

Extraction of different subcellular fractions were performed according to a previous description [83]. Agroinfiltrated *N*. *benthamiana* leaf tissues (4 g) were ground in liquid nitrogen and suspended by vortex mixing in 16 mL of protein extraction buffer [400 mM sucrose, 100 mM Tris-HCl (pH 7.5), 10 mM KCl, 5 mM MgCl_2_, 10% glycerol, 10 mM DTT, 1 tablet of cOmplete Protease Inhibitor Cocktail (Roche, Cat. # 11697498001) to a final volume of 50 mL]. After 20 min incubation on ice, the homogenates were filtered through a nylon mesh into a 50 mL tube to remove the cell walls in the fibrous brie. The crude filtrates were then centrifuged for 10 min at 3000 *g* to produce low-speed pellet fractions (P3). The resulting supernatant was then centrifugated for 30 min at 30000 *g* to separate the S30 supernatant and high-speed pellet (P30) fractions. The P3 and P30 fractions were resuspended by ultrasonic vibrations in 2 mL protein extraction buffer and the samples together with the S30 fraction were analyzed by Western blotting. The CW fractions on the nylon filter were drained carefully with absorbent paper, scraped into a 50 mL tube and resuspended with a pipette in the wash buffer [protein extraction buffer containing 2% (v/v) Triton X-100]. Repeated cycles of centrifugation (10000 *g*) and resuspension were conducted ~ 4 times or until the supernatant became colorless. Then, the pellet was resuspended in 2 mL EBS buffer [4.5% SDS, 9 M urea, 75 mM Tris-HCl (pH 6.8), 10 mM DTT], boiled for 8 min, centrifugated at 20000 *g* for 10 min, and analyzed by Western blotting with anti-TGB1 or anti-GFP antibody. For CW isolation of BNYVV TGB1, the residues were resuspended in the wash buffer without Triton X-100 because repeated Triton X-100 washings removed most of BNYVV TGB1 from the fraction, as has been also observed by earlier investigators [84]. Hence it appears that the BNYVV TGB1 binds less tenaciously to the CW than other TGB1 proteins. After washing twice, the pellets were resuspended in 2 mL EBS buffer containing 2% (v/v) Triton X-100 and analyzed by Western blot with anti-GFP antibody.

### Plasmolysis

Plasmolysis was performed as described previously [28]. Briefly, plasmolysis was induced at 44 hours after agroinfiltration by infiltrating leaves with 700 mM sucrose, followed by cutting and soaking the infiltrated regions in a sucrose solution for 4 ~ 6 hours prior to confocal imaging.

The numerical data used in all figures are included in the S1 Data.

Note: All experiments were performed at least two times and representative results were shown.

## Supporting information

S1 Table Primers used in this study. Primers used for vector construction or other experiments in this work.
(DOCX)

S2 Table Host proteins identified by LC-MS/MS. Host proteins identified by LC-MS/MS after immunoprecipitation of 3xFlag-TGB1 proteins from BSMV_mCP/3xFlag-TGB1_-infected *N*. *benthamiana*.
(DOCX)

S3 Table Host proteins identified by LC-MS/MS. Host proteins identified by LC-MS/MS after immunoprecipitation of γb-3xFlag proteins from BSMV_γb-3xFlag_-infected *N*. *benthamiana*.
(DOCX)

S1 Fig Schematic representation of partial BSMV constructs. BSMV fluorescent reporter constructs used in this study.
(TIF)

S2 Fig Analysis of the specificity of IP assays with 3xFlag-TGB1 bait proteins. *A*. *tumefaciens* containing plasmids expressing RNAα, RNAβ_mCP/3xFlag-TGB1_ or RNAγ were co-infiltrated into *N*. *benthamiana* leaves. After immunoprecipitation with anti-FLAG beads, recovered IP products were analyzed by SDS-PAGE silver staining. The BSMV_mCP_ serves as a negative control.
(TIF)

S3 Fig Western blot with anti-GFP antibodies to confirm protein expression in the infiltrated leaves shown in Fig 1D. The predicted molecular weight of TGB1 is about 57 kDa, but the protein migrates slower than predicted with an apparent size of ~68 kDa; hence the molecular weight of TGB1-YFPc appears to be ~80 kDa, which is comparable to that of the GUS-YFPc negative control. Similar increases of the apparent TGB1 size were also observed in our previous studies [21]. The γb-YFPn protein is ~37 kDa. Uninfiltrated healthy leaves (Healthy) serve as negative controls for Western blot analyses. Sizes (in kDa) of molecular weight markers are shown on the left and antibodies used for detection are indicated on the right, arrowheads indicate the target protein bands.
(TIF)

S4 Fig Analysis of the interactions of γb with TGB2 or TGB3 by Y2H assay. Yeast cells transformed with plasmids indicated on the left were pipetted onto synthetic dextrose dropout media (SD/-Trp-Leu or SD/-Trp-Leu-His-Ade) in a series of 10-fold dilutions. The Y2H combinations containing either empty AD or BD constructs were used as negative controls.
(TIF)

S5 Fig BiFC analyses of γb binding to TGB2 or TGB3. Confocal microscopy of BiFC assays to investigate γb interactions with TGB2 or TGB3 in *N*. *benthamiana* epidermal cells at 3 dpi. Scale bars, 10 μm.
(TIF)

S6 Fig Cell-to-cell movement of BSMV containing wild-type γb or its derivatives (mγb) assayed with the dfBSMV reporter system. Representative confocal images of *N*. *benthamiana* epidermal cells after infiltration with *A*. *tumefaciens* containing different BSMV derivatives at 2 dpi, 3 dpi, and 5 dpi, respectively. The percentage in the upper right of the image indicates the proportion of such case among the observed samples. At least five individual leaf sections were visualized at each time point. Scale bars, 100 μm.
(TIF)

S7 Fig Western blot with anti-Myc and anti-HA antibodies to confirm the protein expression in the infiltrated leaves shown in Fig 5A. The molecular weights of γb_1-24_-YFPn, γb_19-47_-YFPn, γb_60-85_-YFPn, γb_1-85_-YFPn and γb_86-152_-YFPn are about 22 kDa, 23 kDa, 22 kDa, 29 kDa and 27 kDa, respectively. Non-infiltrated healthy leaves (Healthy) serve as a negative control.
(TIF)

S8 Fig *In vitro* ATPase assays to evaluate ATPase activity of TGB1_3A_. The TGB1_3A_-His protein was incubated with increasing amounts of GST-γb and subjected to ATPase assays and A_620_ values were accessed spectrophotometrically. The letters above each bar show statistically significant differences (*P* < 0.05) determined by Duncan’s multiple range test (n = 2).
(TIF)

S9 Fig Confocal microscopy analyses of γb-GFP in leaves infiltrated with *A*. *tumefaciens* containing the BSMV_6A_ mutant. *A*. *tumefaciens* containing plasmids expressing RNAα, RNAβ_6A_, RNAγ_γb-GFP_ or DsRed: Talin were co-infiltrated into *N*. *benthamiana* leaves and the epidermal cells were observed at 3 dpi by confocal microscopy. Scale bar, 20 μm. Chloroplasts are displayed as a false blue color.
(TIF)

S1 Video Mobility of γb-GFP along the *N*. *benthamiana* epidermal cell ER network during BSMV infection. *A*. *tumefaciens* containing plasmids expressing RNAα, RNAβ, RNAγ_γb-GFP_ or mCherry-HDEL [39] were co-infiltrated into *N*. *benthamiana* leaves and time-lapse confocal imaging of the epidermal cells was conducted at about 2 dpi to access fluorescent γb granule movement in the cells.
(MOV)

S2 Video Mobility of γb-mCherry with BSMV genome RNAγ in *N*. *benthamiana* epidermal cells. *A*. *tumefaciens* derivatives containing BSMV_(+)γbPUM_ infectious clones were co-infiltrated into *N*. *benthamiana* leaves as described previously [33]. After 14 days, systemically infected leaves were co-infiltrated with γb-mCherry, CitN-PUMHD3794 and PUMHD3809-CitC, a Pumilio‐based reporter system for imaging vRNAs [43]. At 3 dpi, time-lapse confocal imaging was conducted to document the movement of fluorescent granules in the cells.
(MP4)

S1 Data Excel spreadsheet containing separate sheets with underlying numerical data and statistical analysis for Figs panels 4C, 4E, 5E, 6A, 6B, 7D, 7E and S8. (XLSX)

## References

[1] Heinlein M. Plant virus replication and movement. Virology. 2015; 479: 657–671. 10.1016/j.virol.2015.01.025 25746797

[2] Netherton CL, Wileman T. Virus factories, double membrane vesicles and viroplasm generated in animal cells. Curr Opin Virol. 2011; 1: 381–387. 10.1016/j.coviro.2011.09.008 22440839 PMC7102809

[3] Morozov SY, Dolja VV, Atabekov JG. Probable reassortment of genomic elements among elongated RNA-containing plant viruses. J Mol Evol. 1989; 29: 52–62. 10.1007/BF02106181 2504930 PMC7087513

[4] Petty ITD, Jackson AO. Mutational analysis of *Barley stripe mosaic virus* RNAβ. Virology. 1990; 179: 712–718. 10.1016/0042-6822(90)90138-H 2238467

[5] Beck DL, Guilford PJ, Voot DM, Andersen MT, Forster RLS. Triple gene block proteins of *White clover mosaic potexvirus* are required for transport. Virology. 1991; 183: 695–702. 10.1016/0042-6822(91)90998-Q 1853569

[6] Gilmer D, Bouzoubaa S, Hehn A, Guilley H, Richards K, Jonard G. Efficient cell-to-cell movement of *Beet necrotic yellow vein virus* requires 3' proximal genes located on RNA 2. Virology. 1992; 189: 40–47. 10.1016/0042-6822(92)90679-J 1604825

[7] Herzog E, Hemmer O, Hauser S, Meyer G, Bouzoubaa S, Fritsch C. Identification of genes involved in replication and movement of *Peanut clump virus*. Virology. 1998; 248: 312–322. 10.1006/viro.1998.9287 9721240

[8] Jiang Z, Li Z, Yue N, Zhang K, Li D, Zhang Y. Construction of infectious clones of *Lychnis ringspot virus* and evaluation of its relationship with *Barley stripe mosaic virus* by reassortment of genomic RNA segments. Virus Res. 2018; 243: 106–109. 10.1016/j.virusres.2017.10.012 29054449

[9] Callaway A, Giesman-Cookmeyer D, Gillock ET, Sit TL, Lommel SA. The multifunctional capsid proteins of plant RNA viruses. Annu Rev Phytopathol. 2001; 39: 419–460. 10.1146/annurev.phyto.39.1.419 11701872

[10] Morozov SY, Solovyev AG. Triple gene block: modular design of a multifunctional machine for plant virus movement. J Gen Virol. 2003; 84: 1351–1366. 10.1099/vir.0.18922-0 12771402

[11] Verchot-Lubicz J, Torrance L, Solovyev AG, Morozov SY, Jackson AO, Gilmer D. Varied movement strategies employed by triple gene block-encoding viruses. Mol Plant-Microbe Interact. 2010; 23: 1231–1247. 10.1094/MPMI-04-10-0086 20831404

[12] Solovyev AG, Savenkov EI, Grdzelishvili VZ, Kalinina NO, Morozov SY, Schiemann J, et al Movement of hordeivirus hybrids with exchanges in the triple gene block. Virology. 1999; 253: 278–287. 10.1006/viro.1998.9528 9918886

[13] Lim HS, Bragg JN, Ganesan U, Lawrence DM, Yu J, Isogai M, et al Triple gene block protein interactions involved in movement of *Barley stripe mosaic virus*. J Virol. 2008; 82: 4991–5006. 10.1128/JVI.02586-07 18353960 PMC2346763

[14] Yang Y, Ding B, Baulcombe DC, Verchot J. Cell-to-cell movement of the 25K protein of *Potato virus X* is regulated by three other viral proteins. Mol Plant-Microbe Interact. 2000; 13: 599–605. 10.1094/MPMI.2000.13.6.599 10830259

[15] Jackson AO, Lim HS, Bragg J, Ganesan U, Lee MY. Hordeivirus replication, movement, and pathogenesis. Annu Rev Phytopathol. 2009; 47: 385–422. 10.1146/annurev-phyto-080508-081733 19400645

[16] Navarro JA, Sanchez-Navarro JA, Pallas V. Key checkpoints in the movement of plant viruses through the host. Adv Virus Res. 2019; 104: 1–64. 10.1016/bs.aivir.2019.05.001 31439146

[17] Bragg JN, Jackson AO. The C-terminal region of the *Barley stripe mosaic virus* γb protein participates in homologous interactions and is required for suppression of RNA silencing. Mol Plant Pathol. 2004; 5: 465–481. 10.1111/j.1364-3703.2004.00246.x 20565621

[18] Li Z, Jiang Z, Yang X, Yue N, Wang X, Zhang K, et al Construction of an infectious *Poa semilatent virus* cDNA clone and comparisons of hordeivirus cytopathology and pathogenicity. Phytopathology. 2019; 110: 215–227. 10.1094/PHYTO-06-19-0221-FI 31483225

[19] Yao M, Zhang T, Tian Z, Wang Y, Tao X. Construction of *Agrobacterium*-mediated *Cucumber mosaic virus* infectious cDNA clones and 2b deletion viral vector. Zhongguo Nong Ye Ke Xue. 2011; 44: 4886–4890. 10.1021/jo00394a030

[20] Yuan C, Li C, Yan L, Jackson AO, Liu Z, Han C, et al A high throughput *Barley stripe mosaic virus* vector for virus induced gene silencing in monocots and dicots. PLoS One. 2011; 6: e26468 10.1371/journal.pone.0026468 22031834 PMC3198768

[21] Li Z, Zhang Y, Jiang Z, Jin X, Zhang K, Wang X, et al Hijacking of the nucleolar protein fibrillarin by TGB1 is required for cell-to-cell movement of *Barley stripe mosaic virus*. Mol Plant Pathol. 2018; 19: 1222–1237. 10.1111/mpp.12612 28872759 PMC6638131

[22] Donald RG, Lawrence DM, Jackson AO. The *Barley stripe mosaic virus* 58-kilodalton βb protein is a multifunctional RNA binding protein. J Virol. 1997; 71: 1538–1546. 10.1128/JVI.71.2.1538-1546.1997 8995680 PMC191211

[23] Kalinina NO, Rakitina DA, Yelina NE, Zamyatnin AA Jr., Stroganova TA, Klinov DV, et al RNA-binding properties of the 63 kDa protein encoded by the triple gene block of *Poa semilatent hordeivirus*. J Gen Virol. 2001; 82: 2569–2578. 10.1099/0022-1317-82-10-2569 11562549

[24] Lawrence DM, Jackson AO. Interactions of the TGB1 protein during cell-to-cell movement of *Barley stripe mosaic virus*. J Virol. 2001; 75: 8712–8723. 10.1128/JVI.75.18.8712-8723.2001 11507216 PMC115116

[25] Lawrence DM, Jackson AO. Requirements for cell-to-cell movement of *Barley stripe mosaic virus* in monocot and dicot hosts. Mol Plant Pathol. 2001; 2: 65–75. 10.1046/j.1364-3703.2001.00052.x 20572993

[26] Kalinina NO, Rakitina DV, Solovyev AG, Schiemann J, Morozov SY. RNA helicase activity of the plant virus movement proteins encoded by the first gene of the triple gene block. Virology. 2002; 296: 321–329. 10.1006/viro.2001.1328 12069530

[27] Erhardt M, Morant M, Ritzenthaler C, Stussi-Garaud C, Guilley H, Richards K, et al P42 movement protein of *Beet necrotic yellow vein virus* is targeted by the movement proteins P13 and P15 to punctate bodies associated with plasmodesmata. Mol Plant-Microbe Interact. 2000; 13: 520–528. 10.1094/MPMI.2000.13.5.520 10796018

[28] Lim HS, Bragg JN, Ganesan U, Ruzin S, Schichnes D, Lee MY, et al Subcellular localization of the *Barley stripe mosaic virus* triple gene block proteins. J Virol. 2009; 83: 9432–9448. 10.1128/JVI.00739-09 19570874 PMC2738231

[29] Tilsner J, Linnik O, Louveaux M, Roberts IM, Chapman SN, Oparka KJ. Replication and trafficking of a plant virus are coupled at the entrances of plasmodesmata. J Cell Biol. 2013; 201: 981–995. 10.1083/jcb.201304003 23798728 PMC3691464

[30] Bragg JN, Lawrence DM, Jackson AO. The N-terminal 85 amino acids of the *Barley stripe mosaic virus* γb pathogenesis protein contain three zinc-binding motifs. J Virol. 2004; 78: 7379–7391. 10.1128/JVI.78.14.7379-7391.2004 15220411 PMC434125

[31] Edwards MC. Mapping of the seed transmission determinants of *Barley stripe mosaic virus*. Mol Plant-Microbe Interact. 1995; 8: 906–915. 10.1094/MPMI-8-0906 8664501

[32] Yelina NE, Savenkov EI, Solovyev AG, Morozov SY, Valkonen JP. Long-distance movement, virulence, and RNA silencing suppression controlled by a single protein in hordei- and potyviruses: complementary functions between virus families. J Virol. 2002; 76: 12981–12991. 10.1128/JVI.76.24.12981-12991.2002 12438624 PMC136670

[33] Zhang K, Zhang Y, Yang M, Liu S, Li Z, Wang X, et al The *Barley stripe mosaic virus* γb protein promotes chloroplast-targeted replication by enhancing unwinding of RNA duplexes. PLoS Pathog. 2017; 13: e1006319 10.1371/journal.ppat.1006319 28388677 PMC5397070

[34] Yang M, Zhang Y, Xie X, Yue N, Li J, Wang XB, et al *Barley stripe mosaic virus* γb protein subverts autophagy to promote viral infection by disrupting the ATG7-ATG8 interaction. Plant Cell. 2018; 30: 1582–1595. 10.1105/tpc.18.00122 29848767 PMC6096602

[35] Yang M, Li Z, Zhang K, Zhang X, Zhang Y, Wang X, et al *Barley stripe mosaic virus* γb interacts with glycolate oxidase and inhibits peroxisomal ROS production to facilitate virus infection. Mol Plant. 2018; 11: 338–341. 10.1016/j.molp.2017.10.007 29066357

[36] Zhang X, Dong K, Xu K, Zhang K, Jin X, Yang M, et al *Barley stripe mosaic virus* infection requires PKA-mediated phosphorylation of γb for suppression of both RNA silencing and the host cell death response. New Phytol. 2018; 218: 1570–1585. 10.1111/nph.15065 29453938

[37] Petty ITD, French R, Jones RW, Jackson AO. Identification of *Barley stripe mosaic virus* genes involved in viral RNA replication and systemic movement. EMBO J. 1990; 9: 3453–3457. 10.1002/j.1460-2075.1990.tb07553.x 2209552 PMC552093

[38] Donald RGK, Jackson AO. RNA-binding activities of *Barley stripe mosaic virus* γb fusion proteins. J Gen Virol. 1996; 77: 879–888. 10.1099/0022-1317-77-5-879 8609484

[39] Nelson BK, Cai X, Nebenfuhr A. A multicolored set of *in vivo* organelle markers for co-localization studies in *Arabidopsis* and other plants. Plant J. 2007; 51: 1126–1136. 10.1111/j.1365-313X.2007.03212.x 17666025

[40] Sparkes IA, Frigerio L, Tolley N, Hawes C. The plant endoplasmic reticulum: a cell-wide web. Biochem J. 2009; 423: 145–155. 10.1042/BJ20091113 19772494

[41] Lim HS, Lee MY, Moon JS, Moon JK, Yu YM, Cho IS, et al Actin cytoskeleton and golgi involvement in *Barley stripe mosaic virus* movement and cell wall localization of triple gene block proteins. Plant Pathol J. 2013; 29: 17–30. 10.5423/PPJ.OA.09.2012.0144 25288925 PMC4174794

[42] Wright KM, Wood NT, Roberts AG, Chapman S, Boevink P, Mackenzie KM, et al Targeting of TMV movement protein to plasmodesmata requires the actin/ER network: evidence from FRAP. Traffic. 2007; 8: 21–31. 10.1111/j.1600-0854.2006.00510.x 17132144

[43] Tilsner J, Linnik O, Christensen NM, Bell K, Roberts IM, Lacomme C, et al Live-cell imaging of viral RNA genomes using a Pumilio-based reporter. Plant J. 2009; 57: 758–770. 10.1111/j.1365-313X.2008.03720.x 18980643

[44] Zhou H, Jackson AO. Expression of the *Barley stripe mosaic virus* RNAβ "triple gene block". Virology. 1996; 216: 367–379. 10.1006/viro.1996.0072 8607266

[45] Kawamata T, Seitz H, Tomari Y. Structural determinants of miRNAs for RISC loading and slicer-independent unwinding. Nat Struct Mol Biol. 2009; 16: 953–960. 10.1038/nsmb.1630 19684602

[46] Valli AA, Gallo A, Rodamilans B, Lopez-Moya JJ, Garcia JA. The HCPro from the potyviridae family: an enviable multitasking helper component that every virus would like to have. Mol Plant Pathol. 2018; 19: 744–763. 10.1111/mpp.12553 28371183 PMC6638112

[47] Angell SM, Davies C, Baulcombe DC. Cell-to-cell movement of *Potato virus X* is associated with a change in the size-exclusion limit of plasmodesmata in trichome cells of *Nicotiana clevelandii*. Virology. 1996; 216: 197–201. 10.1006/viro.1996.0046 8614986

[48] Atabekov JG, Rodionova NP, Karpova OV, Kozlovsky SV, Poljakov VY. The movement protein-triggered in situ conversion of *Potato virus X* virion RNA from a nontranslatable into a translatable form. Virology. 2000; 271: 259–263. 10.1006/viro.2000.0319 10860880

[49] Tilsner J, Linnik O, Wright KM, Bell K, Roberts AG, Lacomme C, et al The TGB1 movement protein of *Potato virus X* reorganizes actin and endomembranes into the X-body, a viral replication factory. Plant Physiol. 2012; 158: 1359–1370. 10.1104/pp.111.189605 22253256 PMC3291258

[50] Bayne EH, Rakitina DV, Morozov SY, Baulcombe DC. Cell-to-cell movement of *Potato Potexvirus X* is dependent on suppression of RNA silencing. Plant Journal. 2005; 44: 471–482. 10.1111/j.1365-313X.2005.02539.x 16236156

[51] Leisner SM, Schoelz JE. Joining the crowd: integrating plant virus proteins into the larger world of pathogen effectors. Annu Rev Phytopathol. 2018; 56: 89–110. 10.1146/annurev-phyto-080417-050151 29852091

[52] Li F, Yang X, Bisaro DM, Zhou X. The betaC1 protein of Geminivirus-betasatellite complexes: a target and repressor of host defenses. Mol Plant. 2018; 11: 1424–1426. 10.1016/j.molp.2018.10.007 30404041

[53] Fondong VN. The ever-expanding role of C4/AC4 in geminivirus infection: punching above its weight? Mol Plant. 2019; 12: 145–147. 10.1016/j.molp.2018.12.006 30578853

[54] Zeng R, Liu X, Yang C, Lai J. Geminivirus C4: interplaying with receptor-like kinases. Trends Plant Sci. 2018; 23: 1044–1046. 10.1016/j.tplants.2018.09.003 30279072

[55] Kaido M, Tsuno Y, Mise K, Okuno T. Endoplasmic reticulum targeting of the *Red clover necrotic mosaic virus* movement protein is associated with the replication of viral RNA1 but not that of RNA2. Virology. 2009; 395: 232–242. 10.1016/j.virol.2009.09.022 19819513

[56] Kaido M, Abe K, Mine A, Hyodo K, Taniguchi T, Taniguchi H, et al GAPDH—a recruits a plant virus movement protein to cortical virus replication complexes to facilitate viral cell-to-cell movement. PLoS Pathog. 2014; 10: e1004505 10.1371/journal.ppat.1004505 25411849 PMC4239097

[57] Movahed N, Patarroyo C, Sun J, Vali H, Laliberte JF, Zheng H. Cylindrical inclusion protein of *Turnip mosaic virus* serves as a docking point for the intercellular movement of viral replication vesicles. Plant Physiol. 2017; 175: 1732–1744. 10.1104/pp.17.01484 29089395 PMC5717746

[58] Jin X, Jiang Z, Zhang K, Wang P, Cao X, Yue N, et al Three-dimensional analysis of chloroplast structures associated with virus infection. Plant Physiol. 2018; 176: 282–294. 10.1104/pp.17.00871 28821590 PMC5761806

[59] Zamyatnin AA Jr., Solovyev AG, Savenkov EI, Germundsson A, Sandgren M, Valkonen JP, et al Transient coexpression of individual genes encoded by the triple gene block of *Potato mop-top virus* reveals requirements for TGBp1 trafficking. Mol Plant-Microbe Interact. 2004; 17: 921–930. 10.1094/MPMI.2004.17.8.921 15305613

[60] Ueki S, Citovsky V. To Gate, or not to gate: regulatory mechanisms for intercellular protein transport and virus movement in plants. Molecular Plant. 2011; 4: 782–793. 10.1093/mp/ssr060 21746703 PMC3183397

[61] Lough TJ, Shash K, Xoconostle-Cazares B, Hofstra KR, Beck DL, Balmori E, et al Molecular dissection of the mechanism by which potexvirus triple gene block proteins mediate cell-to-cell transport of infectious RNA. Mol Plant-Microbe Interact. 1998; 11: 801–814. 10.1094/MPMI.1998.11.8.801

[62] Tamai A, Meshi T. Cell-to-cell movement of *Potato virus X*: the role of p12 and p8 encoded by the second and third open reading frames of the triple gene block. Mol Plant-Microbe Interact. 2001; 14: 1158–1167. 10.1094/MPMI.2001.14.10.1158 11605955

[63] Makarov VV, Makarova SS, Makhotenko AV, Obraztsova EA, Kalinina NO. *In vitro* properties of hordeivirus TGB1 protein forming ribonucleoprotein complexes. J Gen Virol. 2015; 96: 3422–3431. 10.1099/jgv.0.000252 26276346

[64] Meister G, Buhler D, Pillai R, Lottspeich F, Fischer U. A multiprotein complex mediates the ATP-dependent assembly of spliceosomal U snRNPs. Nat Cell Biol. 2001; 3: 945–949. 10.1038/ncb1101-945 11715014

[65] Yoda M, Kawamata T, Paroo Z, Ye X, Iwasaki S, Liu Q, et al ATP-dependent human RISC assembly pathways. Nat Struct Mol Biol. 2010; 17: 17–23. 10.1038/nsmb.1733 19966796 PMC2915567

[66] Siddiqui K, Stillman B. ATP-dependent assembly of the human origin recognition complex. J Biol Chem. 2007; 282: 32370–32383. 10.1074/jbc.M705905200 17716973

[67] Wang J, Hu M, Wang J, Qi J, Han Z, Wang G, et al Reconstitution and structure of a plant NLR resistosome conferring immunity. Science. 2019; 364: 44 10.1126/science.aav5870 30948527

[68] Haupt S, Cowan GH, Ziegler A, Roberts AG, Oparka KJ, Torrance L. Two plant-viral movement proteins traffic in the endocytic recycling pathway. Plant Cell. 2005; 17: 164–181. 10.1105/tpc.104.027821 15608333 PMC544497

[69] Hu Y, Li ZG, Yuan C, Jin XJ, Yan LJ, Zhao XF, et al Phosphorylation of TGB1 by protein kinase CK2 promotes *Barley stripe mosaic virus* movement in monocots and dicots. J Exp Bot. 2015; 66: 4733–4747. 10.1093/jxb/erv237 25998907 PMC4507770

[70] Chang CH, Hsu FC, Lee SC, Lo YS, Wang JD, Shaw J, et al The nucleolar fibrillarin protein is required for helper virus-independent long-distance trafficking of a subviral satellite RNA in plants. Plant Cell. 2016; 28: 2586–2602. 10.1105/tpc.16.00071 27702772 PMC5134973

[71] Semashko MA, Gonzalez I, Shaw J, Leonova OG, Popenko VI, Taliansky ME, et al The extreme N-terminal domain of a hordeivirus TGB1 movement protein mediates its localization to the nucleolus and interaction with fibrillarin. Biochimie. 2012; 94: 1180–1188. 10.1016/j.biochi.2012.02.005 22349738

[72] Wang XH, Zhang YJ, Xu J, Shi LD, Fan HY, Han CG, et al The R-rich motif of *Beet black scorch virus* P7a movement protein is important for the nuclear localization, nucleolar targeting and viral infectivity. Virus Res. 2012; 167: 207–218. 10.1016/j.virusres.2012.05.001 22626884 PMC7172424

[73] Kim SH, MacFarlane S, Kalinina NO, Rakitina DV, Ryabov EV, Gillespie T, et al Interaction of a plant virus-encoded protein with the major nucleolar protein fibrillarin is required for systemic virus infection. Proc Natl Acad Sci U S A. 2007; 104: 11115–11120. 10.1073/pnas.0704632104 17576925 PMC1904140

[74] Peremyslov VV, Hagiwara Y, Dolja VV. HSP70 homolog functions in cell-to-cell movement of a plant virus. Proc Natl Acad Sci U S A. 1999; 96: 14771–14776. 10.1073/pnas.96.26.14771 10611288 PMC24723

[75] Carrington JC, Jensen PE, Schaad MC. Genetic evidence for an essential role for potyvirus CI protein in cell-to-cell movement. Plant J. 1998; 14: 393–400. 10.1046/j.1365-313X.1998.00120.x 9670556

[76] Hu J, Li S, Li Z, Li H, Song W, Zhao H, et al A *Barley stripe mosaic virus*-based guide RNA delivery system for targeted mutagenesis in wheat and maize. Mol Plant Pathol. 2019; 20: 1463–1474. 10.1111/mpp.12849 31273916 PMC6792137

[77] Walter M, Chaban C, Schutze K, Batistic O, Weckermann K, Nake C, et al Visualization of protein interactions in living plant cells using bimolecular fluorescence complementation. Plant J. 2004; 40: 428–438. 10.1111/j.1365-313X.2004.02219.x 15469500

[78] Guan KL, Dixon JE. Eukaryotic proteins expressed in *Escherichia coli*: an improved thrombin cleavage and purification procedure of fusion proteins with glutathione S-transferase. Anal Biochem. 1991; 192: 262–267. 10.1016/0003-2697(91)90534-Z 1852137

[79] Goodin MM, Dietzgen RG, Schichnes D, Ruzin S, Jackson AO. pGD vectors: versatile tools for the expression of green and red fluorescent protein fusions in agroinfiltrated plant leaves. Plant J. 2002; 31: 375–383. 10.1046/j.1365-313X.2002.01360.x 12164816

[80] Zhang LD, Wang ZH, Wang XB, Li DW, Han CG, Zhai YF, et al Two virus-encoded RNA silencing suppressors, P14 of *Beet necrotic yellow vein virus* and S6 of *Rice black streak dwarf virus*. Chinese Sci Bull. 2005; 50: 305–310. 10.1007/BF02897570

[81] Jiang N, Zhang C, Liu JY, Guo ZH, Zhang ZY, Han CG, et al Development of *Beet necrotic yellow vein virus*-based vectors for multiple-gene expression and guide RNA delivery in plant genome editing. Plant Biotechnol J. 2019; 17: 1302–1315. 10.1111/pbi.13055 30565826 PMC6576094

[82] Liu D, Shi L, Han C, Yu J, Li D, Zhang Y. Validation of reference genes for gene expression studies in virus-infected *Nicotiana benthamiana* using quantitative real-time PCR. PLoS One. 2012; 7: e46451 10.1371/journal.pone.0046451 23029521 PMC3460881

[83] Donald RGK, Zhou H, Jackson AO. Serological analysis of *Barley stripe mosaic virus*-encoded proteins in infected barley. Virology. 1993; 195: 659–668. 10.1006/viro.1993.1417 8337837

[84] Niesbach-Klosgen U, Guilley H, Jonard G, Richards K. Immunodetection *in vivo* of *Beet necrotic yellow vein virus*-encoded proteins. Virology. 1990; 178: 52–61. 10.1016/0042-6822(90)90378-5 2202150

