# Supplementary material for: The Barley stripe mosaic virus γb protein promotes viral cell-to-cell movement by enhancing ATPase-mediated assembly of ribonucleoprotein movement complexes
Source: PLoS Pathog. 2020 Jul 30;16(7):e1008709. doi: 10.1371/journal.ppat.1008709 (PMC7419011; doi:10.1371/journal.ppat.1008709)
Supplement: S1 Table — Primers used for vector construction or other experiments in this work. (DOCX) [file ppat.1008709.s001.docx]

**S1 Table.** Primers used in this study.

| **Name** | **Primer sequence (5'-3')** | **Purpose** | **Experiment** | **Underline description** |  |
| --- | --- | --- | --- | --- | --- |
| γb-3xFlag F | AAGATCATGATATCGATTACAAGGATGACGATGACAAGCTTTAAAAAAAAAAAAAAATGTTTGATC | pCass4-Rz-γ_γb-3xFlag_ | co-IP | 3xFlag sequence |  |
| γb-3xFlag R | TATAATCACCGTCATGGTCTTTGTAGTCCATGCCACCTCCCTTAGAAACGGAAGAAGAATCATCA |  |  |  |  |
|  | | | | |  |
| TGB3 *Bam*HI F | CGGGATCCATGGCAATGCCTCATC | pSPYNE-TGB3 pSPYCE-TGB3 | BiFC | *Bam*HI restriction enzyme site |  |
| TGB3 *Xho*I R | CCGCTCGAGCCTTTTTGAAGAAAG |  |  | *Xho*I restriction enzyme site |  |
| TGB2 *Bam*HI F | CGGGATCCATGAAGACCACAGTTGGTTCAAGG | pSPYNE-TGB2 pSPYCE-TGB2 |  | *Bam*HI restriction enzyme site |  |
| TGB2 *Xho*I R | CGGGATCCGCCAATATCGCATAGTAATGATC |  |  | *Xho*I restriction enzyme site |  |
| γb *Bam*HI F | CGGGATCCATGATGGCTACTTTCTCTTGTG | pSPYNE-γb_1-24_ |  | *Bam*HI restriction enzyme site |  |
| γb24 *Xho*I R | CCGCTCGAGCTCACATCTCTTACCACAGTAAG |  |  | *Xho*I restriction enzyme site |  |
| γb19 *Bam*HI F | CGGGATCCATGTGTGGTAAGAGATGTGAGCG | pSPYNE-γb_19-47_ |  | *Bam*HI restriction enzyme site |  |
| γb47 *Xho*I R | CCGCTCGAGCGGTTCCAATAGATACTTCTTG |  |  | *Xho*I restriction enzyme site |  |
| γb60 *Bam*HI F | CGGGATCCATGTGTGGAATGCCATGCTCCATTGC | pSPYNE-γb_60-85_ |  | *Bam*HI restriction enzyme site |  |
| γb85 *Xho*I R | CCGCTCGAGATGCTTTTGGCCACAGAACC |  |  | *Xho*I restriction enzyme site |  |
| γb *Bam*HI F | CGGGATCCATGATGGCTACTTTCTCTTGTG | pSPYNE-γb_1-85_ |  | *Bam*HI restriction enzyme site |  |
| γb85 *Xho*I R | CCGCTCGAGATGCTTTTGGCCACAGAACC |  |  | *Xho*I restriction enzyme site |  |
| γb86 *Bam*HI F | CGGGATCCATGGCGGATCTGTATGATTCACTTCTG | pSPYNE-γb_86-152_ |  | *Bam*HI restriction enzyme site |  |
| γb *Xho*I R | CCGCTCGAGCTTAGAAACGGAAGAAGAATCATC |  |  | *Xho*I restriction enzyme site |  |
| GUS *Bam*HI F | CGGGATCCATGTTACGTCCTGTAGAAACCCCAAC | pSPYCE-GUS |  | *Bam*HI restriction enzyme site |  |
| GUS *Xho*I R | CCGCTCGAGTTGTTTGCCTCCCTGCTGCGG |  |  | *Xho*I restriction enzyme site |  |
|  | | | | | |
| reversePCR-AD R | CATATGAGCGTAATCTGGTACGTCG | pGADT7-TGB1_𝚫N16_  pGADT7-TGB1_𝚫N55_  pGADT7-TGB1_𝚫N74_  pGADT7-TGB1_𝚫N105_  pGADT7-TGB1_𝚫C22_  pGADT7-TGB1_𝚫C113_  pGADT7-TGB1_𝚫C186_ | Yeast two hybrid |  |  |
| reversePCR TGB1_N16_ F | TCAGTGAAAGGTGTTTTTGAAAACTCG |  |  |  |  |
| reversePCR TGB1_N55_ F | ACTCCATTGTCCGTTGACAACG |  |  |  |  |
| reversePCR TGB1_N74_ F | GTTCCTGGTCCTAAGTTGGCAAC |  |  |  |  |
| reversePCR TGB1_N105_ F | GCTCAACCGAGTAGGCCCAATG |  |  |  |  |
| reversePCR TGB1_C186_ R | TTATTGTAACAACAGGATTTCCGCGCTCTC |  |  |  |  |
| reversePCR TGB1_C113_ R | TTAGCCATCGTAATCAGTAATGATCACTGTGTCC |  |  |  |  |
| reversePCR TGB1_C22_ R | TTACTTAGAGCTCAAGTCAATATCACCTGT |  |  |  |  |
| reversePCR-AD F | GGATCCATCGAGCTCGAGCTGC |  |  |  |  |
| TGB1-AD/BK *Eco*RI F | CGGAATTCATGGACATGACGAAAACTGTTGAG | pGADT7-TGB1 pGBKT7-TGB1 |  | *Eco*RI restriction enzyme site |  |
| TGB1-AD/BK *Eco*RI R | CGGGATCCTTATTTGGCCTTGAACCAACTGTG |  |  | *Eco*RI restriction enzyme site |  |
| TGB2-AD/BK *Eco*RI F | CGGAATTCATGAAGACCACAGTTGGTTCA | pGADT7-TGB2 pGBKT7-TGB2 |  | *Eco*RI restriction enzyme site |  |
| TGB2-AD/BK *Bam*HI R | CGGGATCCCTAGCCAATATCGCATAGTAA |  |  | *Bam*HI restriction enzyme site |  |
| TGB3-AD/BK *Eco*RI F | CGGAATTCATGGCAATGCCTCATCCC | pGADT7-TGB3 pGBKT7-TGB3 |  | *Eco*RI restriction enzyme site |  |
| TGB3-AD/BK *Bam*HI R | CGGGATCCTTACCTTTTTGAAGAAAGTAAGAG |  |  | *Bam*HI restriction enzyme site |  |
|  | | | | | |
| γb *Bam*HI F | CGGGATCCATGATGGCTACTTTCTCTTG | pGEX-KG-γb_1-85_ | GST pull-down | *Bam*HI restriction enzyme site |  |
| γb_1-85_ *Xho*I R | CCGCTCGAGTTAATGCTTTTGGCCACAGAACC |  |  | *Xho*I restriction enzyme site |  |
| γb_86-152_ *Bam*HI F | CGGGATCCATGGCGGATCTGTATGATTCACTTC | pGEX-KG-γb_86-152_ |  | *Bam*HI restriction enzyme site |  |
| γb *Xho*I R | CCGCTCGAGTTACTTAGAAACGGAAGAAG |  |  | *Xho*I restriction enzyme site |  |
| γb_1-19_ *Bam*HI F | CGGGATCCATGTGTGGTAAGAGATGTGAGCG | pGEX-KG-γb_1-19_ |  | *Bam*HI restriction enzyme site |  |
| γb_1-47_ *Xho*I R | CCGCTCGAGTTACGGTTCCAATAGATACTTCTTG |  |  | *Xho*I restriction enzyme site |  |
| γb_1-60_ *Bam*HI F | CGGGATCCATGTGTGGAATGCCATGCTCCATTGC | pGEX-KG-γb_60-85_ |  | *Bam*HI restriction enzyme site |  |
| γb_1-85_ *Xho*I R | CCGCTCGAGTTAATGCTTTTGGCCACAGAACC |  |  | *Xho*I restriction enzyme site |  |
| γb *Bam*HI F | CGGGATCCATGATGGCTACTTTCTCTTG | pGEX-KG-γb_1-24_ |  | *Bam*HI restriction enzyme site |  |
| γb_1-24_ *Xho*I R | CCGCTCGAGCTCACATCTCTTACCACAGTAAG |  |  | *Xho*I restriction enzyme site |  |
|  | | | | | |
| cYFP *Nco*I F | CATGCCATGGGCGACAAGCAGAAGAACGGCATC | pCB301-β_cYFP-TGB1_ | BSMV-based BiFC | *Nco*I restriction enzyme site |  |
| cYFP *Nco*I R | CATGCCATGGCCTTGTACAGCTCGTCCATGCC |  |  | *Nco*I restriction enzyme site |  |
| γb nYFP F | CTTCCGTTTCTAAGCCAATGGTGAGCAAGGGCGAGGA | pCB301-γ_γb-nYFP_ |  |  |  |
| γb nYFP R | CATTTTTTTTTTTTTTTAGGGCCCTTAGGCCATGATATAGACGT |  |  |  |  |
| QC TGB3-S18TAA F | CTTACCATCATaGGAATCATTTCCTATTTATGGCGAAC | pCB301-β_cYFP-TGB1mTGB3_ |  |  |  |
| QC TGB3-S18TAA R | GAAATGATTCCtATGATGGTAAGCATTGCGG |  |  |  |  |
| QCTGB2 1213TAA F | TCAAGGCCAAATAAGtaatgaCCAATTGTCGCCGGAATCGGTGTC | pCB301-β_cYFP-TGB1mTGB2_ |  |  |  |
| QCTGB2 1213TAA R | TCCGGCGACAATTGGtcattaCTTATTTGGCCTTGAACCAACTGTGGTC |  |  |  |  |
|  | | | | | |
| realtime αa_843_ F | GGTGCTTCCTGTTTCCTC |  | RT-qPCR |  |  |
| realtime αa_937_ R | AATCCTGCCATTCTCCAC |  |  |  |  |
| realtime PP2A F | GACCCTGATGTTGATGTTCGCT |  |  |  |  |
| realtime PP2A R | GAGGGATTTGAAGAGAGATTTC |  |  |  |  |
|  | | | | | |
| QC γb_I130G_ F | GAGGTTAACGCAGGACGTAAGTCCGTAGCTTC | pCB301-γ_I130Gdupflu_ | BSMV duplex  fluorescence system |  |  |
| QC γb_I130G_ R | CGGACTTACGTCCTGCGTTAACCTCACTTTC |  |  |  |  |
| QC γb_BM26_ F | GATGTGAGCAAAACCATGTATATTCTGAAACAAG | pCB301-γ_BM26dupflu_ |  |  |  |
| QC γb_BM26_ R | TATACATGGTTTTGCTCACATCTCTTACCACAGTA |  |  |  |  |
| QC mγb F | CCTTCGCTTGTTGGCTACTTTCTCTTGTGTGTGTT | pCB301-  γ_mγbdupflu_ |  |  |  |
| QC mγb R | GAAAGTAGCCAACAAGCGAAGGTAAATACAGT |  |  |  |  |
|  | | | | | |
| reversePCR PVX TGB1 GKS-3A R | CAGCGGCTCCGGCTACTGCATGTAC | pGDGm-PVXTGB1 pGDGm-PVXTGB1_6A_ | Subcellular localization |  |  |
| reversePCR PVX TGB1 GKS-3A F | CGGCCACAGCCCTAAGGAAGTTGATC |  |  |  |  |
| QC PVX TGB1 DE-AA F | CGCAATCCTCGCTGCGTATACTTTGGACAACACCACAAG |  |  |  |  |
| QC PVX TGB1 DE-AA R | CCAAAGTATACGCAGCGAGGATTGCGAAGTTGCCCTC |  |  |  |  |
| QC PVX TGB1 Q-A F | GACCCTTATGCGGCACCGGAGTTTAGCCTAGAGC |  |  |  |  |
| QC PVX TGB1 Q-A R | CCGGTGCCGCATAAGGGTCAGCAAAAAGTGC |  |  |  |  |
| infu PVX-TGB1 *Xho*I F | CTCTCTCTACAAGATCATGGATATTCTCATCAGTAGTTTGAAAAGTTTAG |  |  |  |  |
| infu PVX-TGB1 *Apa*I R | CTTTACTCATGGGCCCTGGCCCTGCGCGGACATATGTC |  |  |  |  |
| PVX-TGB23 *Xho*I F | CCGCTCGAGATGTCCGCGCAGGGCCATAG | pGD-PVXTGB2/3 |  | *Xho*I restriction enzyme site |  |
| PVX-TGB23 *Apa*I R | GGGGGCCCTCAATGGAAACTTAACCGTTCAACGGAGAGTG |  |  | *Apa*I restriction enzyme site |  |
| reversePCR BNYVV TGB1 GKS-3A R | CAGCAACACCAGGAGCACCCAAAACAATA | pGDG-BNYVVTGB1 pGDG-BNYVVTGB1_6A_ |  |  |  |
| reversePCR BNYVV TGB1 GKS-3A F | CAGCTACCTCGATTAAAAACTTGTTAGAC |  |  |  |  |
| QC BNYVV TGB1 DE-AA R | CACGAGTGACCGCAGCCACAAGCATGGTGTTATATTTTC |  |  |  |  |
| QC BNYVV TGB1 DE-AA F | CATGCTTGTGGCTGCGGTCACTCGTGTGCATATG |  |  |  |  |
| QC BNYVV TGB1 Q-A R | AATTCAACCCTGCCGCCGGATCACCAAAACATATC |  |  |  |  |
| QC BNYVV TGB1 Q-A F | GATCCGGCGGCAGGGTTGAATTATAAGGCCG |  |  |  |  |
| GFP-BNYVV TGB1 *Xho*I F | CCGCTCGAGCTATGGTCCAAGTACAGCGTAGAACGG |  |  | *Xho*I restriction enzyme site |  |
| GFP-BNYVV TGB1 *Apa*I R | GGGGGCCCTTATCTATCTTCGCAAAAGGTATCTCCGGTAC |  |  | *Apa*I restriction enzyme site |  |
| BNYVV TGB2 *Xho*I F | CCGCTCGAGATGTCTAGGGAAATAACCGCTCGACC | pGD-BNYVVTGB2/3 |  | *Xho*I restriction enzyme site |  |
| BNYVV TGB3 *Apa*I R | GGGGGCCCTTATCTATGATACCAAAACCAAACTATAGACATTAAACATATCATGAAC |  |  | *Apa*I restriction enzyme site |  |
| QC BSMVTGB1 DE-AA F | CATAGCTGCATATACACTTGCTGAGAGCGCGGAAATCC | pCB301-βGFP-TGB1_6A_  pGDG-BSMVTGB1_6A_ |  |  |  |
| QC BSMVTGB1 DE-AA R | GTGTATATGCAGCTATGATCAATAAATCAGACGTTATCG |  |  |  |  |
| QC BSMVTGB1 Q343A F | GGATGTAGCTGCAGGAAAAGCCACCACTGCTTCCAG |  |  |  |  |
| QC BSMVTGB1 Q343A R | CTTTTCCTGCAGCTACATCCCCGACTAACAACACCATAG |  |  |  |  |
| reversePCR BSMVTGB1GKS-3A R | GCTGCTGAGCCAGGAACTCCAGAGATGATTC |  |  |  |  |
| reversePCR BSMVTGB1GKS-3A F | CGCAACCATTGTGCGTACTTTGCTCAAAGGTG |  |  |  |  |
| BSMV TGB1 *Xho*I F | CCGCTCGAGATGGACATGACGAAAACTGTTG |  |  | *Xho*I restriction enzyme site |  |
| BSMV TGB1 *Apa*I R | GGGGGCCCTTTGGCCTTGAACCAACTGTGGTCTT |  |  | *Apa*I restriction enzyme site |  |
|  | | | | | |
| T7+kozak+BSMVγb | ATACGACTCACTATAGGGAGCCACCATGATGGCTACTTTCTCTTGTGTGTGTTG | γb and γb_86-152_ templates | *In vitro* translation | T7 promoter + spacer (AG) + Kozak sequence |  |
| T7+kozak+BSMVγb86 | ATACGACTCACTATAGGGAGCCACCATGGCGGATCTGTATGATTCACTTCTGAAACG |  |  |  |  |
| BS32 | TGGTCTTCCCTTGGGGGAC |  |  |  |  |
| T7+kozak TGB1-F | ATACGACTCACTATAGGGAGCCACCATGGACTACAAAGACCATGACGGTG | 3xFlag-TGB1 template |  |  |  |
| BSMV TGB1 R | TTATTTGGCCTTGAACCAACTGTG |  |  |  |  |
| M13-47 | CGCCAGGGTTTTCCCAGTCACGAC | TGB2/3 template |  |  |  |
| BSMV TGB3-R | TTACCTTTTTGAAGAAAGTAAGAGAAAAAAGCTTAAC |  |  |  |  |
