# Supplementary material for: The Barley stripe mosaic virus γb protein promotes viral cell-to-cell movement by enhancing ATPase-mediated assembly of ribonucleoprotein movement complexes
Source: PLoS Pathog. 2020 Jul 30;16(7):e1008709. doi: 10.1371/journal.ppat.1008709 (PMC7419011; doi:10.1371/journal.ppat.1008709)
Supplement: S2 Table — Host proteins identified by LC-MS/MS after immunoprecipitation of 3xFlag-TGB1 proteins from BSMVmCP/3xFlag-TGB1-infected N. benthamiana. (DOCX) [file ppat.1008709.s002.docx]

**S2 Table.** Host proteins identified by LC-MS/MS after immunoprecipitation of 3xFlag-TGB1 from BSMV_mCP/3xFlag-TGB1_-infected *N. benthamiana.*

| GenBank  Accession No. | Score ^a^ | Mass  (Dalton) | Number  of matches | Number  of significant  matches | Number of  sequences | Number of  significant  sequences | emPAI | Sequence  coverage  (%) | Description |
| --- | --- | --- | --- | --- | --- | --- | --- | --- | --- |
| gi\|12643757 | 6756 | 48951 | 202 | 166 | 22 | 19 | 8.38 | 0.64 | RuBisCO activase 1 |
| gi\|697184955 | 6961 | 52771 | 300 | 223 | 24 | 22 | 15.43 | 0.66 | Elongation factor TuB,  chloroplastic |
| gi\|698587115 | 5228 | 52152 | 205 | 157 | 22 | 20 | 9.42 | 0.61 | Elongation factor TuA,  chloroplastic |
| gi\|698475586 | 1783 | 71459 | 66 | 50 | 23 | 20 | 2.46 | 0.54 | Hsc70-2 |
| gi\|110227074 | 950 | 39766 | 41 | 27 | 7 | 5 | 1.33 | 0.26 | photosystem II protein D2 |
| gi\|225425567 | 1301 | 20752 | 42 | 31 | 10 | 8 | 6.45 | 0.74 | ADP-ribosylation factor 2 |
| gi\|685295726 | 1242 | 20653 | 40 | 31 | 10 | 8 | 6.53 | 0.73 | ADP-ribosylation factor 1 |
| gi\|294440432 | 1115 | 43064 | 37 | 25 | 12 | 9 | 1.66 | 0.46 | Plastidic aldolase |
| gi\|698496378 | 792 | 40566 | 48 | 27 | 17 | 8 | 1.29 | 0.51 | Ferredoxin--NADP  reductase |
| gi\|697180030 | 599 | 138173 | 38 | 26 | 21 | 14 | 0.54 | 0.23 | Coatomer subunit alpha-1-  like |
| gi\|697162111 | 243 | 43678 | 19 | 14 | 9 | 6 | 0.78 | 0.33 | S-adenosylmethionine  synthase 1 |
| gi\|84620804 | 266 | 29238 | 8 | 6 | 5 | 4 | 0.78 | 0.2 | chloroplast photosystem II  22 kDa component |
| gi\|61697113 | 204 | 28472 | 3 | 3 | 3 | 3 | 0.55 | 0.18 | chloroplast photosynthetic  oxygen-evolving protein  23 kDa subunit |
| gi\|415666345 | 176 | 41957 | 11 | 9 | 7 | 6 | 0.83 | 0.24 | calcium-sensing receptor |
| gi\|697141344 | 119 | 57367 | 10 | 5 | 4 | 3 | 0.25 | 0.1 | Catalase isozyme 3 |

Individual ions scores > 4 indicate identity or extensive homology (*p* < 0.05).
