# Supplementary material for: The Barley stripe mosaic virus γb protein promotes viral cell-to-cell movement by enhancing ATPase-mediated assembly of ribonucleoprotein movement complexes
Source: PLoS Pathog. 2020 Jul 30;16(7):e1008709. doi: 10.1371/journal.ppat.1008709 (PMC7419011; doi:10.1371/journal.ppat.1008709)
Supplement: S3 Table — Host proteins identified by LC-MS/MS after immunoprecipitation of γb-3xFlag proteins from BSMVγb-3xFlag-infected N. benthamiana. (DOCX) [file ppat.1008709.s003.docx]

**S3 Table.** Host proteins identified by LC-MS/MS after immunoprecipitation of γb-3xFlag from BSMV_γb-3xFlag_-infected *N. benthamiana.*

| GenBank  Accession No. | Score ^a^ | Mass  (Dalton) | Number of  matches | Number of  significant  matches | Number of  sequences | Number of  significant  sequences | emPAI | Description |
| --- | --- | --- | --- | --- | --- | --- | --- | --- |
| gi\|698510587 | 6731 | 42928 | 228 | 172 | 18 | 17 | 4.85 | Glyceraldehyde-3-phosphate  dehydrogenase A |
| gi\|37721383 | 6254 | 55015 | 204 | 147 | 15 | 12 | 1.93 | Photosystem II CP47 protein |
| gi\|395001670 | 3354 | 39838 | 120 | 80 | 8 | 6 | 1.33 | Photosystem II D2 protein |
| gi\|290488500 | 2542 | 50480 | 107 | 81 | 13 | 9 | 1.51 | Photosystem II CP43 chlorophyll  apoprotein |
| gi\|972776542 | 3930 | 29661 | 141 | 118 | 9 | 9 | 3.75 | Chlorophyll a-b binding protein 4 |
| gi\|21633399 | 2506 | 52938 | 123 | 87 | 20 | 18 | 4.78 | ATP synthase beta subunit |
| gi\|301353343 | 856 | 81980 | 25 | 22 | 5 | 5 | 0.29 | Photosystem I P700 apoprotein A2 |
| gi\|723446999 | 170 | 44787 | 13 | 8 | 4 | 4 | 0.6 | Translational elongation factor Tu |
| gi\|335059551 | 1215 | 51918 | 160 | 71 | 16 | 11 | 1.65 | Ribulose-1,5-bisphosphate  carboxylase/oxygenase large subunit |
| gi\|697175371 | 1487 | 80314 | 87 | 64 | 20 | 16 | 1.32 | Transketolase |
| gi\|700583762 | 518 | 15204 | 29 | 18 | 6 | 3 | 1.26 | Photosystem I reaction center subunit  IV A |
| gi\|698435947 | 833 | 34872 | 39 | 26 | 11 | 8 | 1.63 | Carbonic anhydrase |
| gi\|460382334 | 61 | 38140 | 3 | 3 | 1 | 1 | 0.12 | Outer envelope pore protein 37 |

Individual ions scores > 27 indicate identity or extensive homology (*p* < 0.05).
