# Supplementary figures and images for: The Barley stripe mosaic virus γb protein promotes viral cell-to-cell movement by enhancing ATPase-mediated assembly of ribonucleoprotein movement complexes
Source: PLoS Pathog. 2020 Jul 30;16(7):e1008709. doi: 10.1371/journal.ppat.1008709 (PMC7419011; doi:10.1371/journal.ppat.1008709)

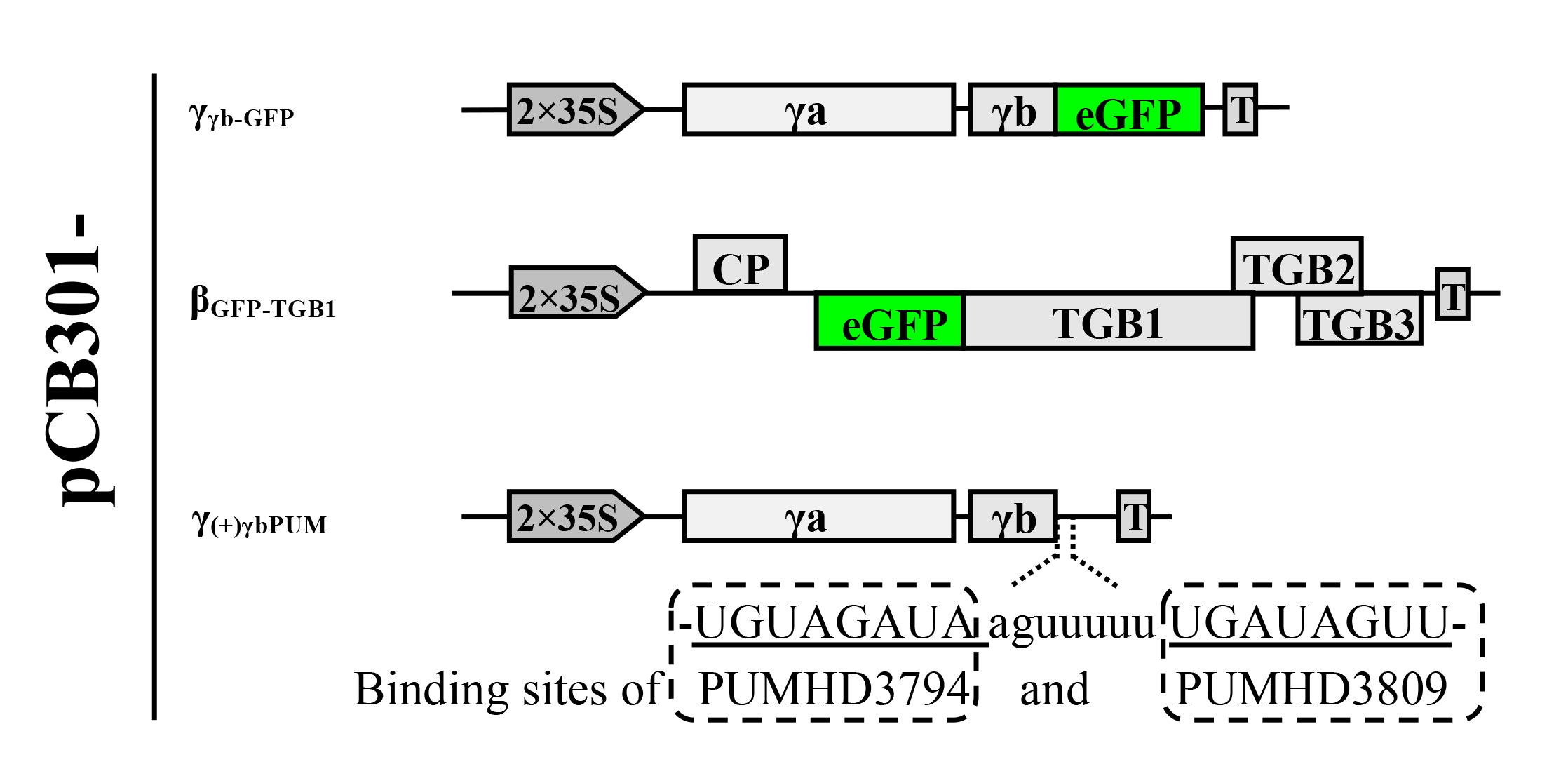

Supplement: S1 Fig — BSMV fluorescent reporter constructs used in this study. (TIF) [file ppat.1008709.s004.tif]

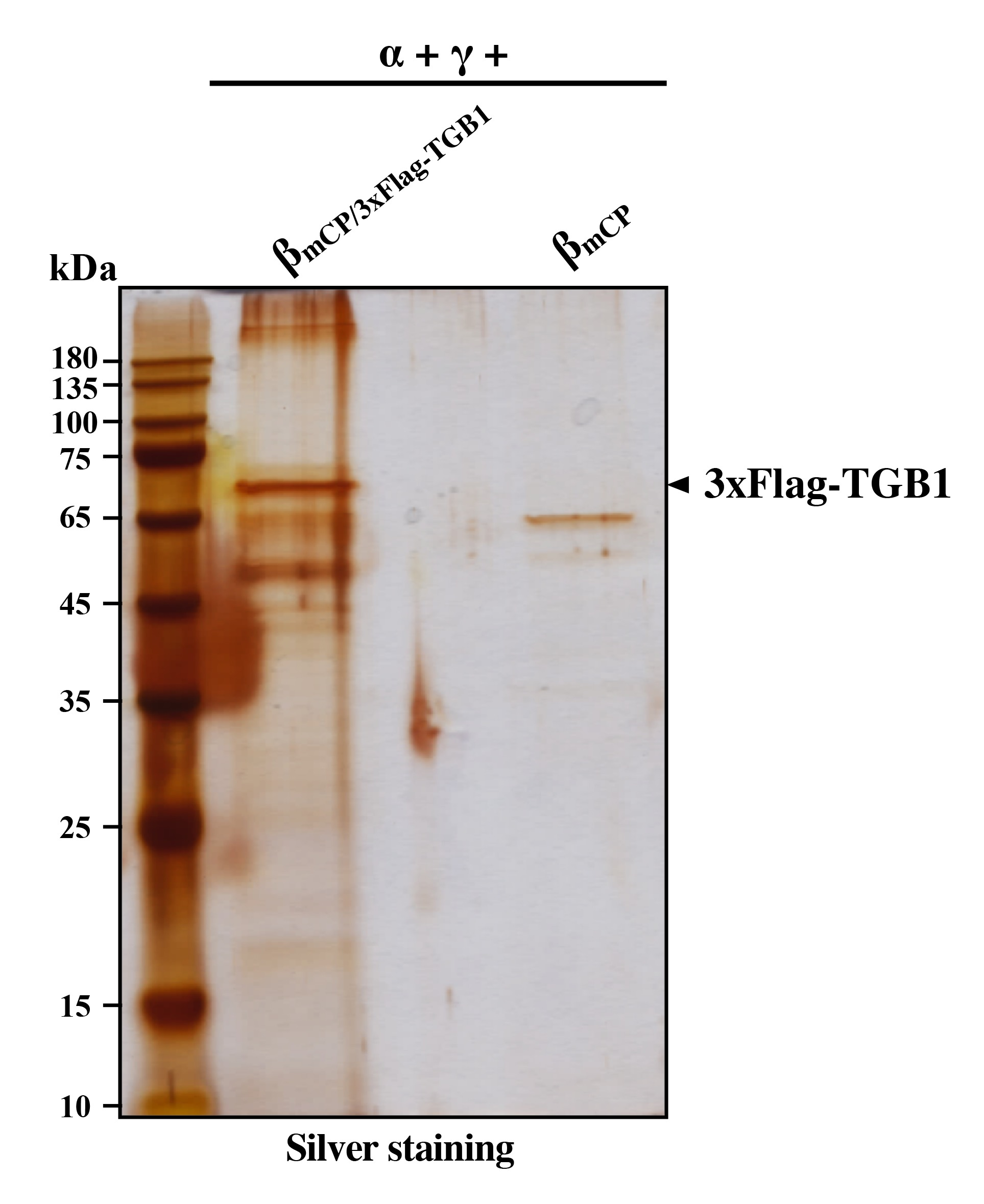

Supplement: S2 Fig — A. tumefaciens containing plasmids expressing RNAα, RNAβmCP/3xFlag-TGB1 or RNAγ were co-infiltrated into N. benthamiana leaves. After immunoprecipitation with anti-FLAG beads, recovered IP products were analyzed by SDS-PAGE silver staining. The BSMVmCP serves as a negative control. (TIF) [file ppat.1008709.s005.tif]

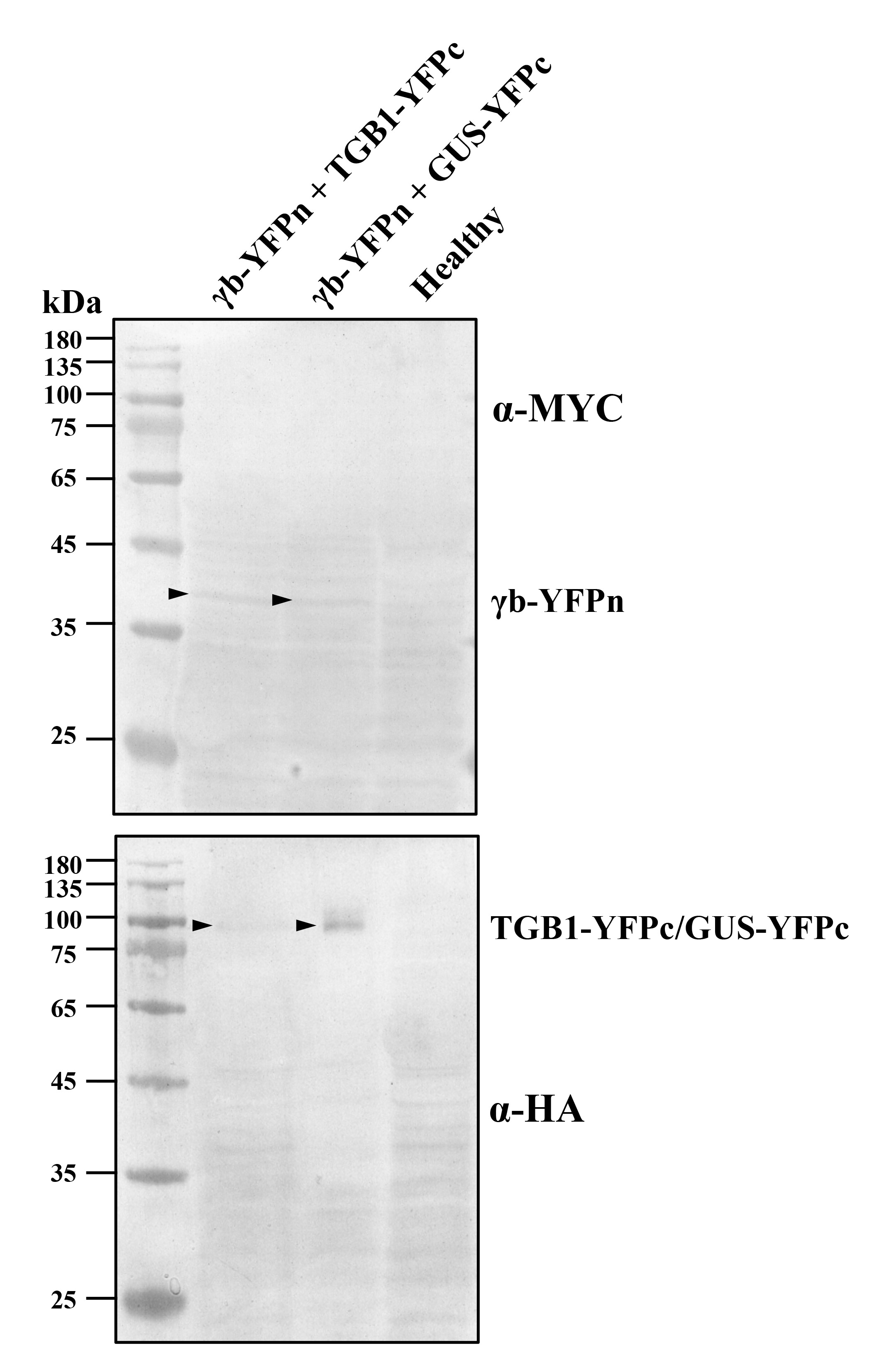

Supplement: S3 Fig — The predicted molecular weight of TGB1 is about 57 kDa, but the protein migrates slower than predicted with an apparent size of ~68 kDa; hence the molecular weight of TGB1-YFPc appears to be ~80 kDa, which is comparable to that of the GUS-YFPc negative control. Similar increases of the apparent TGB1 size were also observed in our previous studies [21]. The γb-YFPn protein is ~37 kDa. Uninfiltrated healthy leaves (Healthy) serve as negative controls for Western blot analyses. Sizes (in kDa) of molecular weight markers are shown on the left and antibodies used for detection are indicated on the right, arrowheads indicate the target protein bands. (TIF) [file ppat.1008709.s006.tif]

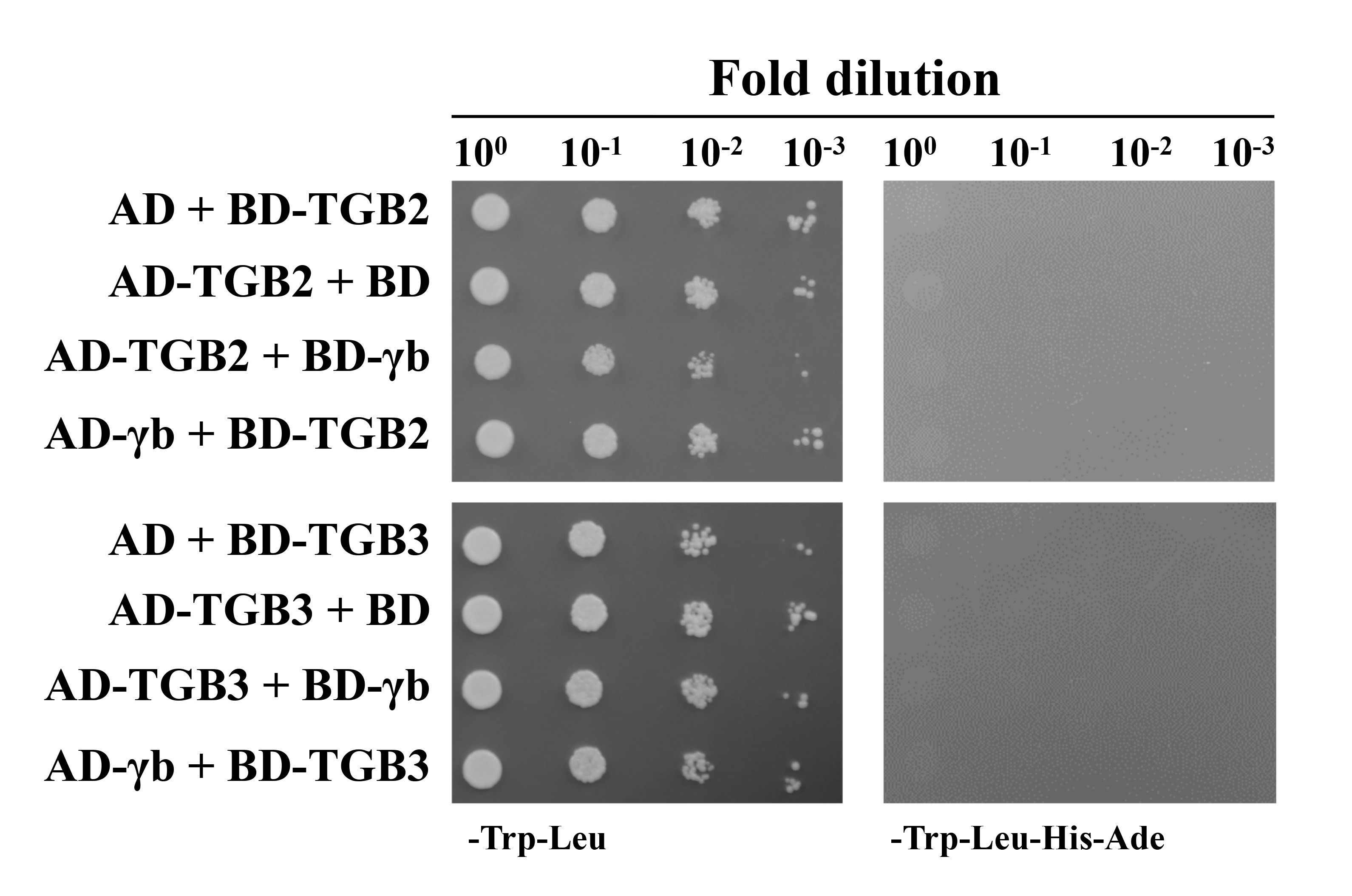

Supplement: S4 Fig — Yeast cells transformed with plasmids indicated on the left were pipetted onto synthetic dextrose dropout media (SD/-Trp-Leu or SD/-Trp-Leu-His-Ade) in a series of 10-fold dilutions. The Y2H combinations containing either empty AD or BD constructs were used as negative controls. (TIF) [file ppat.1008709.s007.tif]

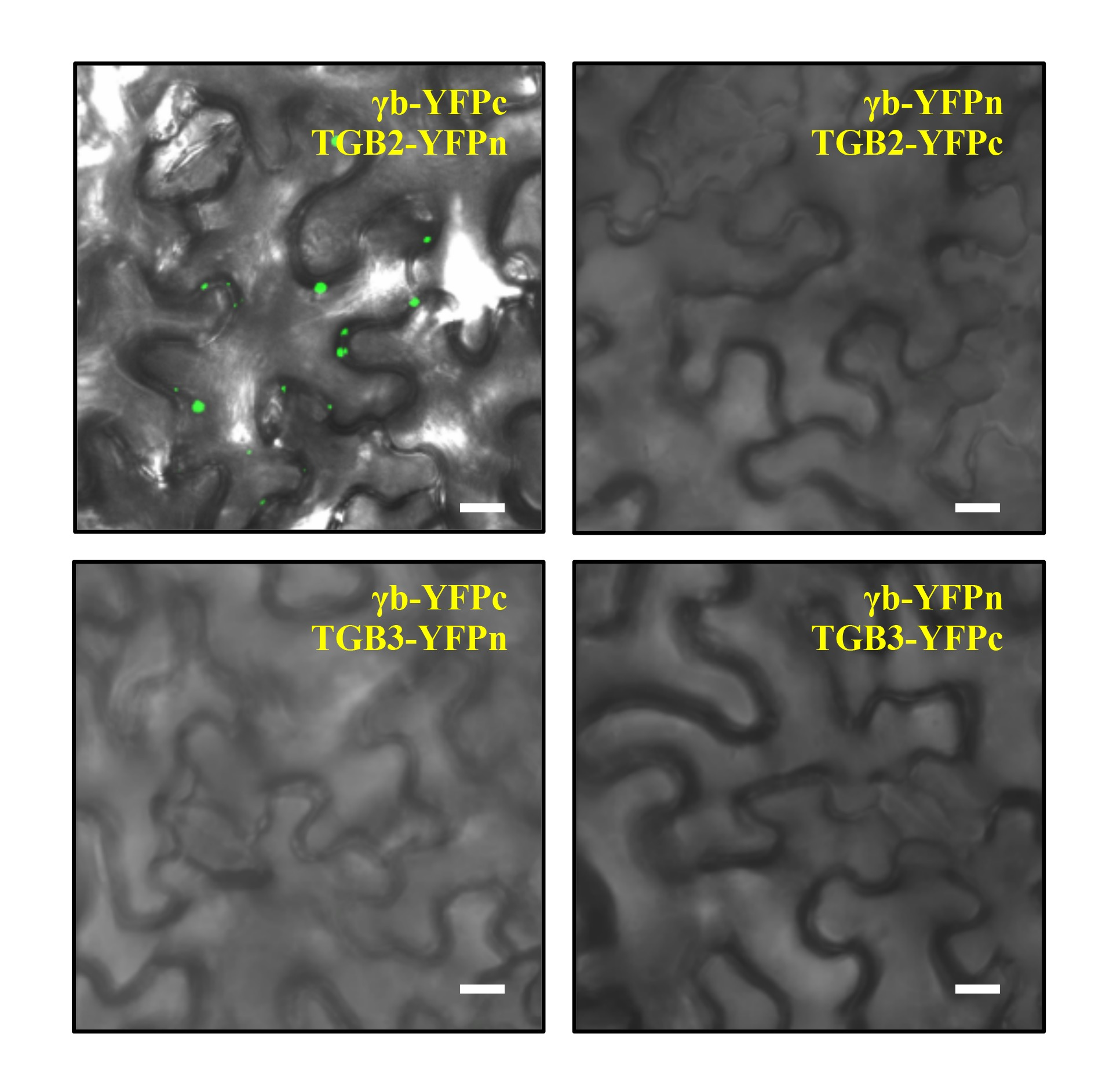

Supplement: S5 Fig — Confocal microscopy of BiFC assays to investigate γb interactions with TGB2 or TGB3 in N. benthamiana epidermal cells at 3 dpi. Scale bars, 10 μm. (TIF) [file ppat.1008709.s008.tif]

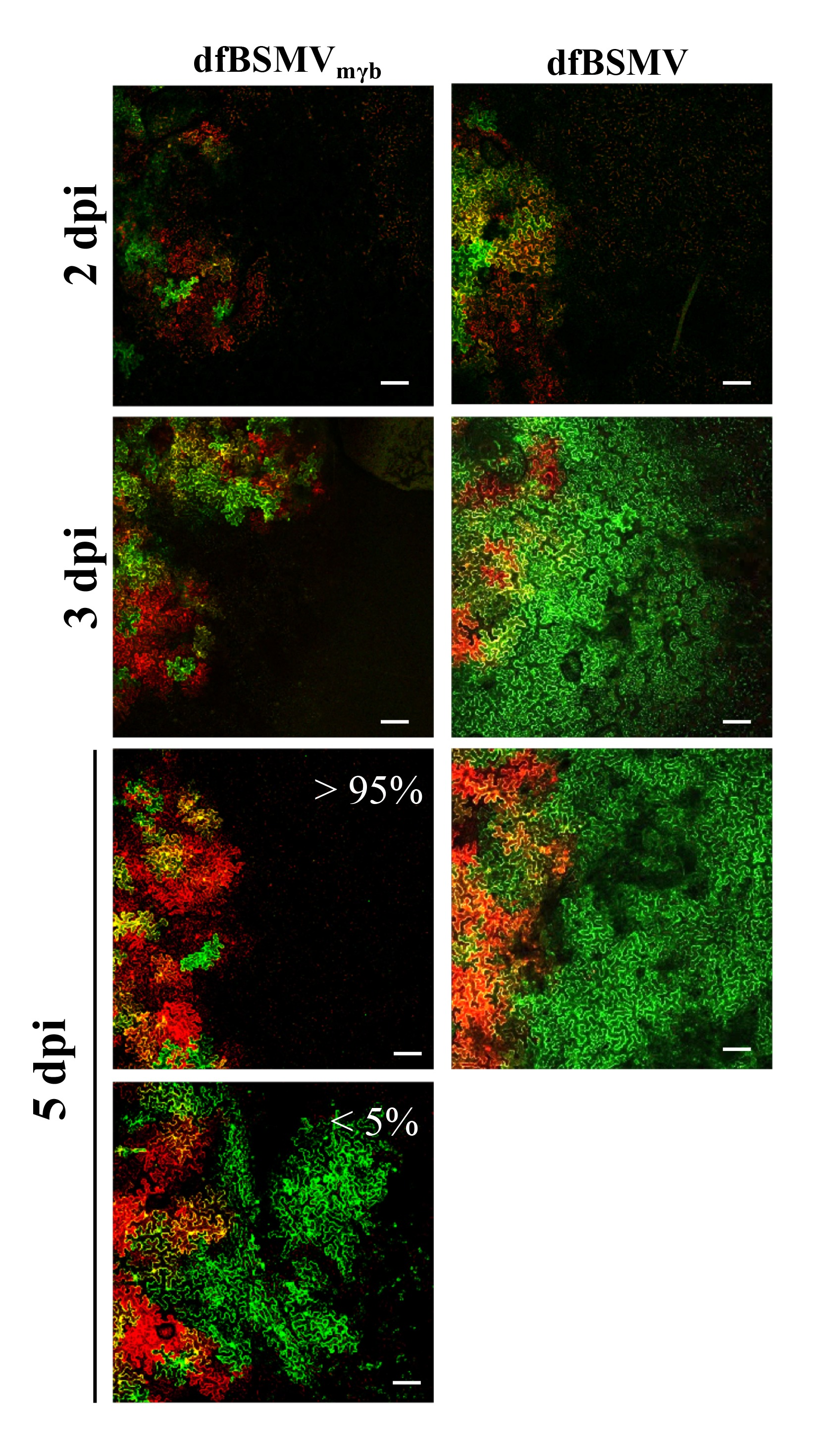

Supplement: S6 Fig — Representative confocal images of N. benthamiana epidermal cells after infiltration with A. tumefaciens containing different BSMV derivatives at 2 dpi, 3 dpi, and 5 dpi, respectively. The percentage in the upper right of the image indicates the proportion of such case among the observed samples. At least five individual leaf sections were visualized at each time point. Scale bars, 100 μm. (TIF) [file ppat.1008709.s009.tif]

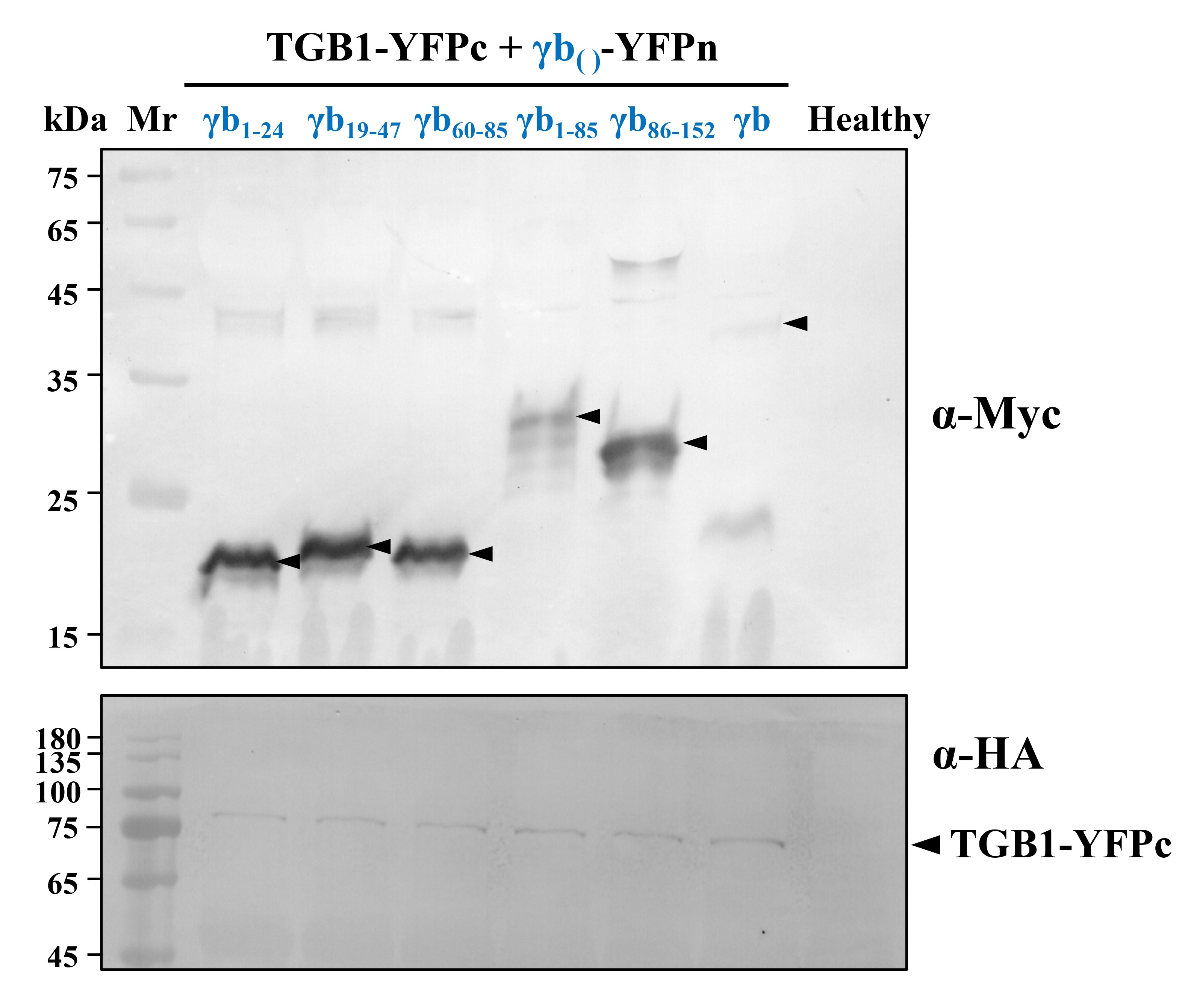

Supplement: S7 Fig — The molecular weights of γb1-24-YFPn, γb19-47-YFPn, γb60-85-YFPn, γb1-85-YFPn and γb86-152-YFPn are about 22 kDa, 23 kDa, 22 kDa, 29 kDa and 27 kDa, respectively. Non-infiltrated healthy leaves (Healthy) serve as a negative control. (TIF) [file ppat.1008709.s010.tif]

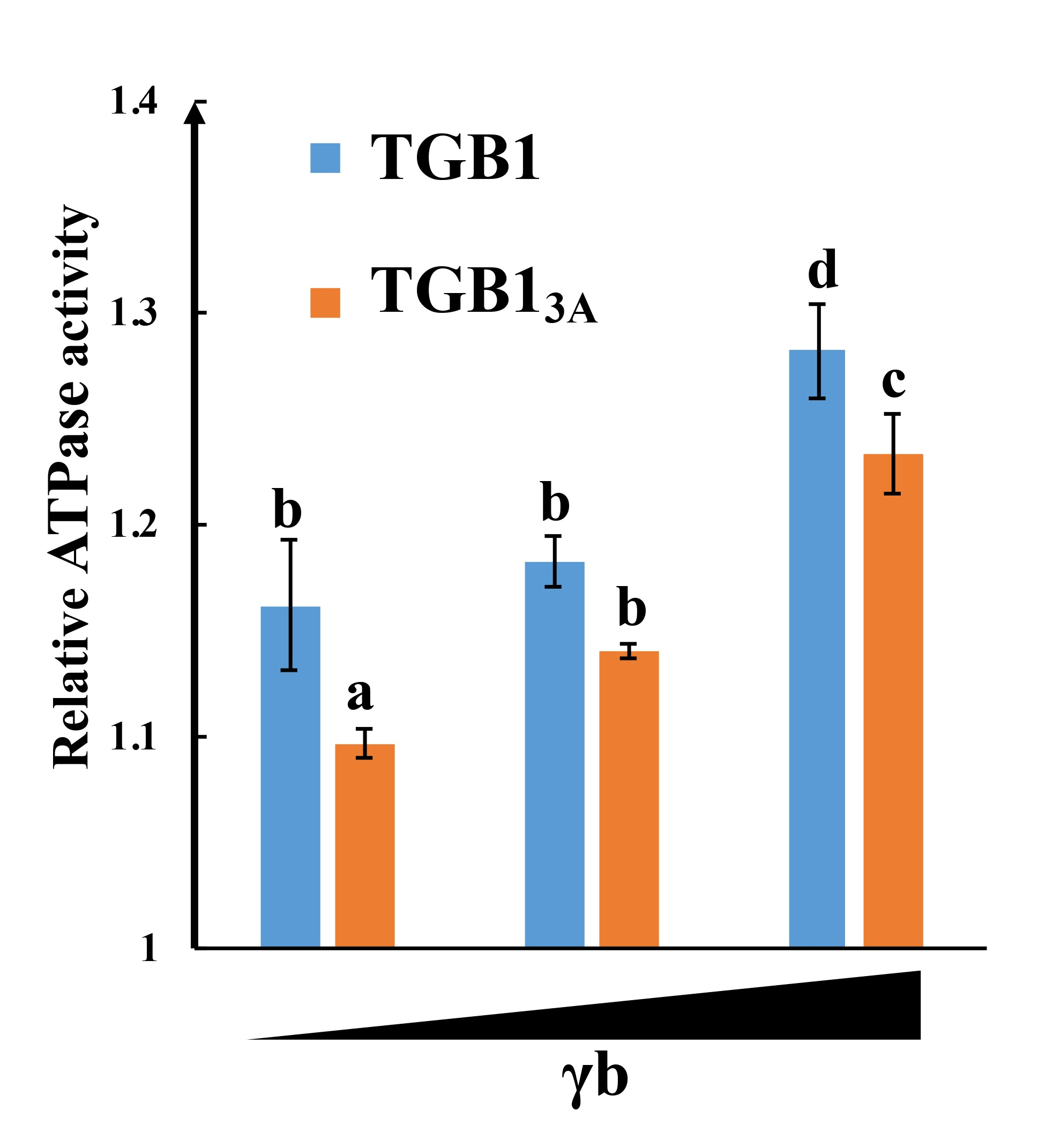

Supplement: S8 Fig — The TGB13A-His protein was incubated with increasing amounts of GST-γb and subjected to ATPase assays and A620 values were accessed spectrophotometrically. The letters above each bar show statistically significant differences (P < 0.05) determined by Duncan’s multiple range test (n = 2). (TIF) [file ppat.1008709.s011.tif]

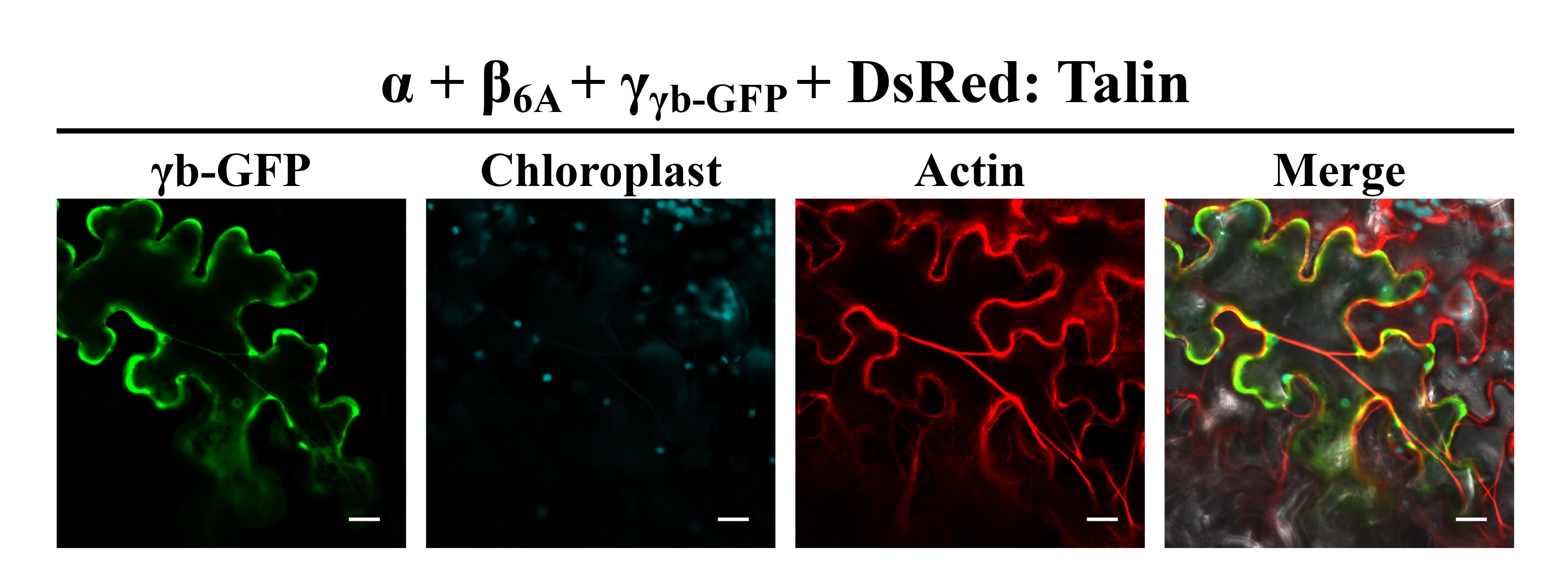

Supplement: S9 Fig — A. tumefaciens containing plasmids expressing RNAα, RNAβ6A, RNAγγb-GFP or DsRed: Talin were co-infiltrated into N. benthamiana leaves and the epidermal cells were observed at 3 dpi by confocal microscopy. Scale bar, 20 μm. Chloroplasts are displayed as a false blue color. (TIF) [file ppat.1008709.s012.tif]
